# SUMOylated DDX3 Promotes HBV Replication and Tumor Growth of Hepatocellular Carcinoma via TRIM28‐DDX3‐KLF4α Signaling Axis

**DOI:** 10.1002/advs.78603

**Published:** 2026-11-03

**Authors:** Yanfang Yang, Ting Kong, Binghan Wang, Xiaoli Jin, Lan Huang, Nan Zhang, Siyu Tang, Samina Ejaz Syed, Shufang Liang

**Affiliations:** ^1^ Department of Biotherapy Cancer Center and State Key Laboratory of Biotherapy West China Hospital Sichuan University Chengdu China; ^2^ Department of Central Laboratory NHC Key Laboratory of Pulmonary Immunological Diseases Guizhou Provincial People's Hospital Guiyang China; ^3^ Department of Neurology Second Affiliated Hospital of Army Medical University (Xinqiao Hospital) Chongqing China; ^4^ Department of Medical Oncology Cancer Center West China Hospital Sichuan University Chengdu China; ^5^ Department of Gastrointestinal Surgery West China Hospital Sichuan University Chengdu China; ^6^ The Second Department of Gastrointestinal Surgery the Affiliated Hospital of North Sichuan Medical College Sichuan China; ^7^ Department of Biochemistry and Biotechnology Baghdad Campus The Islamia University of Bahawalpur Bahawalpur Pakistan

**Keywords:** DDX3, deSUMOylation, HBV‐associated hepatocellular carcinoma, Hepatitis B virus, SUMOylation

## Abstract

DDX3 has dual biological functions of regulating cell proliferation and antiviral effects. Herein, we focus on SUMOylation‐mediated DDX3 in the interplay from hepatitis B virus (HBV) infection to HBV‐associated hepatocellular carcinoma (HBV‐HCC) progression. We identified dynamic regulation of SUMOylation and DeSUMOylation of DDX3 at four key lysine (K) residues (K81/K208/K215/K564) in response to HBV infection. During the early stage of HBV acute infection, SENP1 interacts with DDX3 to keep DDX3 deSUMOylation for inhibiting HBV replication. During persistent HBV replication, as SENP1 downregulation and HBV polymerase accumulation, DDX3 disassociates with SENP1 to bind with TRIM28, which induces DDX3 SUMOylation. Meanwhile, SUMOylated DDX3 enhances alternative splicing of *KLF4* mRNAs to generate KLF4α isoform that involves in Cyclin D1‐mediated cell cycle progression, weakens tumor suppressor function of DDX3 and promotes HBV‐HCC. Moreover, DDX3 expression is positively correlated with *KLF4* mRNA, and high DDX3 level with low TRIM28 expression indicates a better overall survival for HBV‐HCC patients. Our findings reveal the dynamic regulation between SUMOylation and deSUMOylation of DDX3, including mediating HBV gene transcription, virus antigen secretion, and cell proliferation. Furthermore, the TRIM28‐DDX3‐KLF4 axis as a novel therapeutic target for HBV‐HCC, exhibiting the dual ability to inhibit tumor progression and disrupt the HBV–host interaction.

## Graphical abstract

TRIM28 binds to DDX3 protein to catalyze the SUMOylation of DDX3 (S‐DDX3), thus the S‐DDX3 involves in aberrant alternative splicing of *KLF4* mRNA, and the splicing variant *KLF*4α‐mediated cell cycle and signal transduction, weakens tumor suppressor function of DDX3 protein, which promotes progression of HBV‐HCC. Conversely, SENP1 interacts with DDX3 to keep DDX3 deSUMOylation that is essential for inhibiting HBV gene transcription and HBsAg secretion.

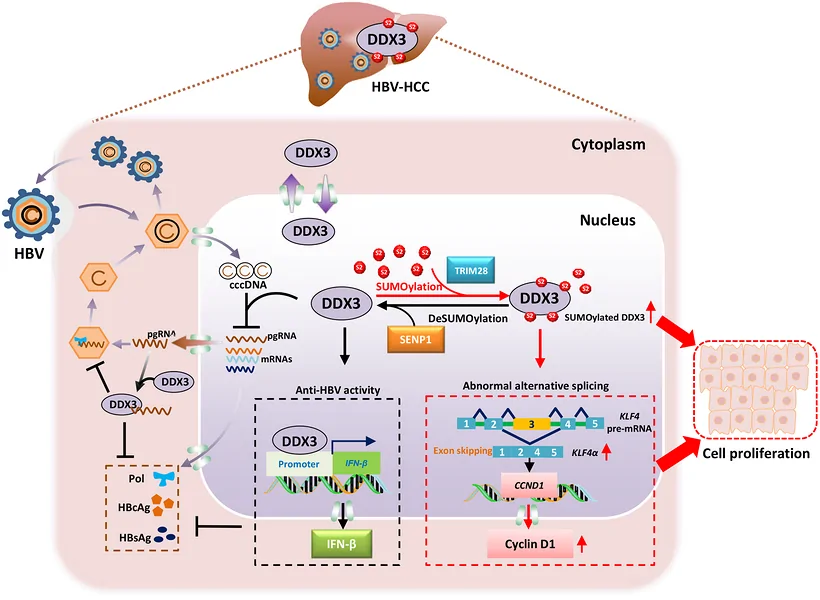

## 1 Introduction

Hepatitis B virus (HBV) is an enveloped DNA virus with a 3.2‐kb circular, partially double‐stranded DNA genome. Despite the availability of a vaccine against HBV has been developed, there are still 296 million people worldwide chronically infected with the hepatotropic virus. HBV infection usually causes hepatitis, and long‐term chronic hepatitis would lead to cirrhosis of the liver, finally inducing carcinogenesis of HBV‐associated hepatocellular carcinoma (HCC) [1]. HBV‐associated HCC (HBV‐HCC) accounts for approximately 85% of HCC cases in China [2]. Treatment of HBV‐HCC mainly includes antiviral therapy before and after tumor treatment. The purpose of anti‐HBV therapy is to eliminate HBV, prevent the progression to cirrhosis, and reverse cirrhosis, which reduces tumor occurrence of HBV‐HCC, improves liver function, tolerates tumor treatment and significantly prevents tumor recurrence after treatment. HBV replication has an impact on the postoperative rehabilitation of HCC patients, and it is related to the tumor node metastasis (TNM) staging and disease progression of HBV‐HCC [3].

Interdiction of HBV replication, transcription, and translation not only relies on various viral elements but also depends on host factors. Post‐translational modifications (PTMs) of host cell proteins are concerned to involve in regulating progression of HBV‐HCC [4, 5, 6], which provides a novel viewpoint to explore HBV‐related HCC carcinogenesis mechanism. For instance, the SUMOylated CPAP protein is required for IKK‐mediated NF‐κB activation and enhances HBx‐induced NF‐κB signaling in HCC [6]. Lysine crotonylation takes participation in HCC progression, which is enriched on active gene promoters or potential enhancers in mammalian cell genomes [7].

Small ubiquitin‐like modifier (SUMO) modification, SUMOylation, is one type of PTMs to regulate function of a large number of proteins and biological processes [8, 9, 10, 11]. Viruses interact with the mammalian cell SUMO pathway to promote infection, persistence, and malignant transformation of host cells [5]. SUMOylation of host cell proteins contributes to HBV gene transcription and packaging, thus enhancing the replication ability of HBV virus [5, 12]. p65 is one of the most important subunits of NF‐κB, and p65 interacts with SUMO2/3 for stabilizing in cytoplasm to involve in the transformation of chronic hepatitis B into HCC [13]. With difference of host transcription factor Sp110 modified by SUMO1 under physiological conditions, in the presence of HBV, Sp110 is deSUMOylated and dissociated from the promyelocytic leukemia nuclear bodies complex to regulate genes related to the type I interferon pathway, which is conducive to HBV proliferation [14]. Considering SUMOylation‐modulated function and significance, targeting of the SUMOylation pathway serves as a promising effective approach for treatment of HBV‐HCC [15].

Human X‐linked DEAD (Asp‐Glu‐Ala‐Asp) box helicase 3 (DDX3) is a member of the DEAD‐box family of RNA helicases, which has been unraveled in cancer progression, including the oncogenic and tumor suppressive capability in regulating cancer proliferation, metastasis, and drug resistance [16]. DDX3 promotes cell proliferation and migration of HCC cells by enhancing its stability or interacting with HBV polymerase (Pol) [17]. Evidences have shown that functional complexity of DDX3 in cancers is determined by its interacting targets and is tumor‐dependent or context‐dependent.

The previous reports show DDX3 has a double‐edged even contradictory role in HBV infection process. DDX3 plays an important role in inhibiting HBV transcription and replication as a host cell element [18]. When HBV infects and enters into hepatocytes, DDX3 serves as a host cytokine to inhibit virus replication through innate immune response [19]. During early stage of HBV infection, DDX3 protein interacts with IKK/TBK1, and binds to the interferon (IFN) promoter to activate the transcription of IFN, which finally induces IFN expression for inhibiting HBV [20]. Viral DNA enters the host cell nucleus, DDX3 shuttles into hepatocyte nucleus to inhibit HBV cccDNA transcription, reduces HBV RNA level in an ATPase activity‐dependent manner, which specifically suppresses HBV genome replication at the transcription level [18]. While on the other hand, with the increase of HBV virus products accumulated in host cells, the antiviral function of DDX3 is gradually weakened. HBV Pol would compete with TBK1/IKKε to bind to host DDX3 protein, interferes with the activation of IRF3, and inhibits production of IFN‐β mediated by RIG‐I and TLR3 [17, 21]. What specific factor or mechanism confers DDX3 converse roles in response to HBV existence?

Focusing on the two coexistent and interacting biological processes between HBV replication and host HCC cell proliferation, herein we have identified DDX3 as a SUMOylation substrate related to HBV infection and HBV‐HCC progression, and have further explored the molecular mechanism of SUMOylation‐mediated DDX3 in response to HBV replication and regulating HBV‐HCC cellular biological processes. Mechanistically, SENP1‐mediated deSUMOylation state of DDX3, and DDX3 interacts with HBV replication intermediate pgRNA and HBV Pol to inhibit HBV gene transcription and HBsAg secretion. Conversely, TRIM28 binds to DDX3 protein to catalyze SUMOylation of DDX3, thus the SUMOylated DDX3 involves in aberrant alternative splicing of *KLF4* mRNA, and the splicing variant KLF4α‐mediated cell cycle and signal transduction, weakens anti‐HBV activity and tumor suppressor function of DDX3 protein, which promotes progression of HBV‐HCC. Our findings indicate targeting TRIM28‐DDX3‐KLF4 signaling axis is a potential effective strategy for treatment of HBV‐HCC.

## 2 Results

### 2.1 DDX3 Prefers to Covalently Conjugate With SUMO2 in HCC Cells

SUMOylation has emerged as a key PTM for regulating protein–protein interactions, transport, and transcription activities, thus modulating a variety of biological processes [22]. We detected the subcellular localization of SUMO1/2/3 and DDX3 in HCC cells to confirm whether DDX3 would be conjugated with SUMOs. Double‐labeled immunofluorescent assay showed that the endogenous SUMOs (SUMO1, SUMO2 and SUMO3) were co‐localized with DDX3 in nucleus of HepG2 and Hep3B cells (Figure 1A), which indicated DDX3 interacted with the SUMO molecules in cell nucleus.

**FIGURE 1:**
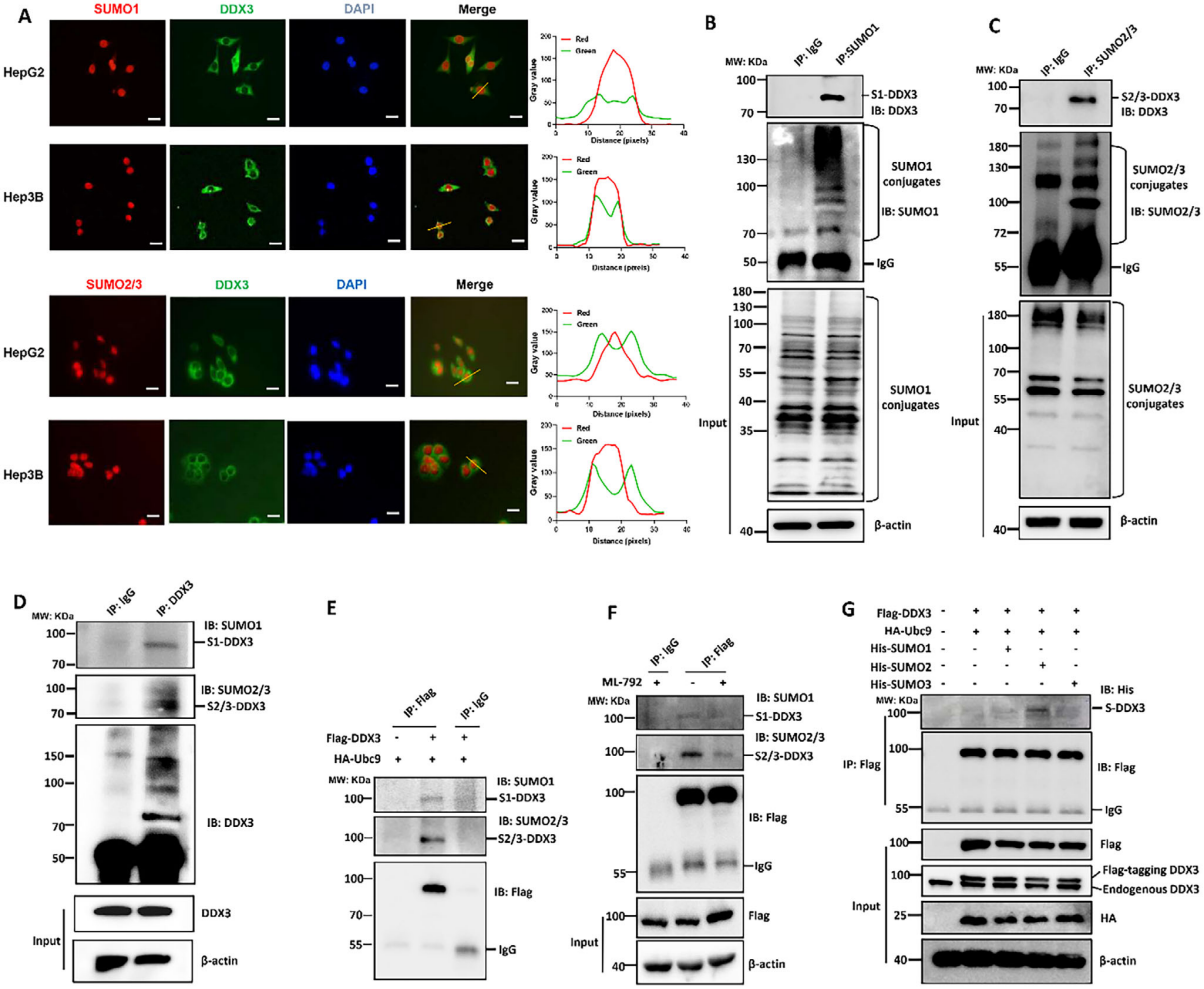
DDX3 is mainly SUMOylated with SUMO2 conjugation in HCC cells. (A) The co‐localization of DDX3 and SUMO1/2/3 were detected in HepG2 and Hep3B cells by immunofluorescence. The yellow lines indicate the position of fluorescent intensity profiles in HepG2 and Hep3B cells. Scale bars represent 20 µm. (B–D) DDX3 was modified by cell endogenous SUMO1/2/3 in HCC cells. HepG2 cells were lysed under denaturing condition, DDX3‐binding protein complex was enriched by IP respectively with anti‐DDX3, anti‐SUMO1 or anti‐SUMO2/3 antibodies, and western blotting with anti‐DDX3, anti‐SUMO1 and anti‐SUMO2/3 antibodies. As a negative control, the primary antibody was replaced with the normal IgG to eliminate the nonspecific protein binding. (E) The SUMO1/2/3‐modified DDX3 was detectable in the exogenous Flag‐tagging DDX3 protein. HepG2 cells were transfected with pFlag‐DDX3 with or without pHA‐Ubc9. Cell lysates were IP with anti‐Flag antibody or IgG, followed by immunoblotting using anti‐Flag, anti‐SUMO1and anti‐SUMO2/3 antibodies. (F) ML‐792 decreased the SUMOylation of DDX3 in HepG2 cells. HepG2 cells were co‐transfected with the pFlag‐DDX3 and pHA‐Ubc9 for 24 h, then treated with 0.5 µM ML‐792 for 48 h, the cells were lysed and analyzed by IP using anti‐IgG or anti‐Flag antibody followed by Western blotting. (G) DDX3 was mainly SUMOylated with SUMO2 conjugation, compared with the binding level with SUMO1 and SUMO3. HepG2 cells were co‐transfected with pFlag‐DDX3 and pHis‐SUMO1‐3, then the cells were lysed and the proteins analyzed by IP and Western blotting using different antibodies. S1‐DDX3: SUMO1 modified DDX3. S2/3‐DDX3: SUMO2/3 modified DDX3. IP: immunoprecipitation; IB: immunoblot; Input: the total cell lysate.

Moreover, cell lysates from HepG2 cells were subjected to enrich SUMO‐binding proteins by immunoprecipitation (IP) using SUMO1 and SUMO2/3 antibodies, and the protein complexes were immunoblotted with the anti‐DDX3, anti‐SUMO1, and anti‐SUMO2/3 antibodies, respectively. Meanwhile, the nonspecific binding via IgG antibody in IP was taken as the negative control. Compared with the native protein position of DDX3 around 75‐KDa, the endogenous SUMOylated DDX3 was visible along with multiple slower migration bands with molecular weight over 75 KDa, which showed DDX3 were conjugated with ∼12 KDa of SUMO1(S1‐DDX3) or SUMO2/3 (S2/3‐DDX3) in HCC cells. (Figure 1B–D).

We further validated that if the exogenous plasmid co‐transfection of pTango‐4Flag ‐DDX3 (pFlag‐DDX3, the measured molecular weight of Flag‑DDX3 was approximately 85 kDa) with pHA‐Ubc9 into cells resulted in a SUMOylation modification of DDX3 under action of the SUMO conjugating enzyme Ubc9. The exogenous Flag‐tagging DDX3 protein was captured from total cellular proteins using anti‐Flag antibody beads. The most obvious 100‐kDa SUMO1/2/3‐tagging DDX3 bands (S1‐DDX3, S2/3‐DDX3) appeared in co‐transfection of pFlag‐DDX3 and pHA‐Ubc9 plasmids (Figure 1E, Middle Lane), which indicated DDX3 generated SUMOylation under inducement of the SUMO conjugating enzyme Ubc9.

Conversely, the global SUMOylation degree of cellular proteins and the specific SUMOylated DDX3 level were both decreased under treatment of the SUMO inhibitor ML‐792. After incubation with 0.05–1.5 µM ML‐792 for 48 h, total SUMO‐binding proteins were decreased in a dose dependent manner (Figure S1A). Thus, we chose a half‐maximal effective concentration (EC_50_), 0.5 µM ML‐792, for subsequent cell incubation to measure the change in SUMOylated DDX3. As expected, the SUMOylated DDX3 levels, including SUMO1‐modified DDX3 (S1‐DDX3) and SUMO2/3‐modified DDX3 (S2/3‐DDX3) were greatly decreased in HepG2 cells after incubation with 0.5 µM ML‐792 for 48 h (Figure 1F).

Furthermore, we determine which specific SUMO molecule was majorly conjugated with DDX3 in HCC cells. We transiently co‐transfected plasmids pFlag‐DDX3, respectively, with pHis‐SUMO1, pHis‐SUMO2, pHis‐SUMO3, and pHA‐Ubc9 in HepG2 cells for 48 h, and the His‐SUMO‐conjugated DDX3 was enriched by IP to measure the specific SUMOylated DDX3 level in all three SUMO‐purified complexes. Our results showed DDX3 preferred to conjugate with SUMO2 in HepG2 cells (Figure 1G).

### 2.2 K81/K208/K215/K564 Residues of DDX3 are the Major SUMOylation Sites

It is necessary to clarify the SUMOylation sites of DDX3 protein for further investigating the biological function of the SUMOylated DDX3. Three common sites, including K118, K491, and K564 of DDX3 (Figure 2A), were predicted to be SUMOylated through analysis using three SUMOylation prediction tools, GPS‐SUMO, SUMOplot, and JASSA. In addition, other 6 residues, including K81, K138, K208, K215, K418, and K511, were previously reported as potential SUMOylation sites by mass spectrometry (MS)‐based proteome identification (Figure 2B).

**FIGURE 2:**
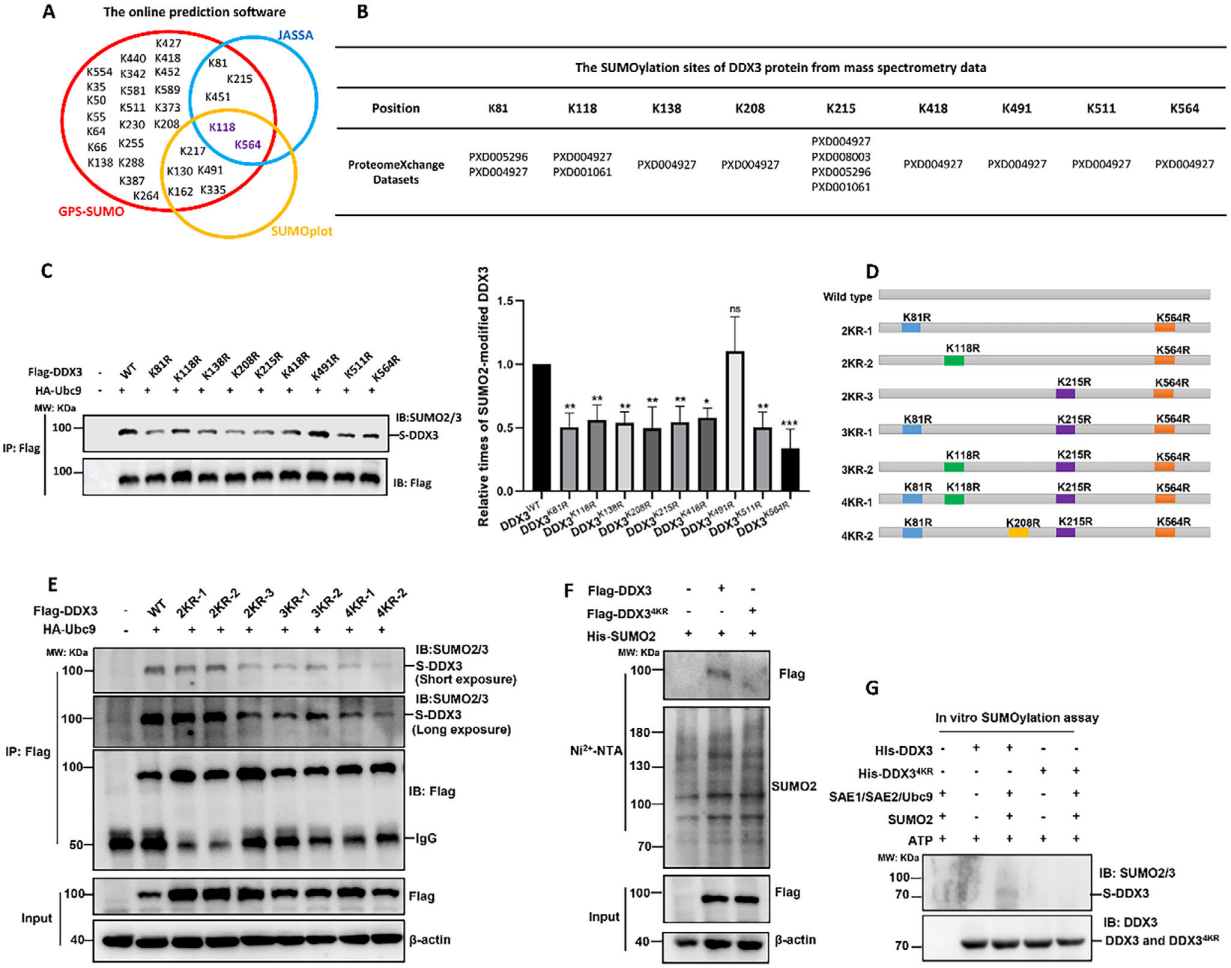
K81/K208/K215/K564 residues of DDX3 are the major SUMOylation sites conjugated with SUMO2. (A) Prediction of DDX3 SUMOylation residues using online software analysis. (B) The SUMOylation sites of DDX3 protein were summarized from ProteomXchange database with containing multiple proteomic datasets. (C) SUMOylation sites of DDX3 were confirmed by single amino acid mutation validation in HepG2 cells. The indicated K‐R mutant plasmids were transfected into HepG2 cells with pHA‐Ubc9 for 48 h, and cell lysates were subsequently immunoprecipitated using anti‐Flag antibody and immunoblotted with anti‐DDX3 and anti‐SUMO2/3 antibodies (left). When normalized with DDX3, the relative SUMOylation level of the mutant DDX3 in each group was statistically analyzed from three repeated biological experiments in a histogram (right). (D) Schematic diagram showed multiple mutations of Lys residues in full‐length DDX3, including double, triple, and quadruple mutants. (E) The SUMOylation levels of the mutant DDX3 with double or multiple K‐R mutations were decreased when compared with the wild‐type DDX3 in HepG2 cells. The indicated combination mutation plasmids were transfected into HepG2 cells for 48 h, and cell lysates were subsequently immunoprecipitated using anti‐Flag antibody and immunoblotted with anti‐DDX3 and anti‐SUMO2/3 antibodies. (F) The SUMOylation level of the mutant DDX3^4KR^ was almost disappeared when compared with the wild‐type DDX3. After transiently co‐transfection of pHis‐SUMO2 with pFlag‐DDX3 or mutant pFlag‐DDX3^4KR^ plasmids for 48 h, the His‐SUMO2‐conjugated DDX3 was pulled‐down by His tag affinity on Ni^2+^‐NTA resin, following by immunoblotting using anti‐Flag antibody. (G) In vitro SUMOylation assay of DDX3. The purified His‐DDX3 or His‐DDX3^4KR^ was incubated in tube in the presence of SAE1, SAE2, Ubc9 and SUMO2 proteins at 37°C for 1 h, then samples were analyzed with the indicated antibody. S‐DDX3: SUMO modified DDX3. DDX3^4KR^: the four residues of K81/K208/K215/K564 in DDX3 were substituted with Arg (R). IP: immunoprecipitation, IB: immunoblot, Input: same account of cell lysate to load.

According to SUMOylation prediction and previous proteome database results [23, 24], we further selected total 9 Lys sites, including the K81, K118, K138, K208, K215, K418, K491, K511, and K564, for validation by amino acid mutation. We analyzed the conservation of these K residues across species, and sequence alignment showed that all the 9 Lys residues were consistent across species (Figure S2). which suggests that these sites are functionally important. Next, we singly substituted each candidate Lys residue of DDX3 with Arg (R), and generated nine pFlag‐DDX3 mutant plasmids. Western blot analysis showed significant differences in SUMO2 modification between wild‐type DDX3 and mutants at K81, K118, K138, K208, K215, K418, and K564 sites, whereas there was no change at K491 mutation (Figure 2C). Notably, no single K mutation completely abolished DDX3 SUMOylation level, indicating potential multiple SUMO modification sites within DDX3.

We further constructed several two or multiple K residues mutation plasmids, including double, triple, and quadruple mutants to test these sites contribution to the total SUMOylation of DDX3 (Figure 2D). All mutations resulted in a marked reduction but not complete loss of DDX3 SUMOylation, whereas 4KR‐2 exhibited the lowest level of SUMOylation among all constructs tested (Figure 2E,F). We further examined whether DDX3 and its mutants could be SUMOylated in vitro. The purified His‐DDX3 or its mutant His‐DDX3^4KR^ were, respectively, incubated in the presence of the SAE1, SAE2, Ubc9, and SUMO2 proteins at 37°C for 1 h. We defined the basal SUMOylation level of DDX3 was that under incubation of DDX3 in the SUMOylation system without E3 ligase (Figure 2G, Lane 3). Notably, the DDX3^4KR^ mutant blocked the SUMOylation of DDX3 in vitro (Figure 2G, Lane 5). Taken together, DDX3 is majorly modified with SUMO2 conjugation at K81, K208, K215, and K564 sites. We defined its combinatorial mutant DDX3 as DDX3^4KR^ for the following biological function investigation.

### 2.3 DeSUMOylation Downregulates Nuclear DDX3 Level in Response to Early Stages of HBV Acute Infection

DDX3 have critical role in innate immune [18, 25], which protects the host during the early stages of virus infection. Meanwhile, virus has influences on the function of host protein and its posttranslational modification, including developing many methods to usurp SUMO conjugation to exploit the host environment for their own benefit [4, 26]. Herein, we examined whether DDX3 SUMOylation levels were influenced by HBV infection, and how SUMOylation mediated DDX3 in response to early stages of HBV infection.

Plasmids pFlag‐DDX3 and pHBV‐1.3mer were overexpressed in HepG2 or Hep3B cells to investigate DDX3 SUMOylation. DDX3 SUMOylation was reduced in the presence of HBV transfection, indicating that HBV infection downregulated the level of SUMO‐modified DDX3 (S‐DDX3) (Figure 3A). We further transfected different concentrations of pHBV‐1.3mer plasmids into HepG2 cells to compare the level of S‐DDX3. As expected, S‐DDX3 was gradually decreased dependently on concentration increase of pHBV‐1.3mer transfection (Figure S3A). Moreover, a similar result was also obtained in HepAD38 cell line (Figure 3B), which is HepG2‐derived cell line expressing HBV under tetracycline‐off control. After transfected with pFlag‐DDX3 and pHis‐SUMO2 to HepAD38 cells, the S‐DDX3 was increased when cells were induced by tetracycline (Tet+) to suppress HBV virus production (Figure 3B, Lane 2), conversely it was decreased without tetracycline action (Tet‐).

**FIGURE 3:**
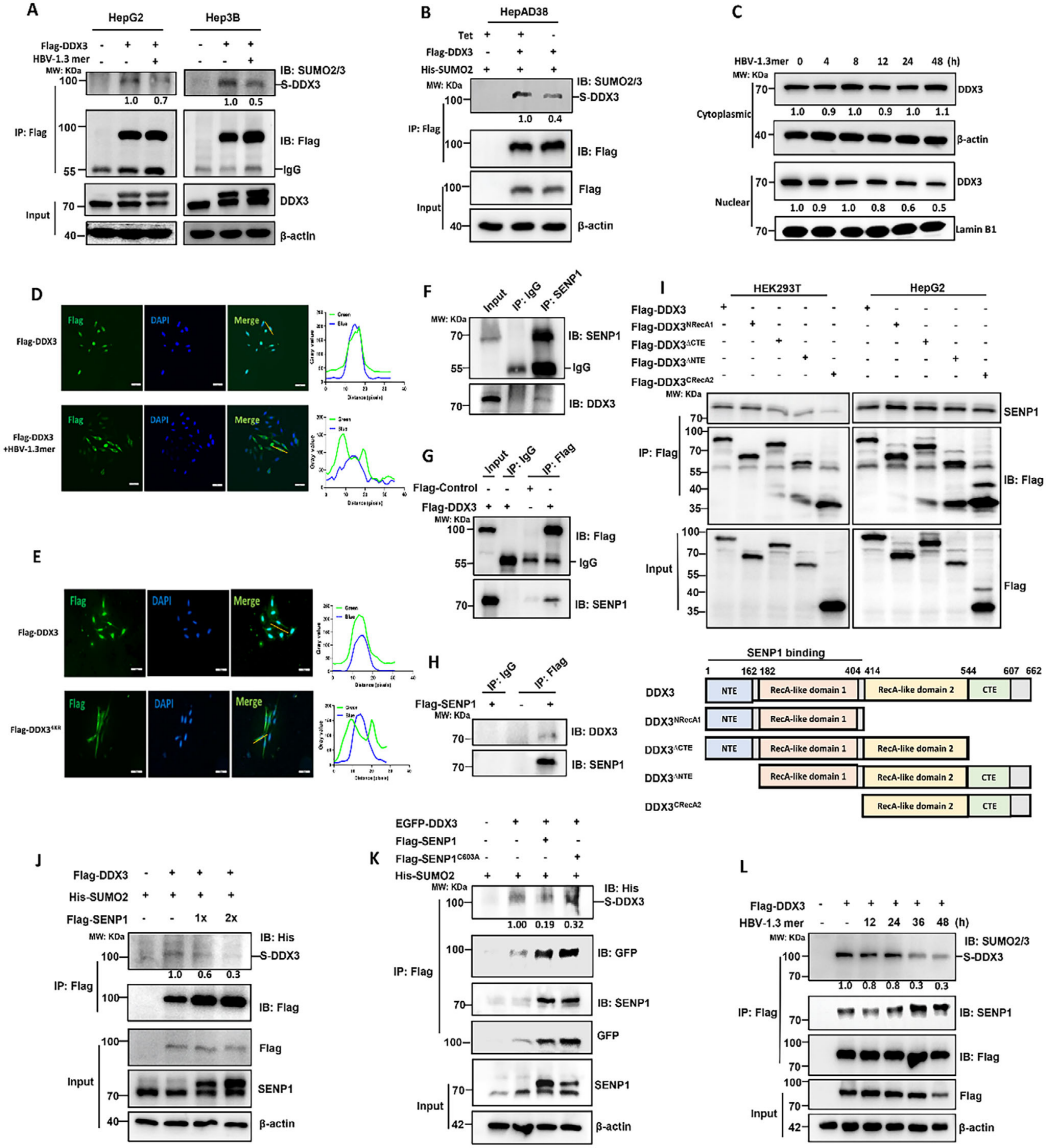
HBV induces S2‐DDX3 downregulation under SENP1‐mediated DDX3 deSUMOylation during early stages of HBV infection. (A) S‐DDX3 level was reduced in the presence of HBV. The total cell lysate was extracted from the exogenous co‐transfection of pFlag‐DDX3 and pHBV‐1.3mer into HepG2 and Hep3B cells. The Flag‐tagging DDX3 protein was enriched from cell lysate by IP on Flag antibody beads, in which the S‐DDX3 level was analyzed through Western blot using DDX3 and SUMO2/3 antibody. (B) S‐DDX3 level was reduced in the HBV‐producing cells HepAD38 (Tet‐). HepAD38 cells maintained in tetracycline can suppress virus production (Tet+). (C) Nuclear DDX3 protein was decreased within the extension of pHBV‐1.3mer transfection time from 4 to 48 h. (D) HBV‐induced nuclear DDX3 downregulation in HCC cells. The localization of DDX3 was detected in stable SUMO2‐overexpressing HepG2 cells (HepG2^His‐SUMO2^) after transfection with pHBV‐1.3mer plasmids. The yellow lines indicate the position of fluorescent intensity profiles. Scale bar, 50 µm. (E) The localization of DDX3 and mutant DDX3^4KR^ were detected in HepG2^His‐SUMO2^ cells by immunofluorescence. The yellow lines indicate the position of fluorescent intensity profiles. Scale bar, 50 µm. (F–H) The interaction between DDX3 and SENP1 at their endogenous or exogenous levels was observed with a co‐IP assay in HepG2 cells. (I) Association of SENP1 with different DDX3 truncations. HEK293T or HepG2 cells transfected with wild‐type Flag‐DDX3 or its truncations were subjected to immunoprecipitation and Western blotting with indicated antibodies. The below pattern indicated the specific truncated DDX3 fragment. (J) Upregulation of SENP1 reduced S‐DDX3 level in a dose‐dependent manner. HepG2 cells were transfected with pFlag‐DDX3, pHis‐SUMO2 and different amounts of pFlag‐SENP1, then the S‐DDX3 was analyzed by Western blotting with indicated antibodies. (K) The C603A mutation of SENP1 had no influence on its binding with DDX3, while the mutation reduced SENP1's deSUMOylase activity in catalyzing deSUMOylation of S‐DDX3. After pFlag‐DDX3 and pHis‐SUMO2 were ectopically expressed in the presence of wild‐type SENP1 or the SENP1^C603A^ mutant in HepG2 cells, the EGFP‐DDX3, Flag‐SENP1, S‐DDX3 level was respectively enriched by IP and examined by Western blotting with indicated antibodies. (L) The reduction of S‐DDX3 in response to HBV infection was associated with the interaction of SENP1 and DDX3. The expression changes of S‐DDX3, the interaction between SENP1 and DDX3 were measured in HepG2 cells after transfection of pHBV‐1.3mer plasmids at different times. S‐DDX3: SUMO modified DDX3. IP: immunoprecipitation, IB: immunoblot.

Next, we detected the level of SUMOylated DDX3 after transfection with viral analogues, Poly (I: C) and poly (dA: dT), which mimic dsRNA and dsDNA viral infection, respectively. HepG2 and Hep3B cells were treated with different concentration of poly (I: C) or poly (dA: dT) for 12 h, and the suitable concentration was screened for the follow‐up experiment based on the levels of p‐IRF3 and IFN‐β secretion (Figure S3B,D). Then, we detected the S‐DDX3 using anti‐Flag antibody after cells treatment with 1.5 µg poly (I: C) or 1 µg poly (dA: dT) for 12 h. The poly (I: C) and poly (dA: dT) can weaken the SUMO2 with DDX3 interaction and decreases the level of S‐DDX3 protein (Figure S3C,E). In general, both early HBV invasion and virus analogs attenuated DDX3 SUMOylation in host cells.

SUMOylation usually alters the cellular localization of a substrate protein, thereby affecting protein function. Consequently, we investigated whether the cellular localization of DDX3 was affected by SUMOylation. After HepG2 cells were transfected pHBV‐1.3mer plasmids, then we extracted the cytosolic and nuclear subcellular fractions of cells to detect DDX3 protein in subcellular components by Western blot. As a result, the nuclear DDX3 protein was time‐dependently decreased (Figure 3C), and this conclusion was consistent with the downregulated nuclear DDX3 when plasmids pFlag‐DDX3 with pHBV‐1.3mer were transiently transfected into SUMO2‐overexpressing HepG2 (HepG2^His‐SUMO2^) cells (Figure 3D). Moreover, pFlag‐DDX3 or pFlag‐DDX3^4KR^ was transfected into HepG2^His‐SUMO2^ cells, which demonstrated the mutant DDX3^4KR^ was mainly located in the cytoplasm when compared with DDX3 location in the nucleus (Figure 3E). Taken together, early HBV infection drives the host DDX3 de‐SUMOylation, and reduces the amount of nuclear DDX3.

### 2.4 SENP1 Catalyzes DDX3 DeSUMOylation During Early Stages of HBV Infection

SUMOylation is a reversible process catalyzed by SUMO specific protease (SENP) family protease, including SENP1, SENP2, SENP3, SENP5, SENP6, and SENP7 [15]. We identified which SENP member was involved in catalyzing the process of DDX3 de‐SUMOylation. The interaction between DDX3 and SENP at their endogenous or exogenous levels was observed with a co‐IP assay. Upon screening the SENP family, SENP1 was confirmed to interact with DDX3, which was validated by co‐IP using SENP1 or Flag antibodies (Figure 3F–H). To further map the interacting domains of DDX3 with SENP1, different truncations of DDX3 were expressed in HEK293T or HepG2 cells to measure the specific truncated sequence by co‐IP and western blotting. Although all DDX3 truncations showed a certain degree of interaction with SENP1, but variant DDX3^∆NTE^ (keeping with aa182‐662) and another variant DDX3^CRecA2^ (keeping with aa 414–662) greatly weakened their binding capacity with SENP1 (Figure 3I, Upper, Lane 4,5). The aa 1–414 of DDX3 was the main binding region with SENP1.

We co‐transfected pFlag‐DDX3, pHis‐SUMO2 with different concentrations pFlag‐SENP1 plasmids into HepG2 cells to verify SENP1‐catalyzed the de‐SUMOylation of DDX3. The increasing amounts of SENP1 expression showed a dose‐dependent decrease in S‐DDX3 (Figure 3J). The consistent results were obtained after co‐transfection pFlag‐DDX3, pHA‐Ubc9 with different concentrations pFlag‐SENP1 plasmids into HepG2 cells (Figure S3F). As known, SENP1 catalytic activity is dependent on cysteine 603 [27], we constructed a noncatalytic SENP1 variant, Flag‐SENP1^C603A^, replacing C603 with alanine (A) [28, 29], to further clarify its interaction with DDX3. After the exogenous plasmid pFlag‐SENP1 or pFlag‐SENP1^C603A^ was co‐overexpressed with pEGFP‐DDX3 (the measured molecular weight of EGFP‑DDX3 was approximately 100 KDa) and pHis‐SUMO2 plasmids in HepG2 cells, DDX3 still interacted with the catalytically inactive SENP1^C603A^ mutant (Figure 3K), which indicated their binding domain has not associated with SENP1's catalytic activity. Whereas, compared with the SENP1, SENP1^C603A^ failed to diminish S‐DDX3 in HepG2 cells (Figure 3K). Collectively, the Cys603‐containing enzyme catalytic activity domain of SENP1 mediates DDX3 de‐SUMOylation.

We further investigated the binding ability of SENP1 with DDX3 protein upon to HBV infection by transient transfection of pHBV‐1.3mer plasmids. With the transfection time of pHBV‐1.3mer extension, the interaction between DDX3 and SENP1 gradually increased, while the level of S‐DDX3 decreased (Figure 3L). Thus, the recruitment of deSUMOylase SENP1 to drive DDX3 deSUMOylation may be an emergency response mechanism for host cells during the early stage of HBV infection.

### 2.5 DDX3^4KR^ Effectively Inhibits HBV Replication In Vitro and In Vivo

We further explored DDX3 protein directly bound with HBV replication intermediate pgRNA (Figure 4A) and HBV Pol (Figure 4B), thus inhibiting HBsAg secretion (Figure 4C, Figure S4A) and promoting phosphorylation of IRF3 (p‐IRF3) (Figure 4D) to exert antiviral effect. As a member of the DEAD‐box helicase family, DDX3 suppresses viral replication and gene expression mostly dependently on its ATPase and helicase activity [30]. As known, DDX3^K230E^ has no ATPase and RNA helicase activity. While DDX3^S382L^ has its ATPase activity, but it lacks RNA helicase activity [19]. We constructed two mutants DDX3^K230E^ and DDX3^S382L^ to examine whether DDX3 ATPase activity associated with its SUMOylation. The pFlag‐DDX3^K230E^, pFlag‐DDX3^S382L^ mutant plasmids were transfected into HCC cells to analyze S‐DDX3 level. The mutation K230E and S382L had not alter DDX3 SUMOylation level, with relative expression levels of S‐DDX3 (S‐DDX3 level versus Flag level) at 0.98 and 1.05, respectively (Figure 4E). Thus, the DDX3 ATPase activity has no influence over DDX3 SUMOylation level.

**FIGURE 4:**
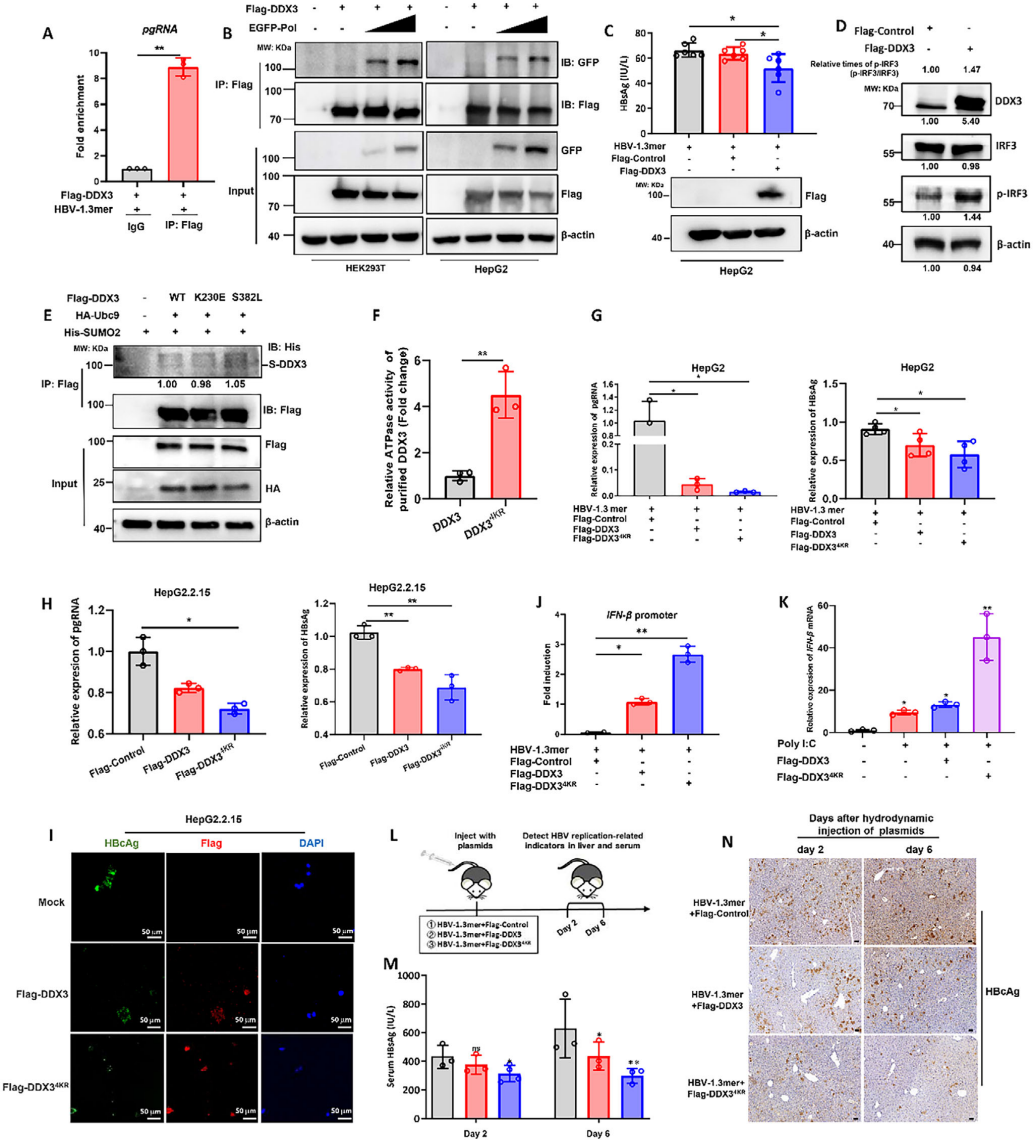
DDX3^4KR^ inhibits HBV replication in vitro and in vivo. (A) DDX3 directly bound to pgRNA by RIP assay. After plasmids pFlag‐tagged DDX3 and pHBV‐1.3mer were transfected to HepG2 cells for 48 h, cell pellets were collected to enrich Flag‐tagging proteins using anti‐Flag antibody. Immunoprecipitated pgRNA was quantitated by RT‐qPCR and normalized to the amount of immunoprecipitated proteins. (B) The dose‐dependent interaction of HBV pol with DDX3. The plasmids pFlag‐DDX3 and different concentration of pEGFP‐pol were co‐transfected into HEK293T and HepG2 cells for 48 h, cell lysates were enriched for exogenous Flag‐tagged protein, and the binding of EGFP‐pol to DDX3 was detected by Western blot. (C) DDX3 inhibited the secretion level of HBsAg in pHBV transfecting‐replication cells. (D) DDX3 overexpression enhanced the phosphorylation of IRF3 in HepG2 cells. (E) The ATPase/helicase activity of DDX3 had no influence on its SUMOylation level. DDX3 mutants (DDX3^K230E^ and DDX3^S382L^), either lacking the ATPase activity or helicase activity, were analyzed the relative change of S‐DDX3 (S‐DDX3 level vs Flag level). Briefly, the membrane was first blotted with anti‐SUMO2/3 antibody, stripped, and then re‐blotted with anti‐Flag antibody to detect exogenous DDX3. The ratios of SUMOylated DDX3 to total Flag‐DDX3 were quantified from the same membrane. (F) DDX3^4KR^ had higher ATPase activity than DDX3. The ATPase activity of DDX3 was examined using an ATPase activity kit, the OD values were normalized with background controls. (G) DDX3^4KR^ had higher antiviral activity of suppressing pgRNA expression and HBsAg secretion than DDX3 in pHBV transfecting‐replication cells. The plasmids pHBV‐1.3mer with pFlag‐DDX3, pFlag‐DDX3^4KR^ were respectively transfected into HepG2 cells for 48 h, then HBV transcription intermediate pgRNA and HBsAg secretion were detected by RT‐qPCR and Elisa. (H) DDX3^4KR^ had higher antiviral activity to suppress pgRNA expression and HBsAg secretion in stable HBV‐expressing HepG2.2.15 cells. The plasmids pFlag‐DDX3 or pFlag‐DDX3^4KR^ were transfected into HepG2.2.15 cells for 48 h, the pgRNA and HBsAg were detected by RT‐qPCR and Elisa. pFlag‐Control: as the negative control group was transfected with empty vector into HepG2.2.15 cells. (I) DDX3^4KR^ inhibited HBcAg expression in HBV‐expressing HepG2.2.15 cells. HepG2.2.15 cells were transfected with pFlag‐DDX3 or pFlag‐DDX3^4KR^ for 48 h, and HBcAg was detected by immunofluorescence. Scale bar: 50 µm. (J, K) DDX3^4KR^ had higher ability than DDX3 in enhancing IFN‐β promoter activity (J) and *IFN‐β* gene level (K) in HepG2^pHBV‐1.3mer^ cells. IFN‐β promoter was assayed through luciferase activity assay, *IFN‐β* gene level was detected by RT‐PCR. (L) A schematic diagram of the detailed groups to test DDX3 and DDX3^4KR^ effect on suppressing HBsAg in mice. 10 µg pFlag‐Control, pFlag‐DDX3 or pFlag‐DDX3^4KR^ was co‐injected with 15 µg pHBV‐1.3mer into 7‐week‐old male C57BL/6 mice by hydrodynamic injection. (M, N) The serum secretion level of HBsAg (M) and HBcAg expression in liver (N) in HBV‐infecting mice at day 2 and day 6 were respectively detected with ELISA and IHC. Scale bar: 50 µm. *p < 0.05, **p < 0.01. pFlag‐Control: hydrodynamic injection of empty vector plasmid into the tail vein of HBV‐infecting mice.

Next, we expressed DDX3 and DDX3^4KR^ in *E. coli* cells to analyze association of DDX3 SUMOylation with its ATPase activity. ATPase activity was analyzed with an ATPase activity kit as our reported approach [31]. DDX3^4KR^ had higher ATPase activity than DDX3 (Figure 4F), which demonstrates SUMOylation reduces the ATPase activity of DDX3, whereas DDX3 ATPase activity is enhanced under deSUMOylation.

We further analyzed the antiviral activity of DDX3^4KR^, a de‐SUMOylated DDX3, in HCC cells. HepG2 and Hep3B cells were transiently transfected with pHBV‐1.3mer, pFlag‐DDX3, or pFlag‐DDX3^4KR^ for 48 h. The HBV transcription intermediate pgRNA and HBsAg secretion were reduced in DDX3^4KR^ and DDX3‐expressing cells compared with the pHBV‐1.3mer transfection (Figure 4G, Figure S4B), while DDX3^4KR^ was more effectively in suppressing pgRNA and HBsAg than DDX3. Similarly, a consistent result was observed in HepG2.2.15 cells (Figure 4H). HBcAg is another marker to reflect HBV replication. The staining intensity of HBcAg in DDX3^4KR^‐ and DDX3‐overexpressing cells was lower than that in non‐transfected HepG2.2.15 cells, but the content of HBcAg in cells overexpressing DDX3^4KR^ was lower than that in the experimental group overexpressing DDX3 (Figure 4I).

Moreover, overexpression of DDX3 and mutant DDX3^4KR^ promoted the activity of the *IFN‐β* promoter in HepG2^pHBV‐1.3mer^ cells through luciferase activity assay (Figure 4J), and DDX3^4KR^ showed more effectively than DDX3 in improving *IFN‐β* promoter activity (Figure 4J) to drive *IFN‐β* gene transcription (Figure 4K). These results indicate in response to HBV infection, host cellular protein DDX3 suffers from deSUMOylation to more effectively resist to HBV.

We further established an acute HBV‐infecting mouse model with hydrodynamic injection of pHBV‐1.3mer plasmids in C57BL/6 mice to explore DDX3^4KR^ influence on HBV replication indicators in vivo. Mouse serum and liver tissues were collected at 0, 1, 2, 6, 8, and 10 days after pHBV‐1.3mer injection. HBcAg in liver and serum HBsAg were highest on day 6, but became lower since day 8 (Figure S4C–F). In addition, we co‐injected with pFlag‐DDX3 plasmids with the pHBV‐1.3mer into mouse, and the expression level of HBcAg in DDX3‐expressing mouse liver at day 2 and 6 was greatly decreased when compared to the mock control (Figure S4G,H), which demonstrated DDX3 inhibited HBcAg expression and secretion in the liver.

Furthermore, we detected DDX3^4KR^ influence on HBcAg expression and secretion in HBV‐infecting mouse on day 2 and 6 (Figure 4L). Compared with the of HBV replication mouse model, HBsAg in mouse serum (Figure 4M) and liver (Figure 4N) was both decreased in DDX3‐expressing and DDX3^4KR^‐expressing group. Especially, DDX3^4KR^ more efficiently inhibited HBsAg secretion in serum and expression in mouse liver.

### 2.6 SENP1 Downregulation and HBV Pol Accumulation Cooperate to Enhance DDX3 SUMOylation in HBV‐HCC

Based on SENP1‐mediated deSUMOylation of DDX3, we further investigated whether SENP1 expression was altered in the transformation for chronic HBV to HBV‐HCC. We extracted the total RNA and total protein of HepG2 and HBV stable replication cells (HepG2.2.15, HepAD38), and detected the endogenous expression levels of mRNA and protein of SENP1. SENP1 expression was decreased in the HBV persistent replication HepG2.2.15 and HepAD38 cells, compared with HepG2 cells (Figure 5A,B). And the levels of the S‐DDX3 were elevated in HBV‐replicating HepG2.2.15 cells (Figure 5C). At the same time, we collected six cases of HBV‐positive (n = 3) and HBV‐negative (n = 3) liver tissues, which were obtained from the non‐cancerous regions of HCC patients during surgical resection, to investigate the effect of HBV infection on SENP1 expression by immunofluorescence analysis. Compared with HBV‐negative liver tissues, SENP1 was downregulated in HBV‐positive liver tissues, and the level of S‐DDX3 was also reduced (Figure 5D,E).

**FIGURE 5:**
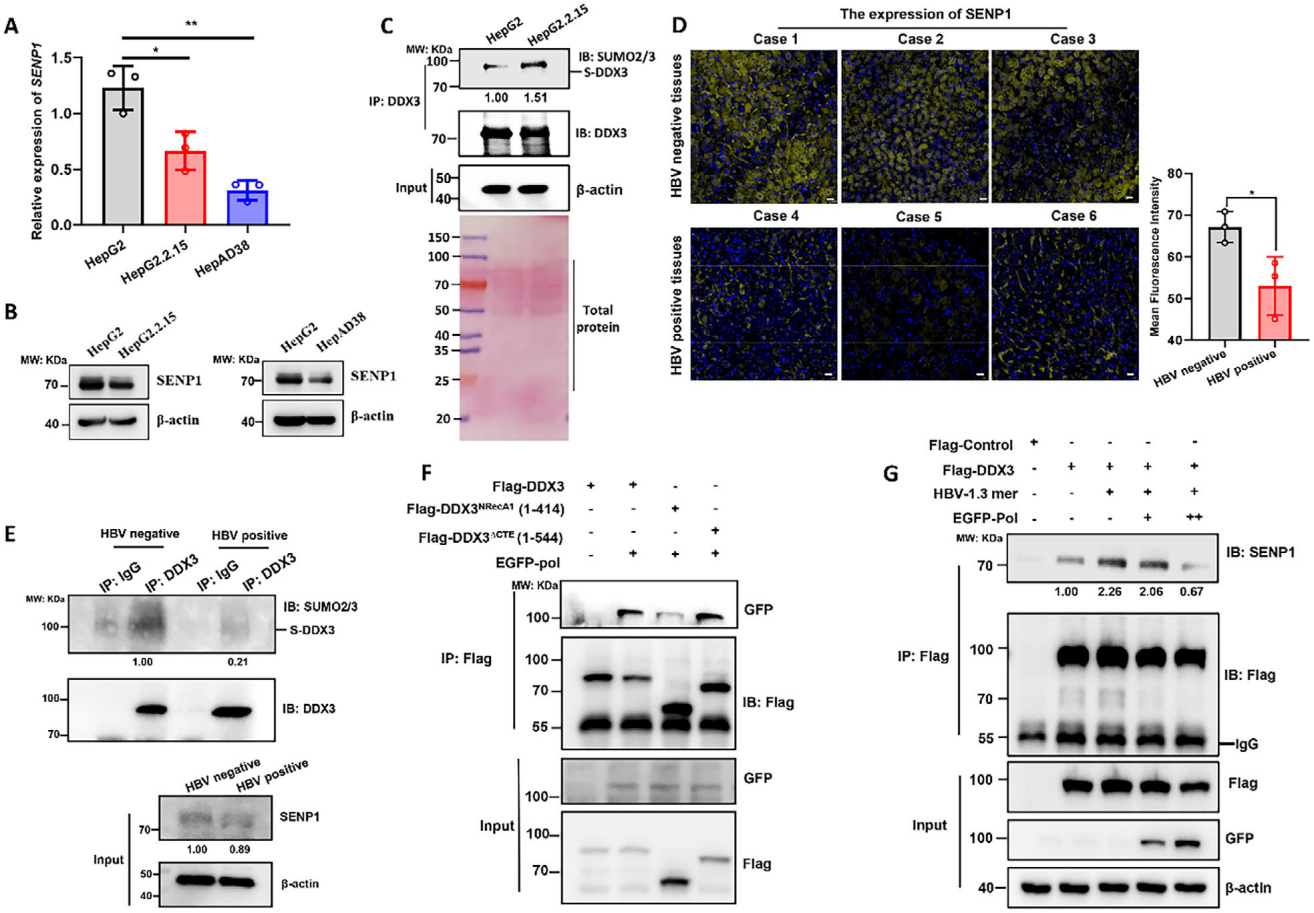
SENP1 downregulation and HBV Pol accumulation cooperate to promote DDX3 SUMOylation in HBV‐HCC. (A, B) SENP1 is decreased in the HBV persistent replication HepG2.2.15 and q12weHepAD38 cells. Extracted the total RNA and total protein of HepG2 and HBV stable replication cells (HepG2.2.15, HepAD38), and detected the expression levels of SENP1 mRNA and protein by RT‐qPCR and Western blot, respectively. (C) The levels of DDX3 SUMOylation were increased in HepG2.2.15 cells. Cellular endogenous DDX3 were enriched by IP to detect SUMOylated DDX3 against anti‐SUMO2/3 antibody. Ponceau S staining of the membrane and immunoblotting for β‐actin as a total protein loading control. (D, E) Expression levels of SENP1, DDX3 and S‐DDX3 in three HBV‐negative and three HBV‐positive human liver tissues were detected by immunofluorescence staining (D) and Western blot (E), respectively. Scale bar: 20 µm. *p < 0.05. IP and immunoblotting analysis with indicated antibodies in HBV‐negative/positive liver tissues. As a negative control, the primary antibody was replaced with the normal IgG to eliminate the nonspecific protein binding. The SUMOylated DDX3 (S‐DDX3) was determined and normalized to DDX3 protein. (F) HBV pol bound to DDX3^∆CTE^ variant. The plasmids pEGFP‐pol, pFlag‐DDX3, pFlag‐DDX3^NRecA1^ and pFlag‐DDX3^∆CTE^ were co‐transfected into HepG2 cells for 48 h, cell lysates were enriched for exogenous Flag‐tagged protein, and the binding of EGFP‐pol to DDX3 was detected by Western blot. (G) HBV Pol inhibited the interaction between DDX3 and SENP1. HepG2 cells were transfected with plasmids pFlag‐DDX3, pHBV‐1.3mer and increasing amounts of the plasmids pEGFP‐Pol. After 48 h, the cells were collected and extracted for Co‐IP analysis, the levels of Flag and SENP1 were analyzed through Western blot. S‐DDX3: SUMO modified DDX3. IP: immunoprecipitation, IB: immunoblot.

On the other side, with the persistent replication of HBV in host cells, a large number of HBV‐encoding proteins produce and accumulate in host cells, including HBV Pol, which affects host cell proteins functions. We measured if HBV Pol accumulation disrupted the interaction between DDX3 and SENP1. We co‐transfected plasmids pEGFP‐pol with pFlag‐DDX3, or each variant pFlag‐DDX3^NRecA1^ and pFlag‐DDX3^∆CTE^ into HepG2 cells for 48 h, then cell lysates were extracted to enrich the exogenously expressed Flag‐tagging proteins. DDX3 interacted with HBV Pol (Figure 5F, Lane 2) as reported research [21]. Moreover, both DDX3^NRecA1^ and DDX3^∆CTE^ variants bound with HBV Pol (Figure 5F, Lane 3–4), indicating that aa 1–414 of DDX3 was the main binding region with HBV Pol.

Furthermore, we co‐transfected plasmids pEGFP‐Pol, pFlag‐DDX3, and pHBV‐1.3mer in HepG2 cells to confirm HBV Pol accumulation hampered the interaction of DDX3‐SENP1. The binding of DDX3 and SENP1 became weaker with increasing HBV Pol concentration (Figure 5G, lanes 4 vs 5), which supported HBV Pol accumulation disrupted the interaction between DDX3 and SENP1. Taken together, SENP1 downregulation and HBV Pol accumulation result in upregulation of DDX3 SUMOylation in HBV‐HCC.

### 2.7 DDX3 SUMOylation Favors *KLF4α* Generation by Exon Skipping of *KLF4* mRNA to Promote HBV‐HCC Cell Cycle Progression

Due to RNA helicase involving in the transcription and translation levels of cell cycle regulators [32, 33], we investigated roles of DDX3 and its SUMOylation in cancer cell cycle progression. As a result, DDX3 overexpression blocked up cell cycle at G1 phase of HBV‐HCC cells (Figure S5A) by reducing expression of cyclin D1 (Figure 6A), and inhibited tumor growth in nude mice xenografted with HCC cells (Figure 6B). Our data were consistent with the previous reports that DDX3 upregulation inhibited cell proliferation and DDX3 knockdown promoted cyclin D1 and cycle progression in HCC [34].

**FIGURE 6:**
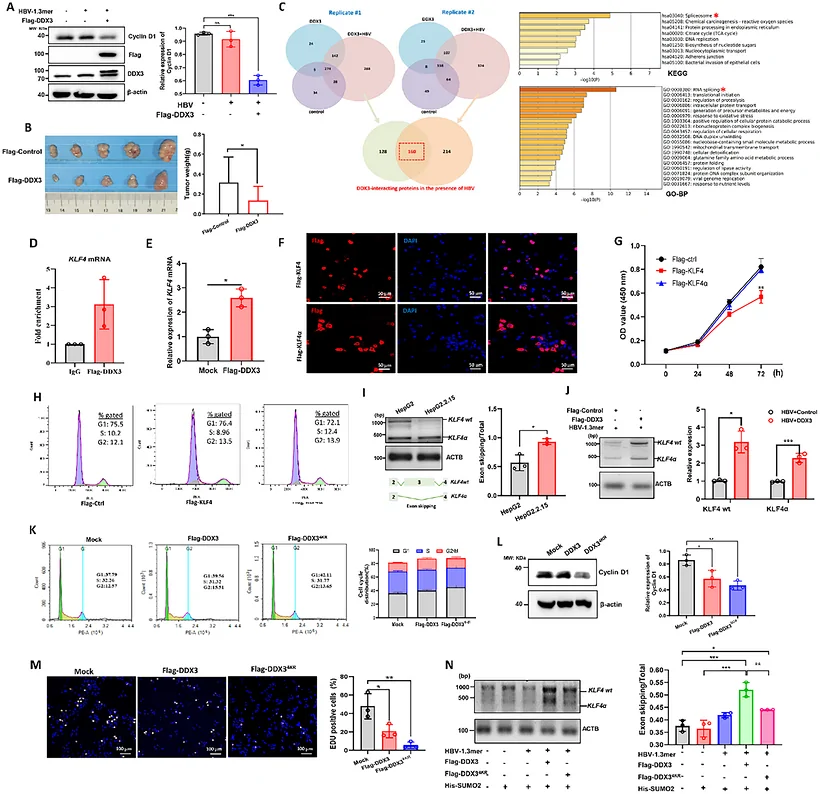
DDX3 SUMOylation favors exon skipping of *KLF*4 mRNA to promote HBV‐HCC cell cycle progression. (A) DDX3 downregulated Cyclin D1 expression in HBV‐HCC cells. The plasmids pHBV‐1.3mer and pFlag‐DDX3 were transfected to HepG2 cells for 48 h, then detected Cyclin D1 expression by western blot. (B) DDX3 reduced tumor growth ability of the xenograft nude mice. Mice were killed after cell injection for 32 days (tumor growth for 20 days), and tumors were isolated from xenograft nude mice. The average tumor weight of pFlag‐DDX3‐overexpressing mice was lighter than the control of pFlag vector group. Flag‐DDX3: nude mice inoculated pFlag‐DDX3‐overexpressing HepG2 cells; Flag‐Control: nude mice inoculated pFlag‐transfected HepG2 cells. *p < 0.05. (C) DDX3 protein participated in RNA splicing process in HBV‐HCC cells. The plasmids pFlag‐DDX3 with or without pHBV‐1.3mer were transfected into HepG2 cells for 48 h, while transfection of the empty vector pFlag was taken as the negative control. Then the cell lysate was immunoprecipitated with anti‐Flag magnetic beads, and Flag‐tagging DDX3 complex was detected by MS/MS. control: The group was transiently transfected with the empty vector pFlag into HepG2 cells. DDX3: The group was transiently transfected with plasmids pFlag‐DDX3 into HepG2 cells. DDX3+HBV: The group was transiently co‐transfected plasmids pFlag‐DDX3 with pHBV‐1.3mer into HepG2 cells. (D) DDX3 binds *KLF4* mRNA. IP assay was performed in HepG2 cell lysates transfecting pFlag‐tagged DDX3 plasmids. Immunoprecipitated *KLF4* mRNA was quantitated by RT‐qPCR and normalized to the amount of immunoprecipated proteins. (E) The expression of *KLF4* mRNA in HCC cells transfected with pFlag‐DDX3 plasmids. (F–H) HepG2.2.15 cells were transiently transfected with pFlag‐KLF4 or pFlag‐KLF4α for 48 h, then we detected the expression distribution, cell cycle distribution and cell proliferation by immunofluorescence, CCK8 and flow cytometry, respectively. (I) Identification of *KLF4* isoforms in HBV‐HCC. The total RNA as a template for RT‐PCR analysis of *KLF4* isoforms using the forward and reverse primers that cover both the 5’‐ and 3’‐UTR regions of *KLF4* transcript. (J) DDX3 improved the expression of *KLF4wt* and isoform *KLF4α*. (K‐M) Compared to DDX3^4KR^, DDX3 more efficiently arrested cell cycle in G1/S phase (Figure 6K) by reducing Cyclin D1 expression (Figure 6L) and inhibited cell proliferation (Figure 6M). The plasmids pFlag‐DDX3 and pFlag‐DDX3^4KR^ were transfected in the stable overexpressing SUMO2 cells, and the cells were detected cell cycle by flow cytometry (K). Representative images of the EdU assay of HepG2.2.15 cells transfected with pFlag‐DDX3 or pFlag‐DDX3^4KR^ (M). EdU‐positive cells represent proliferating cells. (N) The expression of *KLF4wt* and isoform *KLF4α* in HepG2.2.15 cells overexpressing DDX3^WT^ or DDX3^4KR^. *p < 0.05, **p < 0.01, ***p < 0.001.

In addition, we detected DDX3‐mediated biological processes during HBV replication through identification of DDX3‐binding proteins by MS/MS. Plasmids pFlag‐DDX3 with pHBV‐1.3mer were co‐transfected into HepG2 cells for 48 h, then DDX3‐binding proteins were enriched against Flag antibody from cell lysate and identified by MS/MS. We screened 160 DDX3‐interacting proteins in the presence of HBV by integrating twice IP‐MS datasets (Figure 6C, Left). The KEGG pathway and GO‐BP enrichment analysis of these interactors indicated DDX3 with its interacting proteins involved in spliceosome and RNA splicing (Figure 6C, Right).

DDX3 belongs to an RNA‐binding protein, and closely associates with mRNA binding and RNA splicing. We transfected the plasmid pFlag‐DDX3 to HepG2 cells for 48 h, then preformed RNA immunoprecipitation sequencing (RIP‐seq), which confirmed DDX3 bound to multiple mRNAs (|log2FC|>1, p < 0.05) (Figure S5B), within the 5’UTR, 3’UTR, and CDS regions of target genes (Figure S5C). Moreover, these mRNAs were enriched in RNA metabolism‐related pathways, including spliceosome, RNA transport, RNA degradation, and ribosome biosynthesis (Figure S5D). These data support that DDX3 functions as an RNA‐binding protein to directly bind mRNAs and plays an important function in RNA metabolism.

We further detected whether SUMOylation regulated biological processes of DDX3. The plasmids pFlag‐DDX3 with pHis‐SUMO2 were transfected into HepG2 cells for 48 h, while transfection of the empty vector pFlag was used as the negative control. DDX3‐binding SUMO‐relative proteins were immunoprecipitated against Flag antibody from cell lysate and identified by MS/MS. Finally, a total of 55 DDX3‐interacting partners were identified in the context of SUMO2 overexpression (Figure S5E). The details of MS data were summarized in Table S1. The KEGG pathway enrichment analysis of these interactors was performed by Metascape (http://metascape.org/) [35]. And top 20 of KEGG pathway annotation were shown in Figure S5E. Under SUMO2 overexpression, the interaction partners of DDX3 involved in cell cycle and viral oncogenesis (Figure S5E). Meanwhile, the flow cytometry results also confirmed that overexpression of SUMO2 promoted cell cycle (Figure S5F).

Combined with MS and RIP‐seq data, one cell cycle‐related transcription factor *KLF4* was focused to investigate its association with DDX3. RIP experiment showed that the binding factor of DDX3 and *KLF4* mRNA was 2.1 times, which was significantly higher than DDX3 binding with *KLF17* mRNA and *KLF13* mRNA (Figure 6D, Figure S5G). In addition, DDX3 positively regulated KLF4 expression (Figure 6E, Figure S5H).

Except to the encoded wild‐type KLF4 (KLF4 WT), *KLF4* mRNA has five alternative splice variants, respectively encoding KLF4α (KLF4‐1), KLF4‐2. KLF4‐3 KLF4‐4, and KLF4‐5 through TCGA‐LIHC dataset assays from TSVdb database (http://www.tsvdb.com/index.html) (Figure S5I). RNA‐seq results showed that DDX3 knockdown resulted in *KLF4* mRNA skipping at exon 3, which produced a spliced isoform *KLF4α* (Figure S5J). As known that *KLF4* splice variants antagonize wild‐type *KLF4* mRNA (*KLF4wt*), and *KLF4* variants retards the *KLF4wt*‐mediated inhibition of Cyclin D1 expression [36].

Herein, we first analyzed the role of *KLF4wt* and the spliced isoform *KLF4α* in HBV‐HCC cells. Immunofluorescent staining showed that Flag‐tagged KLF4wt remained in the nucleus and Flag‐tagged KLF4α located in the cytoplasm (Figure 6F). The same results were obtained after transfection with the EGFP‐tagged plasmid in HepG2.2.15 cells (Figure S5K). Compared to KLF4wt, overexpression of KLF4α significantly promoted HepG2.2.15 cell cycle progression and proliferation (Figure 6G,H). Taken together, KLF4α and KLF4 have opposite functions in HCC cell cycle and proliferation. KLF4α attenuates KLF4 WT role of suppressing HCC cell proliferation.

We further analyzed the expression levels of *KLF4wt* and the spliced isoform *KLF4α* in the HBV replication cells. The total RNAs were a template for RT‐PCR analysis of *KLF4* isoforms using the forward and reverse primers that covered both the 5’‐ and 3’‐UTR regions of *KLF4* transcript. Compared with HepG2 cells, the expression of *KLF4wt* was lower in HepG2.2.15, but the expression of *KLF4α* was not altered (Figure 6I). DDX3 positively regulated *KLF4* mRNA expression, which increased the expression of *KLF4wt* and isoform *KLF4α* (Figure 6J). Then, we detected the influence of DDX3 and its SUMO mutant DDX3^4KR^ on HBV‐HCC cell proliferation, including Cyclin D1 expression and cell cycle distribution. Compared to DDX3^4KR^, DDX3 more efficiently arrested cell cycle in G1/S phase (Figure 6K) by reducing Cyclin D1 expression (Figure 6L) and inhibited cell proliferation (Figure 6M). Overexpression of DDX3 or DDX3^4KR^ changed the transcript of *KLF4* mRNA. However, DDX3^4KR^ caused the splice variant quantity of *KLF4α* less than that of DDX3 (Figure 6N). These results suggest that DDX3 SUMOylation promotes *KLF4* mRNA to skip in exon 3 to generate more *KLF4α* isoforms, which enhances Cyclin D1 expression and contributes to HBV‐HCC cell proliferation.

### 2.8 BRCC Domain of TRIM28 Binds With the N‐Terminal DDX3 to Enhance S2‐DDX3 in HBV‐HCC Cells

We further investigated if the SUMO E3 ligase regulated the SUMOylation of DDX3. We constructed HepG2 cells stably expressing His‐tagging SUMO2 by lentiviral infection, which were defined HepG2^His‐SUMO2^ cells, and then we employed immunoprecipitation‐mass spectrometry (IP‐MS) to identify DDX3‐interacting proteins in Flag‐tagging DDX3 overexpressing HepG2^His‐SUMO2^ cells. Cell pellets were lysed to enrich DDX3‐binding proteins by anti‐Flag magnetic beads, and the concentrated proteins were identified by MS (Figure 7A). By integrating triple IP‐MS datasets, 423 DDX3‐interacting proteins were identified (Figure 7B, Table S2). Interestingly, the TRIM28 [37, 38, 39], a SUMO E3 ligase, was one of the DDX3‐interacting proteins (Figure 7B). The characteristic peptides of the target protein DDX3 and the interacting protein TRIM28 were shown in Figure 7C.

**FIGURE 7:**
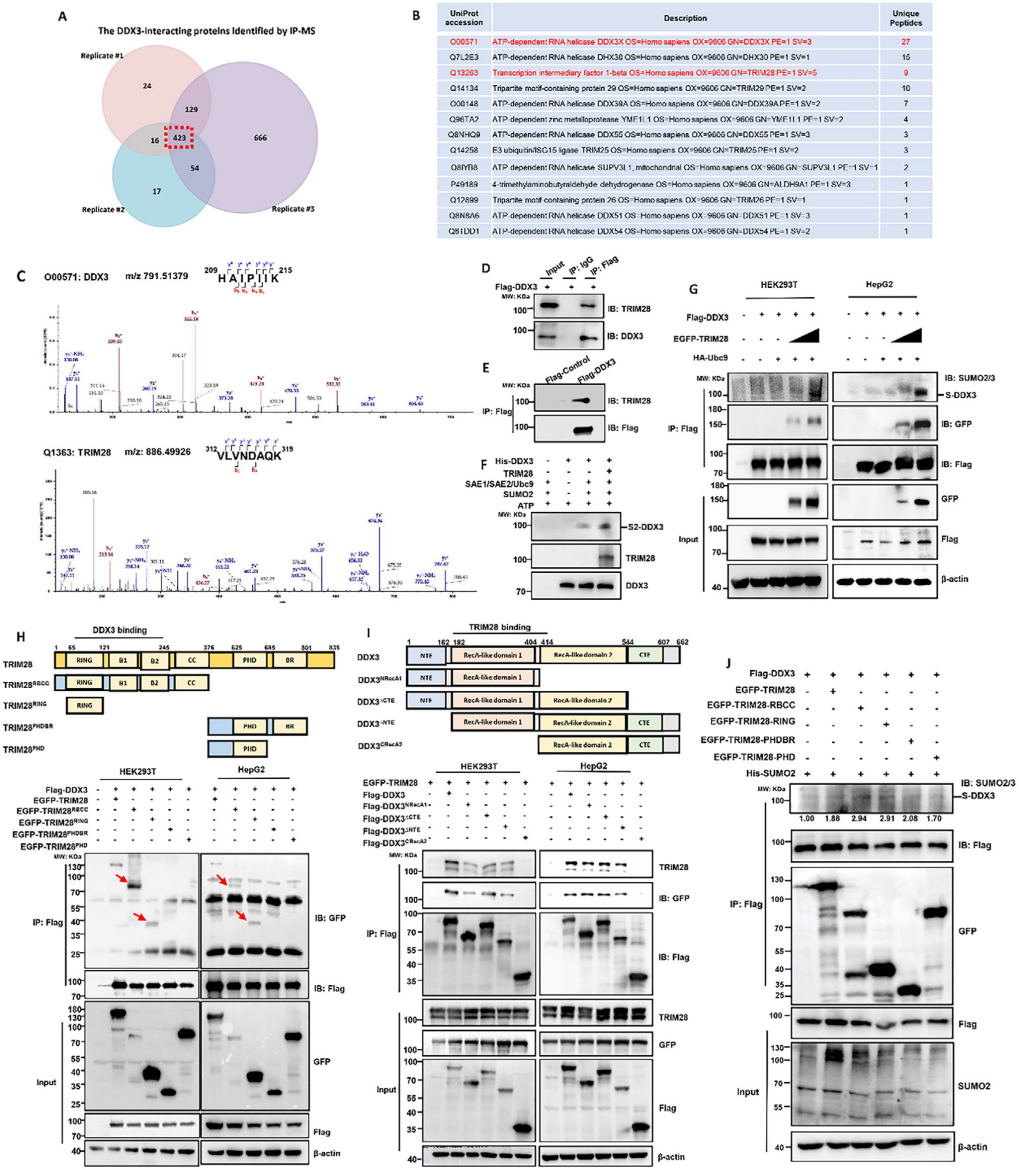
TRIM28 enhances DDX3 SUMOylation levels. (A) MS/MS identification profiling of DDX3‐interacting proteins with three biological replicates. The plasmid pFlag‐DDX3 was transfected into HepG2 cells stably expressing His‐tagging SUMO2 (HepG2^His‐SUMO2^) to overexpress Flag‐tagging DDX3, followed by IP‐MS identification of DDX3‐interacting proteins. Different colors represented the identification number of DDX3‐interacting proteins by each IP‐MS, and the common 423 partners were identified by three IP‐MS datasets. (B) TRIM28 was identified as a DDX3‐interacting protein among the top 12 DDX3‐interacting proteins from IP‐MS. (C) The matched peptide information for DDX3 and TRIM28 in MS/MS. The red b ion indicates the ion fragment from N‐terminal to C‐terminal of each peptide. The subscript number and bottom digits of b ion labelling the peak represents the number of N‐terminal amino acids and their total molecular weight. Similarly, the blue y ion indicates the ion fragment from C‐terminal to N‐terminal. The subscript number and bottom digits of y ion present the number of C‐terminal amino acids and their total molecular weight. The sequences of each peptide are calculated based on the b ions and y ions and their molecular weights. The letters on the top of each figure mean the single letter codes for the amino acids identified by MS. (D, E) IP and western blot analyses were performed to detect the interaction of endogenous TRIM28 with exogenous DDX3 in HepG2 cells. (F) In vitro SUMOylation assay confirmed TRIM28 catalyzes and promotes DDX3 SUMOylation. The purified His‐tagging DDX3 protein and His‐tagging TRIM28 were subjected to test DDX3 SUMOylation in vitro, and levels of DDX3 SUMOylation were determined by western blot using anti‐SUMO2/3 antibody. (G) TRIM28 promoted the SUMOylation levels of DDX3 in cells. HEK293T or HepG2 cells were transiently transfected with the increase amounts of pEGFP‐TRIM28 plasmids, followed by IP and WB analyses. (H) The RBCC domain of TRIM28 was necessary to interact with DDX3. Schematic of wild‐type TRIM28 and four TRIM28 truncations (Upper). Association of DDX3 with the RING finger domain, two B box domains, and coiled‐coil domain of TRIM28 (Below). Plasmid pFlag‐DDX3 was co‐transfected with plasmids encoding full‐length TRIM28 or its truncations into HEK293T or HepG2 cells for 48 h, and cell lysates were immunoprecipitated with anti‐Flag affinity beads to enrich DDX3 binding complexes, which were analyzed by western blot with the indicated antibodies. (I) DDX3 relied on the NRecA1 (aa 1–414) domain to interact with TRIM28. Schematic of wild‐type DDX3 and four DDX3 truncations (Upper). Association of TRIM28 with the NRecA1(aa 1–414) of DDX3 (Below). HEK293T or HepG2 cells transfected with EGFP‐TRIM28 and wild‐type Flag‐DDX3 or its truncations were subjected to perform IP and western blotting with indicated antibodies. (J) RBCC domain of TRIM28 had a key role in binding and modifying DDX3. HepG2 cells were transfected with plasmids pFlag‐DDX3, pEGFP‐TRIM28 and its truncations for 48 h. Then cell pellets were collected to extract cellular lysates to enrich Flag‐tag DDX3 protein by IP, from which the S‐DDX3 was detected through western blot. S‐DDX3: SUMO modified DDX3.

We overexpressed DDX3 in HepG2 cells and validated that DDX3 was co‐pulled down with TRIM28 (Figure 7D,E), which was consistent with the IP‐MS identification. In addition, the global SUMO2‐modification level was increased under TRIM28 overexpression (Figure S6A). To further confirm TRIM28 catalyzes and promotes DDX3 SUMOylation, we respectively purified His‐tagging DDX3 protein and His‐tagging TRIM28 from *E.coli* cells referencing our reports [31] to test DDX3 SUMOylation in vitro. When incubated without the DDX3 protein in the SUMOylation system, no SUMOylated DDX3 band was observed (Figure 7F, Lane 1). In the SUMOylation reactions containing the DDX3 protein, SUMOylated DDX3 at the expected molecular weight was detected (Figure 7F, Lane 3). Upon incubation with the SUMO E3‐ligase TRIM28, the band of S‐DDX3 became stronger (Figure 7F, Lane 4) compared with the TRIM28‐free conditions (Figure 7F, Lane 3), indicating that TRIM28 catalyzes and promotes the SUMOylation levels of DDX3 by conjugation with SUMO2. Moreover, the plasmids pFlag‐DDX3 with different concentrations of pMyc‐TRIM28 or pEGFP‐TRIM28 were co‐transfected into HepG2 or HEK293T cells to detect the SUMOylated DDX3 changes. With the increase of TRIM28 expression, the interaction of TRIM28 with DDX3 was increased, and the level of SUMOylated DDX3 was also enhanced (Figure 7G, Figure S6B). Together, TRIM28 promoted DDX3 SUMOylation in HCC cells.

To map the specific interacting domains of TRIM28 to bind DDX3, several truncated variants of both TRIM28 and DDX3 were co‐expressed in HEK293T or HepG2 cells, then the binding protein complex was enriched by co‐IP and detected by western blotting. A series of TRIM28 truncation mutants revealed that the RBCC (aa 1–376) and RING (aa 65–121) domains of TRIM28 bound to the DDX3, whereas PHDBR (aa 376–801) and PHD (aa 376–685) of TRIM28 did not to bind with DDX3, suggesting that the RING domain itself and the region encompassing the B box and CC domain of TRIM28 is necessary to bind with DDX3 (Figure 7H). And TRIM28 improved DDX3 SUMOylation, which was dependent on its RBCC domain (Figure 7I). Similarly, we constructed several truncated DDX3 plasmids based on the conserved domains of DDX3, and validated the fragment of DDX3 with aa 1–414 interacted with TRIM28 (Figure 7J). In conclusion, the BRCC domain of TRIM28 binds N‐terminal DDX3 to enhance the SUMOylated DDX3 levels in HBV‐HCC cells.

### 2.9 DDX3 is Positively Correlated With KLF4 in HCC, and High DDX3 With Low TRIM28 Expression Means a Better Overall Survival for HBV‐HCC Patients

DDX3 represents an intrinsic host antiviral factor that restricts HBV transcription [18], we further analyzed the correlation between DDX3 and *KLF4*, TRIM28 expression in clinical HBV‐HCC tissues. Bioinformatics data of GSE121248, GSE55092, and GSE62232 from the Gene Expression Omnibus database (GEO) revealed *DDX3* mRNA was decreased in HBV‐HCC tissues compared with adjacent tissues (Figure S7A).

We detected the expression level of DDX3 protein in the HCC tissue microarray, which including 90 HCTs and 90 PLTs (Figure 8A). The typical IHC images of DDX3 expression with different pathologic grade were displayed in Figure 8A. Lower DDX3 expression was in HCC tissues with averagely scoring 6.71 ± 2.99 compared with 90 counterparts (*p* < 0.01) (Table 1, Figure 8B left). Moreover, according to the HCC patient's information, 90 human HCC patients were classified into two groups, HBsAg‐negative (n = 21) and HBsAg‐positive (n = 48). A significant downregulation of DDX3 expression was also found in HCC from HBsAg‐positive patients with averagely scoring 6.67 ± 2.98 compared with 77 counterparts (*p* < 0.01) (Table 1, Figure 8B right). We further analyzed the possible relationship between DDX3 expression in HCC tissues and pathological grading. We performed a linear regression analysis of the histopathological grade and DDX3 immunohistochemical score in 90 patients. In Figure 8C, DDX3 expression in 90 HCC patients was correlated with the tumor pathological grade (correlation coefficient r^2^ = 0.09138, *p* < 0.01). Except the HBsAg‐negative patient, DDX3 expression was also correlated with the pathological grade of 77 HBsAg‐positive HCC patients (correlation coefficient r^2^ = 0.09365, *p* < 0.01).

**FIGURE 8:**
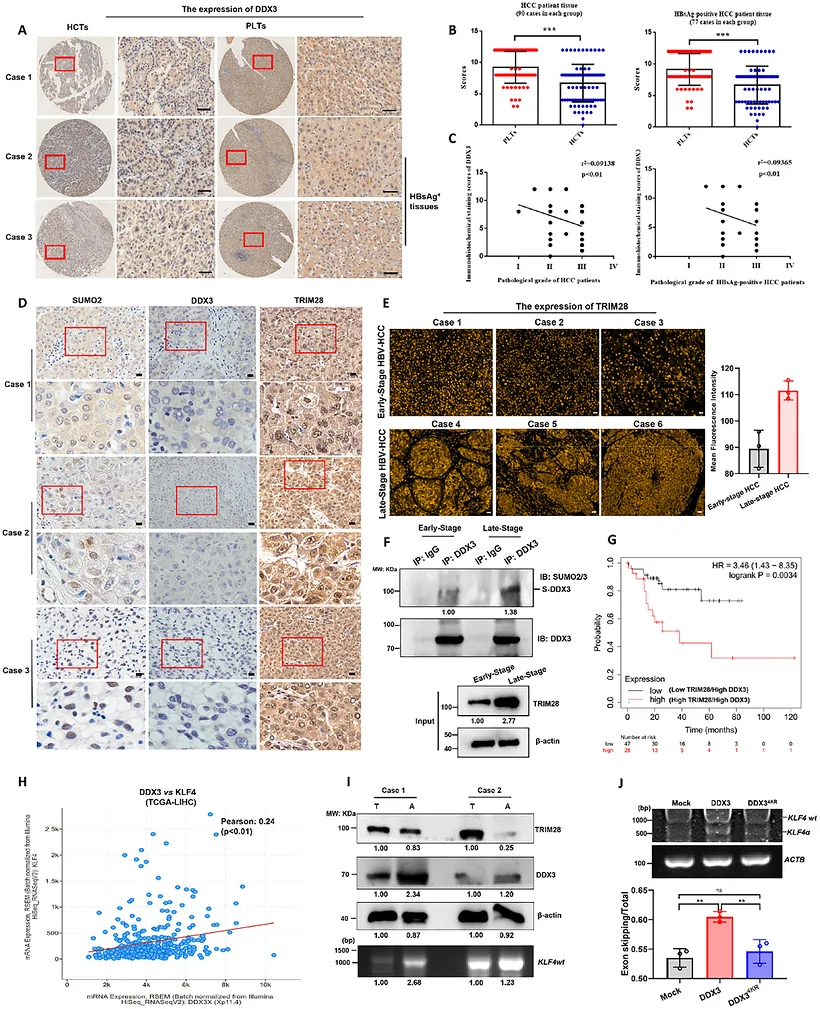
Correlation among DDX3, TRIM28 and *KLF4* expression in HBV‐HCC tissues. (A) DDX3 expression in human PLTs and HCTs was detected by IHC analysis. The typical images IHC staining of DDX3 in three cases of HCC patients. (B) Histogram of immunohistochemical staining scores of DDX3 in HCC patients. (C) A linear regression analysis of the histopathological grade and DDX3 immunohistochemical score in HCC patients. (D) The expression and localization of DDX3, SUMO2 and TRIM28 in PLTs and HCTs were detected by IHC analysis. HCTs: HCC tissues, PLTs: patients’ autologous para‐cancerous liver tissues. (E‐F) Expression levels of TRIM28 and S‐DDX3 in early/advanced‐stage HBV‐HCC tissues were detected by immunofluorescence staining (E) and Western blot (F). Scale bar: 20 µm. *p < 0.05. IP and immunoblotting analysis with indicated antibodies in early/advanced‐stage tumor tissues resected from HBV‐HCC patients. As a negative control, the primary antibody was replaced with the normal IgG to eliminate the nonspecific protein binding. (G) HBV‐HCC patients with low TRIM28 and high DDX3 expression have a better prognosis using Kaplan–Meier plotter online tools. (H) Gene expression correlation between *DDX3* and *KLF4* in HCC using cBioPortal online database. (I) The expression of DDX3 and TRIM28 in HCC tissues and adjacent tissues were detected by IHC analysis. And the expression of *KLF4wt* in HCC tissues and adjacent tissues were detected by RT‐qPCR. (J) The expression of *KLF4wt* and isoform *KLF4α* in PBMC cells overexpressing DDX3^WT^ or DDX3^4KR^. The PBMC cells were derived from the peripheral blood of HBV‐HCC patients.

**TABLE 1:** DDX3 expression profiling in HCC and patients’ autologous para‐cancerous liver tissues.

DDX3 expression in 90 HCTs and 90 PLTs					
Immuno‐reactivity	HCTs (n = 90)		PLTs (n = 90)		*p* value
	Percentage	Average score	Percentage	Average score	
Total	100% (90/90)	6.71 ± 2.99	100%(90/90)	9.23 ± 2.51	*p* < 0.01
Low (+)	33.3% (30/90)	3.20 ± 1.05	5.6% (5/90)	3.60 ± 0.49	
High (++)	66.7% (60/90)	8.47 ± 1.89	94.4% (85/90)	9.56 ± 2.17	

DDX3 expression in 77 HBsAg‐positive HCTs and 77 PLTs (Exclude HBV‐negative patients)					
Immuno‐reactivity	HCTs (n = 77)		PLTs (n = 77)		*p* value
	Percentage	Average score	Percentage	Average score	
Total	100% (77/77)	6.67 ± 2.98	100%(77/77)	9.13 ± 2.49	*p* < 0.01
Low (+)	33.8% (26/77)	3.19 ± 1.07	5.2% (4/77)	3.50 ± 0.5	
High (++)	66.2% (51/77)	8.43 ± 1.89	94.8% (73/77)	9.43 ± 2.17	

HCTs: HCC tissues, PLTs: patients’ autologous para‐cancerous liver tissues.

The immune‐reactivity differences between HCTs and PLTs groups were estimated using Student's t‐test. p< 0.01 was considered statistically significant.

Percentage: (specific cases/total cases).

Low DDX3 level (+) was scored 1–4, while the high level (++) was more than 4 scores.

We assayed the correlation between SUMO2 and DDX3, the expression and distribution of SUMO2 and DDX3 protein in HBV‐positive HCC tissues. DDX3 protein was distributed in nucleus and cytoplasm, while SUMO2 protein mainly located nucleus (Figure 8D), which indirectly demonstrated that DDX3 was SUMOylated in the nucleus. Moreover, TRIM28 was highly expressed in the nucleus of HBV‐HCC tissues (Figure 8D). Furthermore, compared with early‐staged HBV‐HCC patients, the advanced‐staged HBV‐HCC patients exhibited higher levels of TRIM28 expression in liver tissues (Figure 8E), and the level of S‐DDX3 was also elevated (Figure 8F).

A high expression level of TRIM28 in HBV‐HCC patients indicated a poor prognosis based on the online ACLBI and Kaplan‐Meier plotter database analysis (Figure S7B,C). The single DDX3 protein is not sensitive to serve as a prognostic marker for HBV‐HCC (Figure S7D). However, combined with DDX3 and TRIM28 levels to monitor overall survival prognosis of HBV‐HCC patients, HBV‐HCC patients with high DDX3 level and low TRIM28 expression had a better overall survival (Figure 8G). In addition, the expression levels of *DDX3* and *KLF4* was positively correlated in HCC through LinkedOmics and cBioPortal database analysis (Figure 8H, Figure S7E), which is consistent with conclusion of DDX3 upregulation enhancing *KLF4* mRNA level at the cellular level.

Next, we examined DDX3, TRIM28 protein, and *KLF4* mRNA in two pairs of primary HCC and adjacent nontumoral liver tissues. DDX3 and *KLF4* mRNA levels were down‐regulated in HBV‐HCC tissues as compared with adjacent tissues, whereas TRIM28 level was higher in HBV‐HCC tissues (Figure 8I). Moreover, we transfected pFlag‐DDX3 or pFlag‐DDX3^4KR^ in peripheral blood mononuclear cell (PBMC) extracted from the blood samples of HBV‐HCC to verify *KLF4* mRNAs, which showed that DDX3 and DDX3^4KR^ changed the transcript of *KLF4*. In addition, DDX3^4KR^ induced fewer *KLF4α* (Figure 8J), which was consistent with conclusion of DDX3 SUMOylation promotes *KLF4* alternative splicing.

## 3 Discussion

SUMOylation is a critical PTM process that serves as a rapid and reversible mechanism for host cells against viral infection during antiviral immune responses, which regulates function of antiviral proteins and helps maintain immune balance following infection [40]. Conversely, many viruses have evolved strategies to manipulate the host SUMOylation system to facilitate their own benefit, such as by promoting SUMOylation of viral proteins to enhance function or inhibiting host SUMOylation to aid viral replication [40]. Thus, protein SUMOylation has emerged as a pivotal mechanism in both host antiviral defense and viral escape.

HBV infection is the main cause of liver cancer. About 85% of HCC patients are infected with HBV, and the 5‐year survival rate is low [2]. Antiviral therapy can reduce HBV activation after resection for HCC patients and prolong patient survival [41], but antiviral treatment is long‐term, and some patients experience poor therapeutic response. Herein, we investigated the regulatory mechanisms of SUMOylation in response to HBV infection and in HBV‐HCC, suggesting that targeting protein SUMOylation may represent a potential therapeutic strategy for HBV‐HCC.

DDX3 is an ATP‐dependent RNA helicase that functions in a variety of cellular biological processes. DDX3 participates in innate immune regulation during the early stages of viral infection [25], and is closely associated with virus‐induced cancers [42]. When HBV entered liver cells, the phosphorylation of DDX3 mediated recruitment of IRF3 to the DDX3‐IKKε complex, then contributed to *ifnb* promoter activation and inhibiting viral replication [43]. DDX3 activates TBK1/IRF3 immune signaling pathway to induce the expression of IFN‐β, and then decrease the HBsAg secretion [44]. Moreover, HBV pol is a major protein in the viral life cycle that recognizes 5’ ε stem‐loop structure of the pgRNA and formed a complex with the pgRNA, which played an important role in the formation of the nucleocapsid and initiated the reverse transcriptional reaction [45]. The antiviral protein DDX3 can directly bind to HBV Pol in cytoplasm, thereby inhibiting reverse transcription of the virus and suppressing viral replication [19], consistent with our results.

However, with the persistent replication of HBV in host cells, a large number of HBV produces and accumulates in host cells, and cell immune system is unable to completely clear out HBV. the antiviral protein DDX3 is hijacked by viruses, in order to promote their own replication [42, 46]. HBV Pol could interfere with the interaction between DDX3 and IKKε/TBK1, affect the TBK1/IRF3 pathway, and inhibit the production of IFN‐β [21]. Our study indicated that HBV Pol accumulation disrupts the interaction between DDX3 and SENP1, which may affect DDX3 SUMOylation levels in the transformation for chronic HBV to HBV‐HCC.

The types of PTMs and PTMs‐related enzymes modulate DDX3 activity and alter the interactions between DDX3 and other proteins, thereby playing a role in regulating cellular pathways [47]. For example, Sirtuin5 (Sirt5) interacts with DDX3 and demalonylates DDX3, which is critical for TBK1‐IRF3 activation and promotes antiviral innate immune responses [48]. E3 ubiquitin ligase RNF39 inhibits retinoic acid‐inducible gene‐I (RIG‐I)‐like receptor pathways through mediating K48‐linked ubiquitination and proteasomal degradation of DDX3, thereby promoting viral replication [49]. Considering the critical regulatory role of DDX3 in HBV infection and replication, our study focuses on investigating the mechanism by which SUMOylation of DDX3 responses to HBV. Our study found that the early HBV infection drives the host DDX3 de‐SUMOylation. SUMOylation is reversible and dynamic process, in which the modified proteins can be deSUMOylated by SENPs. Based on our conclusion that HBV induces DDX3 deSUMOylation, we screened the SENP family [22], and only SENP1 was found to interact with DDX3. As a key de‐SUMOylating enzyme, SENP1 exhibits extensive subcellular localization, with distribution in the cytoplasm, nucleus, and mitochondria [50]. Our study revealed that SENP1‐mediated deSUMOylation is essential for DDX3 to inhibit HBV gene transcription, HBsAg secretion and cell cycle in HBV cells. However, HBV Pol accumulation disrupts the interaction between DDX3 and SENP1, and SENP1 is downregulated in HBV‐HCC. It is worth noting that the mechanisms by which HBV Pol disrupts the DDX3‐SENP1 interaction and the reduced expression of SENP1 in HBV‐HCC is required further investigation.

The altered levels of host protein SUMOylation appear to represent a host antiviral defense mechanism. For instance, upon viral infection, PIAS3‐induced poly‐SUMOylation promotes polyubiquitination and aggregation of the MAVS protein at lysine 63, thereby efficiently recruiting and releasing IRF3 to facilitate the timely activation of antiviral responses [51]. PIAS1 promotes SUMOylation of the m6A reader YTHDF2 and enhances the anti‐EBV activity of YTHDF2 [52]. Apobec3A is involved in the antiviral host defense, which reduced SUMOylation level of the early viral protein E2A of human adenovirus and disrupted viral replication centers [53]. Thus, de‐SUMOylated DDX3 represents a host defense mechanism against HBV infection.

Notably, viruses also alter the SUMOylation levels of host proteins, thereby suppressing the antiviral immune response. This regulation modulates protein interactions, localization, stability, and transcriptional activity, ultimately controlling viral replication and the viral carcinogenesis process. For instance, SUMOylation of huANP32A/B results in the recruitment of avian influenza virus (AIV) NS2 protein, thereby facilitating huANP32A/B‐supported AIV polymerase activity [54]. DeSUMOylation of the nuclear protein apoptosis inhibitor 5(API5) by Avibirnavirus VP3 protein supports virus replication [55]. HBV primarily expresses the viral proteins HBx, HBcAg, HBsAg, HBV Pol, and HBeAg. HBx protein increases LASP1 SUMOylation to stabilize HER2 and facilitate hepatocarcinogenesis [56]. HBV pol is the earliest discovered and most extensively investigated HBV protein interacting with DDX3 [17]. Our study reveals that the level of DDX3 SUMOylation may be influenced by HBV Pol amounts. DDX3 protein directly bound with HBV Pol to exert antiviral effect by inhibiting HBsAg secretion and promoting p‐IRF3 expression. However, HBV Pol accumulation disrupts the antiviral activity of DDX3 and its interaction with SENP1, leading to upregulation of DDX3 SUMOylation in HBV‐HCC. These dynamic changes in DDX3 SUMOylation levels suggest a role in the transition from liver inflammation to HCC.

DDX3 functions as a tumor suppressor gene by decreasing the expression of Cyclin D1 [34] and inhibiting HBV replication in HBV‐HCC [18, 46]. To investigate the regulatory mechanisms of SUMOylated DDX3, we identified its major SUMOylation sites. DDX3 was majorly modified with SUMO2 conjugation at K81, K208, K215 and K564 sites. These four lysine residues were selected based on the combined prediction scores from GPS‐SUMO and SUMOsp 2.0, together with the results of individual single‐site mutations, which alone had only marginal effects on DDX3 function. To maximize the functional impact and facilitate phenotypic analysis, we therefore generated a combined DDX3^4KR^ mutant, which reduced DDX3 SUMOylation rather than a complete loss of SUMO modification levels. Given that DDX3 may contain additional lysine residues that can be SUMOylated, the contribution of other potential sites to DDX3 function remains to be systematically investigated in future studies using lose‐of‐functional point mutations. In general, the level of SUMOylated DDX3 is elevated in HBV‐HCC under catalysis activation of TRIM28. DDX3 SUMOylation promotes *KLF4* to skip in exon 3 to generate more *KLF4α* isoforms, which attenuates DDX3 ability to downregulate Cyclin D1 and arrests cell cycle, and contributes to HBV‐HCC cell proliferation (Figure 9).

**FIGURE 9:**
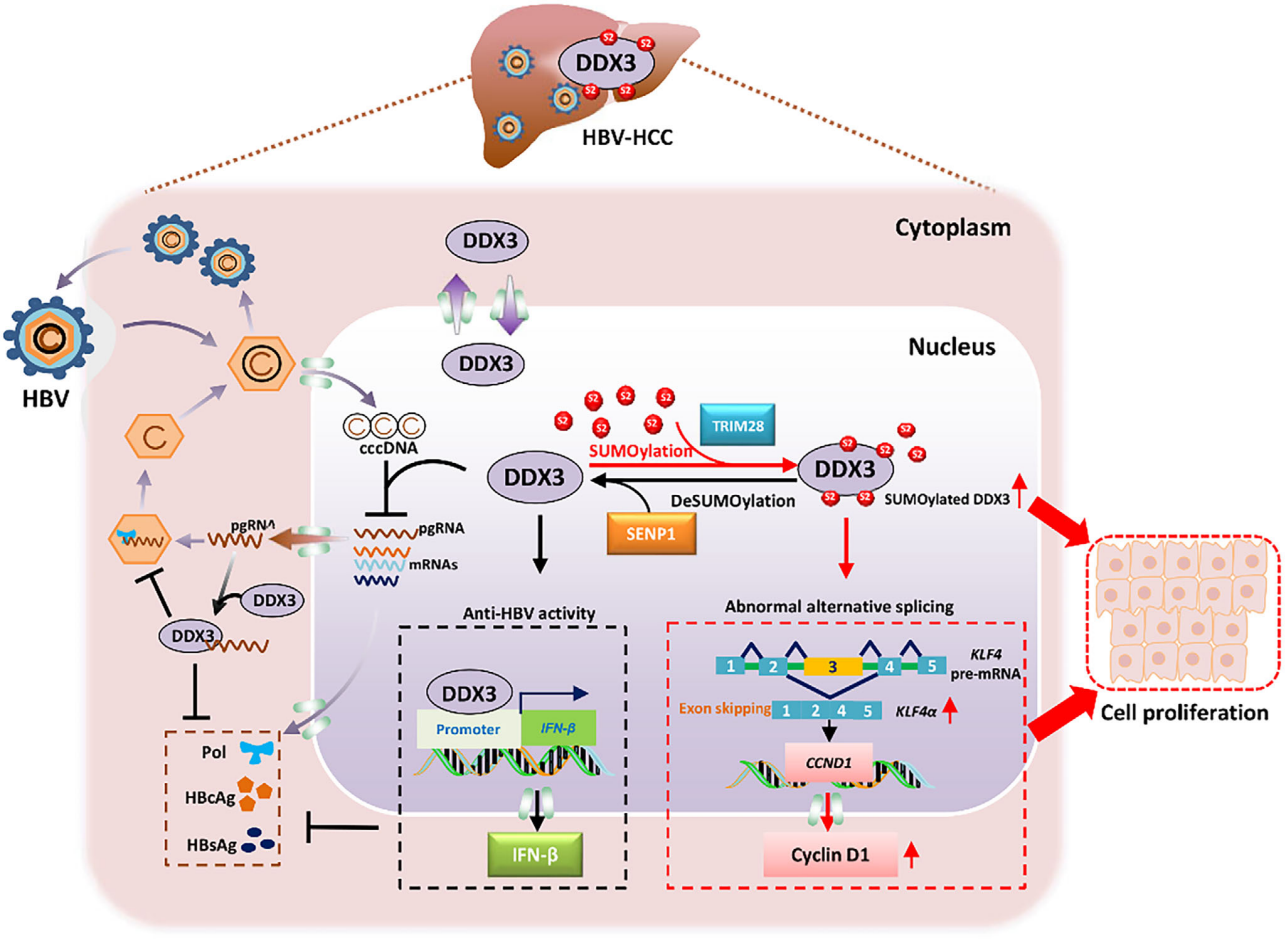
A mechanistic schematic diagram of S‐DDX3 promotes HBV replication and tumor growth of HBV‐HCC. TRIM28 binds to DDX3 protein to catalyze the SUMOylation of DDX3, thus the S‐DDX3 involves in aberrant alternative splicing of *KLF4* mRNA, and the splicing variant *KLF*4α‐mediated cell cycle and signal transduction, weakens tumor suppressor function of DDX3 protein, which promotes progression of HBV‐HCC. Conversely, SENP1 interacts with DDX3 to keep DDX3 deSUMOylation that is essential for inhibiting HBV gene transcription and HBsAg secretion. S‐DDX3: SUMOylated DDX3.

Based on the important biological functions of the DDX3 protein, our study evaluated the expression level of DDX3 and its SUMOylation in HBV‐HCC tissues with association of patient survival prognosis. Although the total DDX3 immunohistochemical score showed only a weak correlation with histopathological grade (Figure 8C), this observation did not weaken the potential importance of DDX3 in the progression of HBV‐HCC. Consequently, we focused on the interaction axis involving DDX3, TRIM28, and SUMO2, as these three molecules functionally reflect the activity of DDX3 SUMOylation. The expression of SUMO ligase TRIM28 was closely correlated with tumor size, tumor stage, and 5‐year overall survival in HCC patients, showing remarkably shorter survival rate in patients with TRIM28 overexpression [57]. Our study showed that the advanced‐staged HBV‐HCC patients had higher levels of TRIM28, SUMO2, and SUMO‐modified DDX3 in liver tissue compared to the early‐staged HBV‐HCC patients. And HBV‐HCC patients with high DDX3 and low TRIM28 levels had a better overall survival. Therefore, the abnormal interaction between TRIM28 and DDX3 may cooperate with DDX3 to promote the development of HCC, which indicates the SUMOylation status of DDX3 may have greater prognostic value than measuring total DDX3 level in HBV‐HCC.

It was worth noting that we applied transient transfection using pHBV‐1.3mer plasmids to mimic early HBV infection in this study. Although this system supports efficient HBV replication and has been widely used to study virus–host interactions, it does not fully replicate the complete HBV life cycle, including viral entry, cccDNA formation, and early host responses associated with natural infection. To validate the accuracy of our findings, we examined and compared the expression levels of SENP1 and TRIM28 in human liver tissue from patients with different clinical backgrounds (no hepatitis vs chronic hepatitis B, early‐stage HCC vs advanced‐stage HCC). Consistent with our in vitro observations, SENP1 expression was reduced in hepatitis tissues compared to non‐hepatitis tissues, and TRIM28 expression was increased with HBV‐HCC progression.

In summary, our study has revealed that DDX3 as a SUMOylation substrate related to HBV‐HCC progression, and explored the molecular mechanism of SUMOylation‐mediated DDX3 in response to HBV replication and in regulating HBV‐HCC cellular biological processes (Figure 9). And our findings indicate targeting TRIM28‐DDX3‐KLF4 signaling axis is a potential effective strategy for treatment of HBV‐HCC.

## 4 Experimental Section

### 4.1 Cell Culture

HEK293T, human HCC cell lines HepG2, HepG2.2.15 and HepAD38 were cultured in DMEM medium supplemented with 10% fetal bovine serum, 100U/mL penicillin and 100 µg/mL streptomycin at 37°C. HepAD38 cells were used for the cell experiments after tetracycline (MCE, HY‐A0107) removal [58].

### 4.2 Plasmids Construction and Cell Transfection

The plasmids pFlag‐DDX3, pHA‐Ubc9, pHA‐ubiquitin, pHis‐SUMO1, pHis‐SUMO2 and pHis‐SUMO3, and pFlag‐SENP1^C603A^ were stored in our laboratory [9, 11, 28]. The truncated sequences of DDX3^NRecA1^, DDX3^∆CTE^, DDX3^∆NTE^, DDX3^CRecA2,^ and DDX3^∆CTE^ were cloned into pTango‐zeo‐N4Flag vector. The site mutations of DDX3, includingK81R, K118R, K138R, K208R, K215R, K418R, K491R, K511R, K564R, K230E and S382L, were generated using Mut Express II Fast Mutagenesis Kit V2 (C214‐01, vazyme). The TRIM28 cDNA (GI: 577858378) and the truncated sequences encoding TRIM28^RBCC^, TRIM28^RING^, TRIM28^PHD,^ and TRIM28^PHDBR^ were cloned into pTango‐zeo‐EGFP vector. The pHBV 1.3‐mer WT replicon (P1178) was purchased from Miaolingbio Technology.

DNA fragment encoding HBV Pol was amplified from pHBV 1.3‐mer WT replicon and cloned into the pTango‐zeo‐EGFP vector to construct pEGFP‐Pol. The plasmids encoding KLF4 and KLF4α were purchased from Sangon Biotech (Shanghai) Co., Ltd. All plasmids were confirmed by DNA sequencing, and transfected into the corresponding cells using the transfection reagent (Lipofectamine 2000, Life Technologies) to observe biological effects.

### 4.3 Lentivirus Infection and Establishment of Stable Overexpression or Knockdown Cell Lines

To generate stable SUMO2‐overexpressing cells, the plasmid pLV203‐mCherry‐SUMO2 was constructed to overexpression SUMO2 gene by a lentivirus delivering system according to the previous report [28, 29]. Briefly, the lentivirus was produced using a three‐plasmid packaging system and transfected in HepG2 cells. Cells were then selected with puromycin (2 µg/ mL) for 1 week. The DDX3‐knockdown HCC cell line was generated with lentivirus infection containing shRNA plasmids targeting DDX3 (shDDX3, targeting sequence: 5’‐ CGCTTGGAACAGGAACTCTTT‐3’) or scrambled sequence (shctrl) for control followed by 2 µg/mL puromycin selection.

### 4.4 Immunoprecipitation

The plasmid constructs and pHis‐SUMOs were transfected into cells as described before to determine which specific SUMO molecule was majorly conjugated with DDX3 in HCC cells [11, 28]. The SUMOylated DDX3 proteins were subsequently pulled down by His tag on Ni^2+^‐NTA affinity beads. Protein immunoprecipitation (IP) assay from cells or tissue slices were performed referring to our previous report [9, 10, 11]. For exogenous IP, cell lysates with approximately 1–2 mg protein were incubated with 10 µL of slurry Anti‐Flag Magnetic Beads (HY‐K0207, MCE) at 4°C for 4 h to enrich Flag‐tagging proteins. For endogenous IP, cell lysates were incubated with anti‐DDX3 or SUMO1 antibody via the protein‐A/G magnetic beads (HY‐K0202, MCE) overnight to enrich DDX3 or SUMO1 binding protein complex. The eluted protein complexes were used for Western blot detection.

### 4.5 Western Blot and Cell Immunofluorescence

The Western blot experiment was conducted in a standardized manner [29, 59]. Cellular proteins were analyzed using SDS‐PAGE gel to test the target protein level by using specific antibodies. The specific primary antibodies included anti‐Flag antibodies (M1403‐2, HuaBio), DDX3(ab196032, Abcam; sc‐365768, Santa Cruz), anti‐SUMO1 antibodies (ab86064, Abcam; ET1606‐53, HuaBio), anti‐SUMO2/3 antibodies (4971, CST; ET1701‐17, HuaBio), SENP1(11929, CST; ET7108‐53, HuaBio), TRIM28(ET1612‐55, HuaBio) and anti‐cyclin D1 (ET1601‐31, HuaBio). The mouse anti‐β‐actin antibody (TA‐09, ZSGB) was used for internal control.

Cellular location of DDX3 was detected by cell immunofluorescence [11]. Cells were incubated with indicated primary antibody overnight at 4°C, following cells were in turn incubated with secondary antibodies (ab150077, ab150080, Abcam) for 1 h, cell nuclei were stained with DAPI for 5 min to visualize by fluorescence microscopy.

### 4.6 Subcellular Fractionation

HCC cells were incubated with 0.5–1.5 µM chemical inhibitor ML‐792 (HY‐108702, MCE) or transfected with pHBV‐1.3mer plasmid for 24 h, then cell cytosolic and nuclear subcellular fractions were isolated with a commercial kit (Nuclear and Cytoplasmic Protein Extraction kit, P0027, Beyotime Institute of Biotechnology, Shanghai, China). The abundance of DDX3 in the nucleus and cytoplasmic was detected by Western blot.

### 4.7 HCC Tissues

The microarray chip with 90 samples of human liver cancer tissues and matched adjacent tissues (HLivH180Su06) was ordered from Outdo Biotech Co., Ltd. (Shanghai, People's Republic of China). The information of HCC tissue microarray was list in Table S4. Twelve liver tissues were collected from Guizhou Provincial People's Hospital with patients’ informed consents. Six cases of HBV‐positive (n = 3) and HBV‐negative (n = 3) liver tissues, which were obtained from the non‐cancerous regions of HCC patients during surgical resection, for use in immunofluorescence experiments to investigate the effect of HBV on SENP1 expression. Additionally, six tumor tissues from HBV‐HCC patients with early‐stage (n = 3) and advanced‐stage (n = 3) were used in immunofluorescence experiments to investigate TRIM28 expression.

### 4.8 Analysis of Online Public Database

Three SUMOylation prediction software, SUMOsp (http://sumosp.biocuckoo.org/), SUMOplot (http://www.abgent.com/sumoplot), and JASSA (http://www.jassa.fr/), were utilized to predict potential SUMOylation sites in DDX3. ACLBI database (https://www.aclbi.com/) and Kaplan–Meier Plotter (https://www.kmplot.com/analysis/) database were used to assess the correlation between DDX3 or TRIM28 expression and overall survival in HBV‐HCC patients. The gene expression correlation analyses were carried out using LinkedOmics (https://www.linkedomics.org/login.php) and cBioPortal (https://www.cbioportal.org).

To evaluate the expression of DDX3 in HCC, the GSE121248, GSE55092, and GSE62232 gene expression profiles were downloaded from the Gene Expression Omnibus (GEO, https://www.ncbi.nlm.nih.gov/geo). The GSE121248 dataset contained 37 adjacent normal tissues and 70 tumoral samples of liver tissues from patients suffering from HCC induced by HBV infection. The GSE62232 dataset included 10 HBV‐related HCC tissues and 10 normal tissues. The GSE55092 dataset included 81 and 39 samples, respectively, from nontumor and tumor areas of liver tissues from HBV‐associated HCC patients. GEO2R (https://www.ncbi.nlm.nih.gov/geo/geo2r/), an interactive internet instrument provided by the National Center for Biotechnical Information (NCBI), was used for identifying DEGs between non‐tumoral and tumoral liver samples from HCC patients with HBV infection. The principle of GEO2R is based on using the GEOquery and limma R packages from the Bioconductor project, which carries out a comparison on processed data tables of microarray data provided by an initial submitter. The DEGs were screened according to adjusted *p* value < 0.05 and |logFC|>2, which were visualized by a volcano plot.

### 4.9 HCC Xenograft Model

All animal studies, including mouse HCC xenograft and the following HBV‐ infected mouse model, were approved by the Animal Care Committee of Sichuan University. Five male BALB/c nude mice, with 5‐week‐old and 20 g body weight for one mouse (HFK bioscience, Beijing, China), were respectively subcutaneously injected 1 × 10^7^ stable DDX3‐overexpressing HepG2 cells to observe tumor growth. A same quantity of HepG2 cells transfected the empty vector plasmid was subcutaneously injected into another five nude mice as a control group. Until approximately 4 weeks later, mouse tumors were harvested *ex vivo* and used to detect target protein expression.

### 4.10 Mouse Model With Acute HBV Infection

Male C57BL/6 mice (GemPharmatech, Nanjing, Jiangsu) with 18–20 g of body weight were established HBV‐infected mouse model by injecting pHBV‐1.3mer plasmid. 15ug plasmids of pHBV‐1.3mer diluted in normal saline were injected into the mouse tail vein in a volume of 10% of the body weight (v/w) within 5–8 s, then monitor the hepatitis B‐related indicators at 0, 1, 2, 6, 8, and 10 days, such as the secretion of HBsAg and the expression of HBcAg in HBV‐infected mouse model.

HBsAg from cellular supernatant or mouse serum was measured using ELISA kits following the manufacturer's protocol (Meimian Biotechnology, Yancheng, China). Absorbance at 450 nm was measured in a microtiter plate reader with dual‐wave length measurement. Formalin‐fixed mouse liver tissues were embedded in paraffin to detect HBcAg by immunohistochemical staining using HBcAg antibody (ab8639, Abcam).

### 4.11 Quantitative RT‐qPCR Analysis

For quantification of HBV *pgRNA* and *IFN‐β*, we extracted total cell RNA and performed RT‐qPCR analysis [29, 31]. The primer sequences used for RT‐qPCR were listed in Supporting Information Table S3.

### 4.12 Association Analysis of DDX3 Level With HCC Clinical Information

The Immunohistochemistry (IHC) staining of DDX3 and SUMO1 was performed following our previous methods [10, 29, 59]. The association of DDX3 expression with HCC clinical information was assessed through the IHC scoring data of HCTs and PLTs using Pearson's χ2 test according to our approach [29]. HCC patients undergoing surgery in the hospital from February 2006 to May 2007. And the HCC clinical information included patient's gender, age, tumor grades and survival time. The clinical information of 90 HCC tissues was shown in detail in Table S4.

### 4.13 Protein Purification and ATPase Activity Assay

Full‐length wild‐type DDX3 and the mutant (DDX3^4KR^) were cloned into pNIC‐His vector and expressed in *E. coli* BL21 (DE3) cells, from which the proteins were purified to measure ATPase activity following our report [31].

### 4.14 RNA Sequencing Analysis

The HepG2 cells with stable knockdown of endogenous DDX3 (shDDX3) cells and shCtrl cells were collected and the total RNA were extracted using Trizol reagent. The samples were then sent to Shanghai Majorbio Bio‐Pharm Technology Co., Ltd for transcriptome sequencing. The sequencing data were analyzed on the online platform of Majorbio Cloud Platform (www.majorbio.com) and DEGs with P adjust value ≤ 0.01 and |log2FC|>1 were considered to be significantly different.

### 4.15 Identification of DDX3‐Binding mRNA by RIP and RIP Sequencing (RIP‐Seq)

RNA‐binding protein immunoprecipitation (RIP) is described in a RIP‐assay kit (RN1001, MBL) according to the manufacturer's protocol. Briefly, 2 × 10^7^ HepG2 cells were transfected plasmids pFlag‐DDX3 and cultured for 48 h. Then cells were washed three times with nuclease PBS and then lysed in RIP lysis buffer containing 1 mM DTT (10708984001, Sigma), 1×protease inhibitor cocktail (P8340, Sigma), 1×PMSF (ST506, Beyotime Biotechnology), and 100U/mL RNase inhibitor (N2515, promega). After centrifugation, the supernatant was added to 15 µg anti‐Flag antibody (ab86064, Abcam) or normal mouse IgG as negative control via the protein‐A/G magnetic beads (HY‐K0202, MCE) overnight to enrich protein‐RNA complexes and wash complexes with wash buffer for 5 min. The enriched RNA for RIP assay and total RNA were subjected to analysis by high‐throughput sequencing (Kangce Technology Co. Inc, Wuhan) or RT‐qPCR.

### 4.16 Dual Luciferase Reporting System

For dual luciferase reporter assay, the cells seeded in 24‐well culture plates were co‐transfected with Flag‐DDX3 (or Flag‐DDX3^4KR^), IFN‐β luciferase plasmids, and pRL‐TK. After transfection for 48 h, the cells were harvested to assess the activities of Renilla luciferase and firefly luciferase using a dual luciferase reporter assay system. The Renilla luciferase signal was normalized to the firefly luciferase signal.

### 4.17 Immunoprecipitation‐Mass Spectrometry (IP‐MS) Analysis

HepG2 cells stably overexpressing SUMO2 or the wild type HepG2 cells were transfected with indicated plasmids for 48 h, and then lysed with Co‐IP buffer. Then the DDX3‐interacting proteins were purified by immunoprecipitation using anti‐Flag magnetic beads (HY‐K0207, MCE). After elution, the co‐immunoprecipitated proteins were digested into peptides with trypsin for liquid chromatography with tandem mass spectrometry (LC–MS/MS) identification as methods we described before [29, 59]. In brief, the original data generated by Orbitrap Fusion Lumos Tribrid mass spectrometer (Thermo Fisher Scientific). Raw data searching was processed in Proteome Discoverer 2.5 (Thermo Fisher Scientific) software or MaxQuant software (version 2.2) with a human protein database. Functional enrichment analysis was conducted via Metascape (http://metascape.org/).

### 4.18 Detection of *KLF4* Specific Isoforms by PCR

Detection of differentially spliced *KLF4* isoforms was performed by RT‐PCR using the following primers, designed to detect the entire coding region of *KLF4* mRNA. Forword: 5’‐ATGGCTGTCAGCGACGCGCTGC‐3’, Reverse: 5’‐TTAAAAATGCCTCTTCATGTGTAAGGCG‐3’. Expected sizes for *KLF4* wild‐type and *KLF4α* are 1410 and 440 bp, respectively. β‐actin gene (*ACTB*) was used as a loading control.

### 4.19 In Vitro SUMOylation Assays

His‐tagged DDX3 and DDX3^4KR^ were expressed in *E. coli* BL21 to purify referencing our previous methods [31]. TRIM28 protein (BA50736) purchased from Biodragon. In vitro SUMOylation of DDX3 was performed using the SUMOylation kit (ab139470, Abcam) according to the manufacturer's instructions. Briefly, the purified protein was mixed as indicated and incubated at a constant temperature of 37°C for 1 h, followed by the addition of 20 µL of non‐reducing SDS gel loading buffer and denaturation at 95°C for 5 min. Finally, samples were separated by SDS‐PAGE and analyzed by Western blot.

### 4.20 Statistics Analysis

Statistical analysis was performed using GraphPad Prism software version 7. Data were represented as mean ± SD. *p* < 0.05 was regarded as a significant difference. Differences between two groups were analyzed by two‐tailed unpaired Student's t‐test or paired Student's t‐test. One‐way or two‐way ANOVA was used for comparing multiple groups.

## Author Contributions

Yanfang Yang: Methodology, Investigation, Data analysis, Writing – original draft, Writing – review and editing, Visualization. Ting Kong, Binghan Wang, Xiaoli Jin, Lan Huang, Nan Zhang, Siyu Tang, Samina ejaz Syed: Investigation, Visualization. Shufang Liang: Methodology, Resources, Writing – original draft, Writing – review & editing, Supervision, Project administration, Funding acquisition. All authors read and approved the final manuscript.

## Ethics Statement

The animal experiments were approved by the Animal Care Committee of Sichuan University (Approval number: 20241127005). The human HCC tissues were collected from those who were surgically resected in the hospital, with the offers’ informed consent guidelines established. Prior review, consent, and approval for this project were provided by the Institutional Ethics Committee (Approval Number: 2025–269). The studies were conducted in accordance with the local legislation and institutional requirements.

## Conflicts of Interest

The authors declare no conflicts of interest.

## Supporting information




**Supporting File**: advs78603‐sup‐0001‐SuppMat.docx.

## Data Availability

The data that supports the findings of this study are available in the supplementary material of this article.
