# Supplementary material for: SUMOylated DDX3 Promotes HBV Replication and Tumor Growth of Hepatocellular Carcinoma via TRIM28‐DDX3‐KLF4α Signaling Axis
Source: Adv Sci (Weinh). 2026 Nov 3:e78603. Online ahead of print. doi: 10.1002/advs.78603 (PMC13731071; doi:10.1002/advs.78603)
Supplement: Supplementary file 1 [file ADVS-9999-e78603-s001.docx]

**SUMOylated DDX3 promotes HBV replication and tumor growth of hepatocellular carcinoma via TRIM28-DDX3-KLF4α signaling axis**

*Yanfang Yang^1,2^, Ting Kong^1^, Binghan Wang^3^, Xiaoli Jin^1^, Lan Huang^1^, Nan Zhang^1, 4^, Siyu Tang^5,6^, Samina Ejaz Syed^7^, and Shufang Liang^1*^*

1 Department of Biotherapy, Cancer Center and State Key Laboratory of Biotherapy, West China Hospital, Sichuan University, Chengdu 610041, China.
2 Department of Central Laboratory, NHC Key Laboratory of Pulmonary Immunological Diseases, Guizhou Provincial People's Hospital, Guiyang 550002, China.
3 Department of Neurology, Second Affiliated Hospital of Army Medical University (Xinqiao Hospital), Chongqing 400037, China.
4 Department of Medical Oncology, Cancer Center, West China Hospital, Sichuan University, Chengdu 610041, China.
5 Department of Gastrointestinal Surgery, West China Hospital, Sichuan University, Chengdu 610041, China.
6 The Second Department of Gastrointestinal Surgery, the Affiliated Hospital of North Sichuan Medical College, Sichuan, China.
7 Department of Biochemistry and Biotechnology, Baghdad Campus, The Islamia University of Bahawalpur, Bahawalpur, Pakistan.

*Correspondence to*: Dr. Shufang Liang, State Key Laboratory of Biotherapy and Cancer Center, West China Hospital, Sichuan University, and Collaborative Innovation Center for Biotherapy, No.17, 3rd Section of People's South Road, Chengdu, 610041, P. R. China. E-mail: [zizi2006@scu.edu.cn](mailto:zizi2006@scu.edu.cn).

**Supplementary Figures**

**Figure S1.** (A) The total SUMOylation levels were attenuated in a dose-dependent manner in HepG2 and Hep3B cells under treatment with different concentrations of ML-792 inhibitor for 48h.

**Figure S2.** **Sequence alignment analysis of Lys at multiple amino acid sites, including 81, 118, 138, 208, 215, 418, 491, 511 and 564, in DDX3 among various species.**

**Figure S3.** (A) The level of S-DDX3 decreased along with the increase of HBV plasmid transfection concentration. (B) Levels of IRF3 phosphorylation (p-IRF3) and IFN-β secretion in HepG2 or Hep3B cells under treatment with DNA virus analogue Poly(dA:dT). (C) The DNA analogues poly (dA: dT) weakened the SUMO-DDX3 interaction and S-DDX3 level. After HepG2 cells were treated with 1μg poly (dA: dT) for 12h, we enriched S-DDX3 by IP to detect. (D) Levels of p-IRF3 and IFN-β secretion in HepG2 or Hep3B cells under treatment with RNA virus analogue Poly(I:C). (E) The RNA viral analogues poly (I: C) weakened S-DDX3 level. After HepG2 cells were treated with 1.5μg poly (I: C) for 12h, we enriched S-DDX3 by IP to detect. (F) Upregulation of SENP1 reduced S-DDX3 level with a dose-dependent manner in HepG2 cells. HepG2 cells were transfected with pFlag-DDX3, pHA-Ubc9 and different amounts of pFlag-SENP1, then the S-DDX3 was analyzed by Western blotting with indicated antibodies. S-DDX3: SUMO modified DDX3. IP: immunoprecipitation, IB: immunoblot.

**Figure S4.** (A) DDX3 inhibited the secretion level of HBsAg in pHBV transfecting-replication cells. (B) DDX3^4KR^ had higher antiviral activity of suppressing pgRNA expression and HBsAg secretion than DDX3 in pHBV transfecting-replication cells. The plasmids pHBV-1.3mer with pFlag-DDX3, pFlag-DDX3^4KR^ were respectively transfected into Hep3B cells for 48h, then HBV transcription intermediate pgRNA and HBsAg secretion were detected by RT-qPCR and Elisa. (C) Schematic diagram of the establishment of acute HBV-infecting mouse model by hydrodynamic injection (HI) of 10μg pHBV-1.3mer plasmids in C57BL/6 mice. (D-F) Time-dependent HBsAg secretion and HBcAg expression in HBV-infecting mice. HBsAg in the serum were measured by Elisa, and HBcAg were detected by IHC at different time points (0, 1, 2, 6, 8 and 10 days). (G-H) DDX3 inhibits HBsAg secretion and HBcAg expression in pHBV-1.3mer-injected mice. The expression of HBcAg in mouse liver was observed by IHC (G) and western blot (H). Scale bar: 50 μm.

**Figure S5. DDX3 SUMOylation favors exon skipping of *KLF4* mRNA to promote HBV-HCC cell cycle progression.** (A) Overexpression of DDX3 restrained HBV-HCC cells in the G1/S phase. The plasmids pHBV-1.3mer and pFlag-DDX3 were transfected to HepG2 cells for 48h, then cell cycle was detected by flow cytometry. (B) DDX3 interacted with mRNAs. The plasmid pFlag-DDX3 was transfected in HepG2 cells for 48 h, then preformed RNA immunoprecipitation sequencing (RIP-seq). The absolute value of logFC > 1 and p value < 0.05 were used as criteria to select genes with up-regulated IP relative to input as protein binding genes. (C) The major binding regions of DDX3 with mRNAs. The horizontal coordinates represent the 5‘UTR, CDS, and 3’UTR regions of the genes, and the vertical coordinates represent the depth of reads coverage compared to all genes. (D) KEGG pathway enrichment analysis of DDX3-binding genes. (E) Identification of DDX3 interacting proteins under SUMO2 overexpression conditions. A Venn diagram illustrating number of DDX3 interactors. The DDX3 interactors in the context of SUMO2 overexpression were selected and the list of KEGG pathway annotation of selected interactors. Flag-control: The group was transiently transfected with the empty vector pFlag into HepG2 cells. Flag-DDX3: The group was transiently transfected with plasmids pFlag-DDX3 into HepG2 cells. Flag-DDX3+SUMO2: The group was transiently co-transfected plasmids pFlag-DDX3 with pHis-SUMO2 into HepG2 cells. (F) Changes in cell cycle distribution of HCC cells with stably overexpressing SUMO2 (HepG2^His-SUMO2^) were detected by flow cytometry. (G) The expression of *KLF13* and *KLF17* in HCC cells transfected with pFlag-DDX3 plasmids were detected by RT-qPCR. (H) DDX3 bound to cell cycle regulator *KLFs* mRNA. IP assay with HepG2 cell lysates expressing pFlag-tagged DDX3 plasmids by using anti-Flag antibody. Immunoprecipitated *KLF13* and *KLF17* mRNA were quantitated by RT-qPCR and normalized to the amount of immunoprecipated proteins. (I) *KLF4* mRNA has five splicing variants through TCGA-LIHC dataset analysis from TSVdb database. LIHC, Liver hepatocellular carcinoma. (J) DDX3 regulates *KLF4* mRNA skipping at exon 3, producing spliced isoform KLF4α. (G) HepG2.2.15 cells were transiently transfected with pFlag-KLF4 or pFlag-KLF4α for 48h, and the expression distribution was detected by immunofluorescence. (K) HepG2.2.15 cells were transiently transfected with pEGFP-KLF4 or pEGFP-KLF4α for 48h, then KLF4 distribution was examined by immunofluorescence.

**Figure S6.** (A) TRIM28 promotes the levels of SUMO2-modified substrate proteins. (B) TRIM28 promotes the level of S-DDX3. The plasmids pFlag-DDX3, pHA-Ubc9 and different concentrations of pMyc-TRIM28 were co-transfected into HepG2 or HEK293T cells, and the proteins were collected after 48 h for co-IP. S-DDX3: SUMO modified DDX3. IP: immunoprecipitation, IB: immunoblot.

**Figure S7.** (A) GEO database analysis of *DDX3* mRNA expression levels in HBV-HCC. (E) Low TRIM28 expression levels have better prognostic value for HBV-HCC patients using Kaplan–Meier plotter online tools. (B-C) Low TRIM28 expression levels have better prognostic value for HBV-HCC patients using the online tools of ACLBI and Kaplan–Meier plotter. (D) DDX3 expression levels have no prognostic value for HBV-HCC patients using ACLBI database. (E) Gene expression correlation between *DDX3* and *KLF4* in HCC from LinkedOmics online database.(F) The order of microarray sites of 90 HCC and adjacent tissue was included. Blue dots represent HCC tissues and green dots represent adjacent tissues. (G) The HE staining in 90 cases of HCC and adjacent tissues. (H) The immunohistochemical staining of DDX3 in 90 cases of HCC and adjacent tissues.

Supplementary Tables

Table S1. The DDX3 interactors in the context of SUMO2 overexpression.

| **Accession** | **Description** | **Coverage [%]** | **Peptides** | **PSMs** | **Unique Peptides** | **AAs** | **MW [kDa]** | **calc. pI** | **Score Sequest HT: Sequest HT** | **Peptides (by Search Engine): Sequest HT** | **Protein Groups** |
| --- | --- | --- | --- | --- | --- | --- | --- | --- | --- | --- | --- |
| P68133 | Actin, alpha skeletal muscle OS=Homo sapiens OX=9606 GN=ACTA1 PE=1 SV=1 | 39 | 14 | 17 | 3 | 377 | 42 | 5.39 | 47.89 | 14 | 1 |
| P33991 | DNA replication licensing factor MCM4 OS=Homo sapiens OX=9606 GN=MCM4 PE=1 SV=5 | 5 | 4 | 4 | 4 | 863 | 96.5 | 6.74 | 9.11 | 4 | 1 |
| P27708 | CAD protein OS=Homo sapiens OX=9606 GN=CAD PE=1 SV=3 | 2 | 3 | 3 | 2 | 2225 | 242.8 | 6.46 | 7.07 | 3 | 1 |
| Q92900 | Regulator of nonsense transcripts 1 OS=Homo sapiens OX=9606 GN=UPF1 PE=1 SV=2 | 3 | 3 | 3 | 3 | 1129 | 124.3 | 6.61 | 6.6 | 3 | 1 |
| Q86TG7 | Retrotransposon-derived protein PEG10 OS=Homo sapiens OX=9606 GN=PEG10 PE=1 SV=2 | 4 | 2 | 2 | 2 | 708 | 80.1 | 6.39 | 5.6 | 2 | 1 |
| O75396 | Vesicle-trafficking protein SEC22b OS=Homo sapiens OX=9606 GN=SEC22B PE=1 SV=4 | 13 | 2 | 2 | 2 | 215 | 24.6 | 6.92 | 4.67 | 2 | 1 |
| O95782 | AP-2 complex subunit alpha-1 OS=Homo sapiens OX=9606 GN=AP2A1 PE=1 SV=3 | 2 | 2 | 2 | 2 | 977 | 107.5 | 7.03 | 4.65 | 2 | 1 |
| Q04837 | Single-stranded DNA-binding protein, mitochondrial OS=Homo sapiens OX=9606 GN=SSBP1 PE=1 SV=1 | 16 | 2 | 2 | 2 | 148 | 17.2 | 9.6 | 4.59 | 2 | 1 |
| O95831 | Apoptosis-inducing factor 1, mitochondrial OS=Homo sapiens OX=9606 GN=AIFM1 PE=1 SV=1 | 4 | 2 | 2 | 2 | 613 | 66.9 | 8.95 | 4.5 | 2 | 1 |
| P00533 | Epidermal growth factor receptor OS=Homo sapiens OX=9606 GN=EGFR PE=1 SV=2 | 2 | 2 | 2 | 2 | 1210 | 134.2 | 6.68 | 4.2 | 2 | 1 |
| P14868 | Aspartate--tRNA ligase, cytoplasmic OS=Homo sapiens OX=9606 GN=DARS1 PE=1 SV=2 | 4 | 2 | 2 | 2 | 501 | 57.1 | 6.55 | 4.06 | 2 | 1 |
| Q7L0Y3 | tRNA methyltransferase 10 homolog C OS=Homo sapiens OX=9606 GN=TRMT10C PE=1 SV=2 | 4 | 2 | 2 | 2 | 403 | 47.3 | 9.36 | 4.05 | 2 | 1 |
| P61221 | ATP-binding cassette sub-family E member 1 OS=Homo sapiens OX=9606 GN=ABCE1 PE=1 SV=1 | 4 | 2 | 2 | 2 | 599 | 67.3 | 8.34 | 4.01 | 2 | 1 |
| O43776 | Asparagine--tRNA ligase, cytoplasmic OS=Homo sapiens OX=9606 GN=NARS1 PE=1 SV=1 | 3 | 2 | 2 | 2 | 548 | 62.9 | 6.25 | 3.97 | 2 | 1 |
| O43823 | A-kinase anchor protein 8 OS=Homo sapiens OX=9606 GN=AKAP8 PE=1 SV=1 | 3 | 1 | 1 | 1 | 692 | 76.1 | 5.15 | 3.38 | 1 | 1 |
| A6NMY6 | Putative annexin A2-like protein OS=Homo sapiens OX=9606 GN=ANXA2P2 PE=5 SV=2 | 3 | 1 | 1 | 1 | 339 | 38.6 | 6.95 | 3.36 | 1 | 1 |
| P26640 | Valine--tRNA ligase OS=Homo sapiens OX=9606 GN=VARS1 PE=1 SV=4 | 1 | 1 | 1 | 1 | 1264 | 140.4 | 7.59 | 3.25 | 1 | 1 |
| O43175 | D-3-phosphoglycerate dehydrogenase OS=Homo sapiens OX=9606 GN=PHGDH PE=1 SV=4 | 3 | 1 | 1 | 1 | 533 | 56.6 | 6.71 | 3.07 | 1 | 1 |
| P62877 | E3 ubiquitin-protein ligase RBX1 OS=Homo sapiens OX=9606 GN=RBX1 PE=1 SV=1 | 18 | 1 | 1 | 1 | 108 | 12.3 | 6.96 | 3.04 | 1 | 1 |
| O14617 | AP-3 complex subunit delta-1 OS=Homo sapiens OX=9606 GN=AP3D1 PE=1 SV=1 | 1 | 1 | 1 | 1 | 1153 | 130.1 | 8.48 | 2.94 | 1 | 1 |
| Q16762 | Thiosulfate sulfurtransferase OS=Homo sapiens OX=9606 GN=TST PE=1 SV=4 | 6 | 1 | 1 | 1 | 297 | 33.4 | 7.25 | 2.94 | 1 | 1 |
| O75746 | Calcium-binding mitochondrial carrier protein Aralar1 OS=Homo sapiens OX=9606 GN=SLC25A12 PE=1 SV=2 | 2 | 1 | 1 | 1 | 678 | 74.7 | 8.38 | 2.93 | 1 | 1 |
| Q9BQ52 | Zinc phosphodiesterase ELAC protein 2 OS=Homo sapiens OX=9606 GN=ELAC2 PE=1 SV=2 | 2 | 1 | 1 | 1 | 826 | 92.2 | 7.9 | 2.72 | 1 | 1 |
| Q13410 | Butyrophilin subfamily 1 member A1 OS=Homo sapiens OX=9606 GN=BTN1A1 PE=1 SV=3 | 2 | 1 | 1 | 1 | 526 | 58.9 | 5.54 | 2.69 | 1 | 1 |
| P51659 | Peroxisomal multifunctional enzyme type 2 OS=Homo sapiens OX=9606 GN=HSD17B4 PE=1 SV=3 | 2 | 1 | 1 | 1 | 736 | 79.6 | 8.84 | 2.68 | 1 | 1 |
| Q96IX5 | ATP synthase membrane subunit DAPIT, mitochondrial OS=Homo sapiens OX=9606 GN=ATP5MD PE=1 SV=1 | 28 | 1 | 1 | 1 | 58 | 6.5 | 9.76 | 2.66 | 1 | 1 |
| P53007 | Tricarboxylate transport protein, mitochondrial OS=Homo sapiens OX=9606 GN=SLC25A1 PE=1 SV=2 | 4 | 1 | 1 | 1 | 311 | 34 | 9.89 | 2.57 | 1 | 1 |
| O60832 | H/ACA ribonucleoprotein complex subunit DKC1 OS=Homo sapiens OX=9606 GN=DKC1 PE=1 SV=3 | 3 | 1 | 1 | 1 | 514 | 57.6 | 9.42 | 2.55 | 1 | 1 |
| P26599 | Polypyrimidine tract-binding protein 1 OS=Homo sapiens OX=9606 GN=PTBP1 PE=1 SV=1 | 2 | 1 | 1 | 1 | 531 | 57.2 | 9.17 | 2.54 | 1 | 1 |
| P01116 | GTPase KRas OS=Homo sapiens OX=9606 GN=KRAS PE=1 SV=1 | 6 | 1 | 1 | 1 | 189 | 21.6 | 6.77 | 2.53 | 1 | 1 |
| Q6PKG0 | La-related protein 1 OS=Homo sapiens OX=9606 GN=LARP1 PE=1 SV=2 | 1 | 1 | 1 | 1 | 1096 | 123.4 | 8.82 | 2.51 | 1 | 1 |
| Q15006 | ER membrane protein complex subunit 2 OS=Homo sapiens OX=9606 GN=EMC2 PE=1 SV=1 | 4 | 1 | 1 | 1 | 297 | 34.8 | 6.57 | 2.51 | 1 | 1 |
| P36897 | TGF-beta receptor type-1 OS=Homo sapiens OX=9606 GN=TGFBR1 PE=1 SV=1 | 3 | 1 | 1 | 1 | 503 | 55.9 | 7.55 | 2.51 | 1 | 1 |
| Q9H0U4 | Ras-related protein Rab-1B OS=Homo sapiens OX=9606 GN=RAB1B PE=1 SV=1 | 7 | 1 | 1 | 1 | 201 | 22.2 | 5.73 | 2.5 | 1 | 1 |
| P56192 | Methionine--tRNA ligase, cytoplasmic OS=Homo sapiens OX=9606 GN=MARS1 PE=1 SV=2 | 1 | 1 | 1 | 1 | 900 | 101.1 | 6.16 | 2.49 | 1 | 1 |
| Q8N726 | Tumor suppressor ARF OS=Homo sapiens OX=9606 GN=CDKN2A PE=1 SV=2 | 6 | 1 | 1 | 1 | 132 | 13.9 | 12.41 | 2.48 | 1 | 1 |
| P37108 | Signal recognition particle 14 kDa protein OS=Homo sapiens OX=9606 GN=SRP14 PE=1 SV=2 | 10 | 1 | 1 | 1 | 136 | 14.6 | 10.04 | 2.44 | 1 | 1 |
| P53992 | Protein transport protein Sec24C OS=Homo sapiens OX=9606 GN=SEC24C PE=1 SV=3 | 1 | 1 | 1 | 1 | 1094 | 118.2 | 7.06 | 2.42 | 1 | 1 |
| P48643 | T-complex protein 1 subunit epsilon OS=Homo sapiens OX=9606 GN=CCT5 PE=1 SV=1 | 2 | 1 | 1 | 1 | 541 | 59.6 | 5.66 | 2.42 | 1 | 1 |
| Q9NXF1 | Testis-expressed protein 10 OS=Homo sapiens OX=9606 GN=TEX10 PE=1 SV=2 | 1 | 1 | 1 | 1 | 929 | 105.6 | 9.36 | 2.42 | 1 | 1 |
| Q9UHB6 | LIM domain and actin-binding protein 1 OS=Homo sapiens OX=9606 GN=LIMA1 PE=1 SV=1 | 1 | 1 | 1 | 1 | 759 | 85.2 | 6.84 | 2.42 | 1 | 1 |
| P19367 | Hexokinase-1 OS=Homo sapiens OX=9606 GN=HK1 PE=1 SV=3 | 1 | 1 | 1 | 1 | 917 | 102.4 | 6.8 | 2.4 | 1 | 1 |
| P46782 | 40S ribosomal protein S5 OS=Homo sapiens OX=9606 GN=RPS5 PE=1 SV=4 | 4 | 1 | 1 | 1 | 204 | 22.9 | 9.72 | 2.39 | 1 | 1 |
| P78371 | T-complex protein 1 subunit beta OS=Homo sapiens OX=9606 GN=CCT2 PE=1 SV=4 | 2 | 1 | 1 | 1 | 535 | 57.5 | 6.46 | 2.38 | 1 | 1 |
| O43172 | U4/U6 small nuclear ribonucleoprotein Prp4 OS=Homo sapiens OX=9606 GN=PRPF4 PE=1 SV=2 | 3 | 1 | 1 | 1 | 522 | 58.4 | 7.42 | 2.38 | 1 | 1 |
| P35250 | Replication factor C subunit 2 OS=Homo sapiens OX=9606 GN=RFC2 PE=1 SV=3 | 4 | 1 | 1 | 1 | 354 | 39.1 | 6.44 | 2.36 | 1 | 1 |
| P02794 | Ferritin heavy chain OS=Homo sapiens OX=9606 GN=FTH1 PE=1 SV=2 | 6 | 1 | 1 | 1 | 183 | 21.2 | 5.55 | 2.33 | 1 | 1 |
| P11498 | Pyruvate carboxylase, mitochondrial OS=Homo sapiens OX=9606 GN=PC PE=1 SV=2 | 1 | 1 | 1 | 1 | 1178 | 129.6 | 6.84 | 2.33 | 1 | 1 |
| O60701 | UDP-glucose 6-dehydrogenase OS=Homo sapiens OX=9606 GN=UGDH PE=1 SV=1 | 3 | 1 | 1 | 1 | 494 | 55 | 7.12 | 2.33 | 1 | 1 |
| Q9NSE4 | Isoleucine--tRNA ligase, mitochondrial OS=Homo sapiens OX=9606 GN=IARS2 PE=1 SV=2 | 2 | 1 | 1 | 1 | 1012 | 113.7 | 7.2 | 2.32 | 1 | 1 |
| P47989 | Xanthine dehydrogenase/oxidase OS=Homo sapiens OX=9606 GN=XDH PE=1 SV=4 | 1 | 1 | 1 | 1 | 1333 | 146.3 | 7.66 | 2.31 | 1 | 1 |
| P27348 | 14-3-3 protein theta OS=Homo sapiens OX=9606 GN=YWHAQ PE=1 SV=1 | 6 | 1 | 1 | 1 | 245 | 27.7 | 4.78 | 2.3 | 1 | 1 |
| Q8TC12 | Retinol dehydrogenase 11 OS=Homo sapiens OX=9606 GN=RDH11 PE=1 SV=2 | 4 | 1 | 1 | 1 | 318 | 35.4 | 8.82 | 2.3 | 1 | 1 |
| O95707 | Ribonuclease P protein subunit p29 OS=Homo sapiens OX=9606 GN=POP4 PE=1 SV=2 | 6 | 1 | 1 | 1 | 220 | 25.4 | 10.07 | 2.29 | 1 | 1 |
| Q9Y388 | RNA-binding motif protein, X-linked 2 OS=Homo sapiens OX=9606 GN=RBMX2 PE=1 SV=2 | 3 | 1 | 1 | 1 | 322 | 37.3 | 9.83 | 2.29 | 1 | 1 |
| Q9P015 | 39S ribosomal protein L15, mitochondrial OS=Homo sapiens OX=9606 GN=MRPL15 PE=1 SV=1 | 4 | 1 | 1 | 1 | 296 | 33.4 | 10.01 | 2.28 | 1 | 1 |
| Q96CS3 | FAS-associated factor 2 OS=Homo sapiens OX=9606 GN=FAF2 PE=1 SV=2 | 2 | 1 | 1 | 1 | 445 | 52.6 | 5.62 | 2.25 | 1 | 1 |
| Q16891 | MICOS complex subunit MIC60 OS=Homo sapiens OX=9606 GN=IMMT PE=1 SV=1 | 1 | 1 | 1 | 1 | 758 | 83.6 | 6.48 | 2.24 | 1 | 1 |
| P50750 | Cyclin-dependent kinase 9 OS=Homo sapiens OX=9606 GN=CDK9 PE=1 SV=3 | 3 | 1 | 1 | 1 | 372 | 42.8 | 8.79 | 2.24 | 1 | 1 |
| Q16543 | Hsp90 co-chaperone Cdc37 OS=Homo sapiens OX=9606 GN=CDC37 PE=1 SV=1 | 2 | 1 | 1 | 1 | 378 | 44.4 | 5.25 | 2.23 | 1 | 1 |
| O15160 | DNA-directed RNA polymerases I and III subunit RPAC1 OS=Homo sapiens OX=9606 GN=POLR1C PE=1 SV=1 | 5 | 1 | 1 | 1 | 346 | 39.2 | 5.5 | 2.22 | 1 | 1 |
| P46977 | Dolichyl-diphosphooligosaccharide—protein glycosyltransferase subunit STT3A OS=Homo sapiens OX=9606 GN=STT3A PE=1 SV=2 | 2 | 1 | 1 | 1 | 705 | 80.5 | 8.07 | 2.21 | 1 | 1 |
| Q96TA2 | ATP-dependent zinc metalloprotease YME1L1 OS=Homo sapiens OX=9606 GN=YME1L1 PE=1 SV=2 | 2 | 1 | 1 | 1 | 773 | 86.4 | 8.76 | 2.17 | 1 | 1 |
| Q16531 | DNA damage-binding protein 1 OS=Homo sapiens OX=9606 GN=DDB1 PE=1 SV=1 | 1 | 1 | 1 | 1 | 1140 | 126.9 | 5.26 | 2.13 | 1 | 1 |
| O15143 | Actin-related protein 2/3 complex subunit 1B OS=Homo sapiens OX=9606 GN=ARPC1B PE=1 SV=3 | 3 | 1 | 1 | 1 | 372 | 40.9 | 8.35 | 2.13 | 1 | 1 |
| Q9H7B2 | Ribosome production factor 2 homolog OS=Homo sapiens OX=9606 GN=RPF2 PE=1 SV=2 | 3 | 1 | 1 | 1 | 306 | 35.6 | 9.99 | 2.12 | 1 | 1 |
| Q99623 | Prohibitin-2 OS=Homo sapiens OX=9606 GN=PHB2 PE=1 SV=2 | 4 | 1 | 1 | 1 | 299 | 33.3 | 9.83 | 2.12 | 1 | 1 |
| Q96N66 | Lysophospholipid acyltransferase 7 OS=Homo sapiens OX=9606 GN=MBOAT7 PE=1 SV=2 | 3 | 1 | 1 | 1 | 472 | 52.7 | 8.97 | 2.12 | 1 | 1 |
| Q71RC2 | La-related protein 4 OS=Homo sapiens OX=9606 GN=LARP4 PE=1 SV=3 | 2 | 1 | 1 | 1 | 724 | 80.5 | 6.61 | 2.11 | 1 | 1 |
| P04181 | Ornithine aminotransferase, mitochondrial OS=Homo sapiens OX=9606 GN=OAT PE=1 SV=1 | 3 | 1 | 1 | 1 | 439 | 48.5 | 7.03 | 2.1 | 1 | 1 |
| A1L0T0 | 2-hydroxyacyl-CoA lyase 2 OS=Homo sapiens OX=9606 GN=ILVBL PE=1 SV=2 | 1 | 1 | 1 | 1 | 632 | 67.8 | 8.15 | 2.1 | 1 | 1 |
| P62140 | Serine/threonine-protein phosphatase PP1-beta catalytic subunit OS=Homo sapiens OX=9606 GN=PPP1CB PE=1 SV=3 | 3 | 1 | 1 | 1 | 327 | 37.2 | 6.19 | 2.1 | 1 | 1 |
| Q9UHD8 | Septin-9 OS=Homo sapiens OX=9606 GN=SEPTIN9 PE=1 SV=2 | 3 | 1 | 1 | 1 | 586 | 65.4 | 8.97 | 2.1 | 1 | 1 |
| P43034 | Platelet-activating factor acetylhydrolase IB subunit alpha OS=Homo sapiens OX=9606GN=PAFAH1B1 PE=1 SV=2 | 3 | 1 | 1 | 1 | 410 | 46.6 | 7.37 | 2.09 | 1 | 1 |
| Q13217 | DnaJ homolog subfamily C member 3 OS=Homo sapiens OX=9606 GN=DNAJC3 PE=1 SV=1 | 2 | 1 | 1 | 1 | 504 | 57.5 | 6.15 | 2.08 | 1 | 1 |
| P61026 | Ras-related protein Rab-10 OS=Homo sapiens OX=9606 GN=RAB10 PE=1 SV=1 | 6 | 1 | 1 | 1 | 200 | 22.5 | 8.38 | 2.08 | 1 | 1 |
| Q96I59 | Probable asparagine--tRNA ligase, mitochondrial OS=Homo sapiens OX=9606 GN=NARS2 PE=1 SV=3 | 3 | 1 | 1 | 1 | 477 | 54.1 | 7.24 | 2.06 | 1 | 1 |
| O15212 | Prefoldin subunit 6 OS=Homo sapiens OX=9606 GN=PFDN6 PE=1 SV=1 | 6 | 1 | 1 | 1 | 129 | 14.6 | 8.88 | 2.04 | 1 | 1 |
| Q14254 | Flotillin-2 OS=Homo sapiens OX=9606 GN=FLOT2 PE=1 SV=2 | 3 | 1 | 1 | 1 | 428 | 47 | 5.25 | 2.04 | 1 | 1 |
| Q9Y399 | 28S ribosomal protein S2, mitochondrial OS=Homo sapiens OX=9606 GN=MRPS2 PE=1 SV=1 | 3 | 1 | 1 | 1 | 296 | 33.2 | 9.26 | 2.03 | 1 | 1 |
| P43897 | Elongation factor Ts, mitochondrial OS=Homo sapiens OX=9606 GN=TSFM PE=1 SV=2 | 2 | 1 | 1 | 1 | 325 | 35.4 | 8.38 | 2.02 | 1 | 1 |
| Q15070 | Mitochondrial inner membrane protein OXA1L OS=Homo sapiens OX=9606 GN=OXA1L PE=1 SV=3 | 2 | 1 | 1 | 1 | 435 | 48.5 | 9.45 | 2.02 | 1 | 1 |
| O14684 | Prostaglandin E synthase OS=Homo sapiens OX=9606 GN=PTGES PE=1 SV=2 | 7 | 1 | 1 | 1 | 152 | 17.1 | 9.5 | 2.01 | 1 | 1 |
| P62195 | 26S proteasome regulatory subunit 8 OS=Homo sapiens OX=9606 GN=PSMC5 PE=1 SV=1 | 3 | 1 | 1 | 1 | 406 | 45.6 | 7.55 | 2.01 | 1 | 1 |
| O00425 | Insulin-like growth factor 2 mRNA-binding protein 3 OS=Homo sapiens OX=9606 GN=IGF2BP3 PE=1 SV=2 | 2 | 1 | 1 | 1 | 579 | 63.7 | 8.87 | 2 | 1 | 1 |
| Q5JTV8 | Torsin-1A-interacting protein 1 OS=Homo sapiens OX=9606 GN=TOR1AIP1 PE=1 SV=2 | 2 | 1 | 1 | 1 | 583 | 66.2 | 8.18 | 1.99 | 1 | 1 |
| Q15646 | 2'-5'-oligoadenylate synthase-like protein OS=Homo sapiens OX=9606 GN=OASL PE=1 SV=2 | 3 | 1 | 1 | 1 | 514 | 59.2 | 7.87 | 1.98 | 1 | 1 |
| Q3KR37 | Protein Aster-B OS=Homo sapiens OX=9606 GN=GRAMD1B PE=1 SV=1 | 1 | 1 | 1 | 1 | 738 | 85.3 | 6.21 | 1.98 | 1 | 1 |
| Q13576 | Ras GTPase-activating-like protein IQGAP2 OS=Homo sapiens OX=9606 GN=IQGAP2 PE=1 SV=4 | 1 | 1 | 1 | 1 | 1575 | 180.5 | 5.64 | 1.96 | 1 | 1 |
| Q01469 | Fatty acid-binding protein 5 OS=Homo sapiens OX=9606 GN=FABP5 PE=1 SV=3 | 7 | 1 | 1 | 1 | 135 | 15.2 | 7.01 | 1.95 | 1 | 1 |
| Q9UBS4 | DnaJ homolog subfamily B member 11 OS=Homo sapiens OX=9606 GN=DNAJB11 PE=1 SV=1 | 2 | 1 | 1 | 1 | 358 | 40.5 | 6.18 | 1.94 | 1 | 1 |
| Q8NDV3 | Structural maintenance of chromosomes protein 1B OS=Homo sapiens OX=9606 GN=SMC1B PE=2 SV=2 | 1 | 1 | 1 | 1 | 1235 | 143.8 | 7.66 | 1.94 | 1 | 1 |
| Q9Y4Z0 | U6 snRNA-associated Sm-like protein LSm4 OS=Homo sapiens OX=9606 GN=LSM4 PE=1 SV=1 | 6 | 1 | 1 | 1 | 139 | 15.3 | 9.99 | 1.92 | 1 | 1 |
| Q8N684 | Cleavage and polyadenylation specificity factor subunit 7 OS=Homo sapiens OX=9606 GN=CPSF7 PE=1 SV=1 | 3 | 1 | 1 | 1 | 471 | 52 | 8 | 1.92 | 1 | 1 |
| P20339 | Ras-related protein Rab-5A OS=Homo sapiens OX=9606 GN=RAB5A PE=1 SV=2 | 6 | 1 | 1 | 1 | 215 | 23.6 | 8.15 | 1.92 | 1 | 1 |
| Q6NUK1 | Calcium-binding mitochondrial carrier protein SCaMC-1 OS=Homo sapiens OX=9606 GN=SLC25A24 PE=1 SV=2 | 2 | 1 | 1 | 1 | 477 | 53.3 | 6.33 | 1.91 | 1 | 1 |
| Q9BYC9 | 39S ribosomal protein L20, mitochondrial OS=Homo sapiens OX=9606 GN=MRPL20 PE=1 SV=1 | 5 | 1 | 1 | 1 | 149 | 17.4 | 10.86 | 1.9 | 1 | 1 |
| P02042 | Hemoglobin subunit delta OS=Homo sapiens OX=9606 GN=HBD PE=1 SV=2 | 6 | 1 | 1 | 1 | 147 | 16 | 8.05 | 1.9 | 1 | 1 |
| Q8TDR0 | TRAF3-interacting protein 1 OS=Homo sapiens OX=9606 GN=TRAF3IP1 PE=1 SV=1 | 1 | 1 | 1 | 1 | 691 | 78.6 | 7.93 | 1.9 | 1 | 1 |

Table S2. The interacting protein of DDX3 identified by mass spectrometry.

| **Accession** | **Description** | **Coverage [%]** | **Peptides** | **PSMs** | **Unique Peptides** | **AAs** | **MW [kDa]** | **calc. pI** | **Score Sequest HT: Sequest HT** | **Peptides (by Search Engine): Sequest HT** | **Protein Groups** |
| --- | --- | --- | --- | --- | --- | --- | --- | --- | --- | --- | --- |
| O00571 | ATP-dependent RNA helicase DDX3X OS=Homo sapiens OX=9606 GN=DDX3X PE=1 SV=3 | 20 | 13 | 15 | 12 | 662 | 73.2 | 7.18 | 38.67 | 13 | 1 |
| Q14247 | Src substrate cortactin OS=Homo sapiens OX=9606 GN=CTTN PE=1 SV=2 | 41 | 21 | 34 | 21 | 550 | 61.5 | 5.4 | 85.25 | 21 | 1 |
| Q8WWM7 | Ataxin-2-like protein OS=Homo sapiens OX=9606 GN=ATXN2L PE=1 SV=2 | 17 | 15 | 24 | 15 | 1075 | 113.3 | 8.59 | 58.86 | 15 | 1 |
| P67809 | Y-box-binding protein 1 OS=Homo sapiens OX=9606 GN=YBX1 PE=1 SV=3 | 39 | 8 | 16 | 5 | 324 | 35.9 | 9.88 | 57.8 | 8 | 1 |
| P16403 | Histone H1.2 OS=Homo sapiens OX=9606 GN=H1-2 PE=1 SV=2 | 31 | 6 | 19 | 2 | 213 | 21.4 | 10.93 | 52.7 | 6 | 1 |
| O43852 | Calumenin OS=Homo sapiens OX=9606 GN=CALU PE=1 SV=2 | 33 | 10 | 20 | 10 | 315 | 37.1 | 4.64 | 52.05 | 10 | 1 |
| P22626 | Heterogeneous nuclear ribonucleoproteins A2/B1 OS=Homo sapiens OX=9606 GN=HNRNPA2B1 PE=1 SV=2 | 32 | 10 | 17 | 9 | 353 | 37.4 | 8.95 | 51.31 | 10 | 1 |
| P62424 | 60S ribosomal protein L7a OS=Homo sapiens OX=9606 GN=RPL7A PE=1 SV=2 | 30 | 9 | 18 | 9 | 266 | 30 | 10.61 | 50 | 9 | 1 |
| P60709 | Actin, cytoplasmic 1 OS=Homo sapiens OX=9606 GN=ACTB PE=1 SV=1 | 31 | 9 | 18 | 9 | 375 | 41.7 | 5.48 | 49.95 | 9 | 1 |
| P10412 | Histone H1.4 OS=Homo sapiens OX=9606 GN=H1-4 PE=1 SV=2 | 30 | 6 | 18 | 2 | 219 | 21.9 | 11.03 | 48.49 | 6 | 1 |
| P19338 | Nucleolin OS=Homo sapiens OX=9606 GN=NCL PE=1 SV=3 | 15 | 11 | 19 | 11 | 710 | 76.6 | 4.7 | 46.69 | 11 | 1 |
| P31327 | Carbamoyl-phosphate synthase [ammonia], mitochondrial OS=Homo sapiens OX=9606 GN=CPS1 PE=1 SV=2 | 12 | 16 | 18 | 16 | 1500 | 164.8 | 6.74 | 45.87 | 16 | 1 |
| P61978 | Heterogeneous nuclear ribonucleoprotein K OS=Homo sapiens OX=9606 GN=HNRNPK PE=1 SV=1 | 21 | 9 | 18 | 9 | 463 | 50.9 | 5.54 | 45.68 | 9 | 1 |
| Q07666 | KH domain-containing, RNA-binding, signal transduction-associated protein 1 OS=Homo sapiens OX=9606 GN=KHDRBS1 PE=1 SV=1 | 17 | 9 | 16 | 9 | 443 | 48.2 | 8.66 | 43.99 | 9 | 1 |
| P62750 | 60S ribosomal protein L23a OS=Homo sapiens OX=9606 GN=RPL23A PE=1 SV=1 | 38 | 7 | 15 | 7 | 156 | 17.7 | 10.45 | 43.17 | 7 | 1 |
| P16989 | Y-box-binding protein 3 OS=Homo sapiens OX=9606 GN=YBX3 PE=1 SV=4 | 39 | 8 | 14 | 5 | 372 | 40.1 | 9.77 | 42.75 | 8 | 1 |
| P35637 | RNA-binding protein FUS OS=Homo sapiens OX=9606 GN=FUS PE=1 SV=1 | 11 | 4 | 11 | 2 | 526 | 53.4 | 9.36 | 38.48 | 4 | 1 |
| P31942 | Heterogeneous nuclear ribonucleoprotein H3 OS=Homo sapiens OX=9606 GN=HNRNPH3 PE=1 SV=2 | 23 | 6 | 12 | 6 | 346 | 36.9 | 6.87 | 37.58 | 6 | 1 |
| P08238 | Heat shock protein HSP 90-beta OS=Homo sapiens OX=9606 GN=HSP90AB1 PE=1 SV=4 | 21 | 13 | 14 | 7 | 724 | 83.2 | 5.03 | 36.76 | 13 | 1 |
| P38646 | Stress-70 protein, mitochondrial OS=Homo sapiens OX=9606 GN=HSPA9 PE=1 SV=2 | 15 | 9 | 14 | 9 | 679 | 73.6 | 6.16 | 35.63 | 9 | 1 |
| Q86UE4 | Protein LYRIC OS=Homo sapiens OX=9606 GN=MTDH PE=1 SV=2 | 24 | 11 | 13 | 11 | 582 | 63.8 | 9.32 | 34.03 | 11 | 1 |
| P38159 | RNA-binding motif protein, X chromosome OS=Homo sapiens OX=9606 GN=RBMX PE=1 SV=3 | 23 | 9 | 14 | 9 | 391 | 42.3 | 10.05 | 33.88 | 9 | 1 |
| P11142 | Heat shock cognate 71 kDa protein OS=Homo sapiens OX=9606 GN=HSPA8 PE=1 SV=1 | 18 | 9 | 12 | 6 | 646 | 70.9 | 5.52 | 33.86 | 9 | 1 |
| P11021 | Endoplasmic reticulum chaperone BiP OS=Homo sapiens OX=9606 GN=HSPA5 PE=1 SV=2 | 17 | 9 | 12 | 7 | 654 | 72.3 | 5.16 | 32.57 | 9 | 1 |
| P0DMV9 | Heat shock 70 kDa protein 1B OS=Homo sapiens OX=9606 GN=HSPA1B PE=1 SV=1 | 14 | 7 | 12 | 5 | 641 | 70 | 5.66 | 32.39 | 7 | 1 |
| P14618 | Pyruvate kinase PKM OS=Homo sapiens OX=9606 GN=PKM PE=1 SV=4 | 19 | 9 | 12 | 9 | 531 | 57.9 | 7.84 | 32.3 | 9 | 1 |
| Q9BUJ2 | Heterogeneous nuclear ribonucleoprotein U-like protein 1 OS=Homo sapiens OX=9606 GN=HNRNPUL1 PE=1 SV=2 | 11 | 9 | 12 | 9 | 856 | 95.7 | 6.92 | 30.82 | 9 | 1 |
| P16401 | Histone H1.5 OS=Homo sapiens OX=9606 GN=H1-5 PE=1 SV=3 | 22 | 4 | 11 | 3 | 226 | 22.6 | 10.92 | 30.52 | 4 | 1 |
| P05388 | 60S acidic ribosomal protein P0 OS=Homo sapiens OX=9606 GN=RPLP0 PE=1 SV=1 | 24 | 7 | 11 | 7 | 317 | 34.3 | 5.97 | 29.91 | 7 | 1 |
| P29692 | Elongation factor 1-delta OS=Homo sapiens OX=9606 GN=EEF1D PE=1 SV=5 | 32 | 7 | 10 | 6 | 281 | 31.1 | 5.01 | 29.78 | 7 | 1 |
| P10809 | 60 kDa heat shock protein, mitochondrial OS=Homo sapiens OX=9606 GN=HSPD1 PE=1 SV=2 | 20 | 10 | 11 | 10 | 573 | 61 | 5.87 | 29.65 | 10 | 1 |
| Q92804 | TATA-binding protein-associated factor 2N OS=Homo sapiens OX=9606 GN=TAF15 PE=1 SV=1 | 14 | 5 | 11 | 3 | 592 | 61.8 | 8.02 | 29.34 | 5 | 1 |
| Q15293 | Reticulocalbin-1 OS=Homo sapiens OX=9606 GN=RCN1 PE=1 SV=1 | 14 | 4 | 10 | 4 | 331 | 38.9 | 5 | 28.65 | 4 | 1 |
| P11940 | Polyadenylate-binding protein 1 OS=Homo sapiens OX=9606 GN=PABPC1 PE=1 SV=2 | 14 | 8 | 11 | 5 | 636 | 70.6 | 9.5 | 28.52 | 8 | 1 |
| Q12906 | Interleukin enhancer-binding factor 3 OS=Homo sapiens OX=9606 GN=ILF3 PE=1 SV=3 | 13 | 10 | 12 | 10 | 894 | 95.3 | 8.76 | 27.74 | 10 | 1 |
| P07900 | Heat shock protein HSP 90-alpha OS=Homo sapiens OX=9606 GN=HSP90AA1 PE=1 SV=5 | 14 | 9 | 11 | 3 | 732 | 84.6 | 5.02 | 26.83 | 9 | 1 |
| P62269 | 40S ribosomal protein S18 OS=Homo sapiens OX=9606 GN=RPS18 PE=1 SV=3 | 37 | 6 | 10 | 6 | 152 | 17.7 | 10.99 | 26.64 | 6 | 1 |
| P17096 | High mobility group protein HMG-I/HMG-Y OS=Homo sapiens OX=9606 GN=HMGA1 PE=1 SV=3 | 37 | 4 | 10 | 4 | 107 | 11.7 | 10.32 | 26.48 | 4 | 1 |
| P61254 | 60S ribosomal protein L26 OS=Homo sapiens OX=9606 GN=RPL26 PE=1 SV=1 | 34 | 7 | 10 | 2 | 145 | 17.2 | 10.55 | 25.61 | 7 | 1 |
| P49411 | Elongation factor Tu, mitochondrial OS=Homo sapiens OX=9606 GN=TUFM PE=1 SV=2 | 13 | 6 | 11 | 6 | 452 | 49.5 | 7.61 | 25.54 | 6 | 1 |
| P23396 | 40S ribosomal protein S3 OS=Homo sapiens OX=9606 GN=RPS3 PE=1 SV=2 | 35 | 7 | 10 | 7 | 243 | 26.7 | 9.66 | 25.22 | 7 | 1 |
| P26373 | 60S ribosomal protein L13 OS=Homo sapiens OX=9606 GN=RPL13 PE=1 SV=4 | 29 | 6 | 9 | 6 | 211 | 24.2 | 11.65 | 24.99 | 6 | 1 |
| P62917 | 60S ribosomal protein L8 OS=Homo sapiens OX=9606 GN=RPL8 PE=1 SV=2 | 19 | 5 | 9 | 5 | 257 | 28 | 11.03 | 24.49 | 5 | 1 |
| P62851 | 40S ribosomal protein S25 OS=Homo sapiens OX=9606 GN=RPS25 PE=1 SV=1 | 30 | 5 | 9 | 5 | 125 | 13.7 | 10.11 | 24.22 | 5 | 1 |
| P46781 | 40S ribosomal protein S9 OS=Homo sapiens OX=9606 GN=RPS9 PE=1 SV=3 | 36 | 8 | 11 | 8 | 194 | 22.6 | 10.65 | 24.02 | 8 | 1 |
| P51991 | Heterogeneous nuclear ribonucleoprotein A3 OS=Homo sapiens OX=9606 GN=HNRNPA3 PE=1 SV=2 | 25 | 7 | 9 | 7 | 378 | 39.6 | 9.01 | 23.76 | 7 | 1 |
| P05387 | 60S acidic ribosomal protein P2 OS=Homo sapiens OX=9606 GN=RPLP2 PE=1 SV=1 | 57 | 4 | 8 | 4 | 115 | 11.7 | 4.54 | 23.13 | 4 | 1 |
| Q02878 | 60S ribosomal protein L6 OS=Homo sapiens OX=9606 GN=RPL6 PE=1 SV=3 | 19 | 7 | 9 | 7 | 288 | 32.7 | 10.58 | 23 | 7 | 1 |
| P18621 | 60S ribosomal protein L17 OS=Homo sapiens OX=9606 GN=RPL17 PE=1 SV=3 | 18 | 3 | 8 | 3 | 184 | 21.4 | 10.17 | 22.41 | 3 | 1 |
| Q86V81 | THO complex subunit 4 OS=Homo sapiens OX=9606 GN=ALYREF PE=1 SV=3 | 26 | 5 | 8 | 5 | 257 | 26.9 | 11.15 | 22.35 | 5 | 1 |
| Q13310 | Polyadenylate-binding protein 4 OS=Homo sapiens OX=9606 GN=PABPC4 PE=1 SV=1 | 13 | 7 | 9 | 4 | 644 | 70.7 | 9.26 | 22.32 | 7 | 1 |
| O43390 | Heterogeneous nuclear ribonucleoprotein R OS=Homo sapiens OX=9606 GN=HNRNPR PE=1 SV=1 | 13 | 8 | 9 | 4 | 633 | 70.9 | 8.13 | 22.27 | 8 | 1 |
| P62937 | Peptidyl-prolyl cis-trans isomerase A OS=Homo sapiens OX=9606 GN=PPIA PE=1 SV=2 | 26 | 4 | 9 | 4 | 165 | 18 | 7.81 | 22.26 | 4 | 1 |
| Q9UNX3 | 60S ribosomal protein L26-like 1 OS=Homo sapiens OX=9606 GN=RPL26L1 PE=1 SV=1 | 28 | 6 | 9 | 1 | 145 | 17.2 | 10.55 | 22.21 | 6 | 1 |
| Q92522 | Histone H1.10 OS=Homo sapiens OX=9606 GN=H1-10 PE=1 SV=1 | 28 | 6 | 8 | 6 | 213 | 22.5 | 10.76 | 21.67 | 6 | 1 |
| P07910 | Heterogeneous nuclear ribonucleoproteins C1/C2 OS=Homo sapiens OX=9606 GN=HNRNPC PE=1 SV=4 | 24 | 8 | 9 | 8 | 306 | 33.7 | 5.08 | 21.61 | 8 | 1 |
| P07437 | Tubulin beta chain OS=Homo sapiens OX=9606 GN=TUBB PE=1 SV=2 | 12 | 5 | 9 | 1 | 444 | 49.6 | 4.89 | 21.51 | 5 | 1 |
| P68371 | Tubulin beta-4B chain OS=Homo sapiens OX=9606 GN=TUBB4B PE=1 SV=1 | 12 | 5 | 9 | 1 | 445 | 49.8 | 4.89 | 21.33 | 5 | 1 |
| P15121 | Aldo-keto reductase family 1 member B1 OS=Homo sapiens OX=9606 GN=AKR1B1 PE=1 SV=3 | 14 | 5 | 9 | 5 | 316 | 35.8 | 6.98 | 21.28 | 5 | 1 |
| P68104 | Elongation factor 1-alpha 1 OS=Homo sapiens OX=9606 GN=EEF1A1 PE=1 SV=1 | 12 | 6 | 8 | 6 | 462 | 50.1 | 9.01 | 20.32 | 6 | 1 |
| P00338 | L-lactate dehydrogenase A chain OS=Homo sapiens OX=9606 GN=LDHA PE=1 SV=2 | 20 | 6 | 8 | 5 | 332 | 36.7 | 8.27 | 20.26 | 6 | 1 |
| P06748 | Nucleophosmin OS=Homo sapiens OX=9606 GN=NPM1 PE=1 SV=2 | 22 | 6 | 9 | 6 | 294 | 32.6 | 4.78 | 19.68 | 6 | 1 |
| O60506 | Heterogeneous nuclear ribonucleoprotein Q OS=Homo sapiens OX=9606 GN=SYNCRIP PE=1 SV=2 | 11 | 7 | 8 | 3 | 623 | 69.6 | 8.59 | 19.37 | 7 | 1 |
| Q07955 | Serine/arginine-rich splicing factor 1 OS=Homo sapiens OX=9606 GN=SRSF1 PE=1 SV=2 | 23 | 6 | 8 | 6 | 248 | 27.7 | 10.36 | 19.09 | 6 | 1 |
| P60842 | Eukaryotic initiation factor 4A-I OS=Homo sapiens OX=9606 GN=EIF4A1 PE=1 SV=1 | 22 | 8 | 8 | 7 | 406 | 46.1 | 5.48 | 18.12 | 8 | 1 |
| P06733 | Alpha-enolase OS=Homo sapiens OX=9606 GN=ENO1 PE=1 SV=2 | 13 | 6 | 7 | 6 | 434 | 47.1 | 7.39 | 17.7 | 6 | 1 |
| P62899 | 60S ribosomal protein L31 OS=Homo sapiens OX=9606 GN=RPL31 PE=1 SV=1 | 26 | 4 | 8 | 4 | 125 | 14.5 | 10.54 | 17.63 | 4 | 1 |
| Q99729 | Heterogeneous nuclear ribonucleoprotein A/B OS=Homo sapiens OX=9606 GN=HNRNPAB PE=1 SV=2 | 13 | 5 | 7 | 4 | 332 | 36.2 | 8.21 | 17.15 | 5 | 1 |
| P31943 | Heterogeneous nuclear ribonucleoprotein H OS=Homo sapiens OX=9606 GN=HNRNPH1 PE=1 SV=4 | 10 | 4 | 6 | 4 | 449 | 49.2 | 6.3 | 16.77 | 4 | 1 |
| P39019 | 40S ribosomal protein S19 OS=Homo sapiens OX=9606 GN=RPS19 PE=1 SV=2 | 45 | 7 | 7 | 7 | 145 | 16.1 | 10.32 | 16.62 | 7 | 1 |
| Q00839 | Heterogeneous nuclear ribonucleoprotein U OS=Homo sapiens OX=9606 GN=HNRNPU PE=1 SV=6 | 10 | 6 | 7 | 6 | 825 | 90.5 | 6 | 16.62 | 6 | 1 |
| Q9Y3I0 | RNA-splicing ligase RtcB homolog OS=Homo sapiens OX=9606 GN=RTCB PE=1 SV=1 | 10 | 5 | 7 | 5 | 505 | 55.2 | 7.23 | 16.33 | 5 | 1 |
| P04406 | Glyceraldehyde-3-phosphate dehydrogenase OS=Homo sapiens OX=9606 GN=GAPDH PE=1 SV=3 | 16 | 5 | 6 | 5 | 335 | 36 | 8.46 | 16.13 | 5 | 1 |
| P05023 | Sodium/potassium-transporting ATPase subunit alpha-1 OS=Homo sapiens OX=9606 GN=ATP1A1 PE=1 SV=1 | 9 | 6 | 6 | 6 | 1023 | 112.8 | 5.49 | 15.53 | 6 | 1 |
| Q71U36 | Tubulin alpha-1A chain OS=Homo sapiens OX=9606 GN=TUBA1A PE=1 SV=1 | 16 | 6 | 6 | 6 | 451 | 50.1 | 5.06 | 15.51 | 6 | 1 |
| P62826 | GTP-binding nuclear protein Ran OS=Homo sapiens OX=9606 GN=RAN PE=1 SV=3 | 24 | 5 | 6 | 5 | 216 | 24.4 | 7.49 | 15.2 | 5 | 1 |
| Q01130 | Serine/arginine-rich splicing factor 2 OS=Homo sapiens OX=9606 GN=SRSF2 PE=1 SV=4 | 19 | 3 | 6 | 3 | 221 | 25.5 | 11.85 | 15.2 | 3 | 1 |
| P17844 | Probable ATP-dependent RNA helicase DDX5 OS=Homo sapiens OX=9606 GN=DDX5 PE=1 SV=1 | 7 | 4 | 6 | 3 | 614 | 69.1 | 8.92 | 15.11 | 4 | 1 |
| P62753 | 40S ribosomal protein S6 OS=Homo sapiens OX=9606 GN=RPS6 PE=1 SV=1 | 21 | 5 | 6 | 5 | 249 | 28.7 | 10.84 | 14.94 | 5 | 1 |
| P51398 | 28S ribosomal protein S29, mitochondrial OS=Homo sapiens OX=9606 GN=DAP3 PE=1 SV=1 | 13 | 4 | 6 | 4 | 398 | 45.5 | 8.88 | 14.63 | 4 | 1 |
| P61353 | 60S ribosomal protein L27 OS=Homo sapiens OX=9606 GN=RPL27 PE=1 SV=2 | 49 | 6 | 6 | 6 | 136 | 15.8 | 10.56 | 14.29 | 6 | 1 |
| P81605 | Dermcidin OS=Homo sapiens OX=9606 GN=DCD PE=1 SV=2 | 27 | 3 | 6 | 3 | 110 | 11.3 | 6.54 | 13.95 | 3 | 1 |
| P25705 | ATP synthase subunit alpha, mitochondrial OS=Homo sapiens OX=9606 GN=ATP5F1A PE=1 SV=1 | 12 | 6 | 6 | 6 | 553 | 59.7 | 9.13 | 13.91 | 6 | 1 |
| P36578 | 60S ribosomal protein L4 OS=Homo sapiens OX=9606 GN=RPL4 PE=1 SV=5 | 8 | 3 | 5 | 3 | 427 | 47.7 | 11.06 | 13.8 | 3 | 1 |
| O14979 | Heterogeneous nuclear ribonucleoprotein D-like OS=Homo sapiens OX=9606 GN=HNRNPDL PE=1 SV=3 | 11 | 4 | 5 | 3 | 420 | 46.4 | 9.57 | 13.34 | 4 | 1 |
| P30050 | 60S ribosomal protein L12 OS=Homo sapiens OX=9606 GN=RPL12 PE=1 SV=1 | 19 | 3 | 5 | 3 | 165 | 17.8 | 9.42 | 13.31 | 3 | 1 |
| P00558 | Phosphoglycerate kinase 1 OS=Homo sapiens OX=9606 GN=PGK1 PE=1 SV=3 | 14 | 5 | 5 | 5 | 417 | 44.6 | 8.1 | 13.29 | 5 | 1 |
| P62266 | 40S ribosomal protein S23 OS=Homo sapiens OX=9606 GN=RPS23 PE=1 SV=3 | 21 | 3 | 5 | 3 | 143 | 15.8 | 10.49 | 12.98 | 3 | 1 |
| Q96HS1 | Serine/threonine-protein phosphatase PGAM5, mitochondrial OS=Homo sapiens OX=9606 GN=PGAM5 PE=1 SV=2 | 15 | 4 | 6 | 4 | 289 | 32 | 8.68 | 12.97 | 4 | 1 |
| Q14444 | Caprin-1 OS=Homo sapiens OX=9606 GN=CAPRIN1 PE=1 SV=2 | 13 | 5 | 5 | 5 | 709 | 78.3 | 5.25 | 12.93 | 5 | 1 |
| P46778 | 60S ribosomal protein L21 OS=Homo sapiens OX=9606 GN=RPL21 PE=1 SV=2 | 16 | 3 | 5 | 3 | 160 | 18.6 | 10.49 | 12.83 | 3 | 1 |
| Q86YZ3 | Hornerin OS=Homo sapiens OX=9606 GN=HRNR PE=1 SV=2 | 8 | 4 | 4 | 4 | 2850 | 282.2 | 10.04 | 12.64 | 4 | 1 |
| P84098 | 60S ribosomal protein L19 OS=Homo sapiens OX=9606 GN=RPL19 PE=1 SV=1 | 16 | 3 | 5 | 2 | 196 | 23.5 | 11.47 | 12.53 | 3 | 1 |
| P62263 | 40S ribosomal protein S14 OS=Homo sapiens OX=9606 GN=RPS14 PE=1 SV=3 | 28 | 3 | 4 | 3 | 151 | 16.3 | 10.05 | 12.47 | 3 | 1 |
| Q9BRJ6 | Uncharacterized protein C7orf50 OS=Homo sapiens OX=9606 GN=C7orf50 PE=1 SV=1 | 28 | 3 | 4 | 3 | 194 | 22.1 | 9.64 | 12.22 | 3 | 1 |
| P08670 | Vimentin OS=Homo sapiens OX=9606 GN=VIM PE=1 SV=4 | 10 | 5 | 5 | 4 | 466 | 53.6 | 5.12 | 12 | 5 | 1 |
| Q9Y3U8 | 60S ribosomal protein L36 OS=Homo sapiens OX=9606 GN=RPL36 PE=1 SV=3 | 30 | 4 | 5 | 4 | 105 | 12.2 | 11.59 | 11.9 | 4 | 1 |
| O14950 | Myosin regulatory light chain 12B OS=Homo sapiens OX=9606 GN=MYL12B PE=1 SV=2 | 24 | 4 | 5 | 4 | 172 | 19.8 | 4.84 | 11.73 | 4 | 1 |
| P62841 | 40S ribosomal protein S15 OS=Homo sapiens OX=9606 GN=RPS15 PE=1 SV=2 | 9 | 2 | 4 | 2 | 145 | 17 | 10.39 | 11.65 | 2 | 1 |
| Q99848 | Probable rRNA-processing protein EBP2 OS=Homo sapiens OX=9606 GN=EBNA1BP2 PE=1 SV=2 | 14 | 4 | 5 | 4 | 306 | 34.8 | 10.1 | 11.64 | 4 | 1 |
| P07305 | Histone H1.0 OS=Homo sapiens OX=9606 GN=H1-0 PE=1 SV=3 | 21 | 4 | 5 | 4 | 194 | 20.9 | 10.84 | 11.6 | 4 | 1 |
| P82650 | 28S ribosomal protein S22, mitochondrial OS=Homo sapiens OX=9606 GN=MRPS22 PE=1 SV=1 | 13 | 4 | 4 | 4 | 360 | 41.3 | 7.9 | 11.42 | 4 | 1 |
| P07737 | Profilin-1 OS=Homo sapiens OX=9606 GN=PFN1 PE=1 SV=2 | 37 | 4 | 4 | 4 | 140 | 15 | 8.27 | 11.32 | 4 | 1 |
| P84090 | Enhancer of rudimentary homolog OS=Homo sapiens OX=9606 GN=ERH PE=1 SV=1 | 12 | 1 | 3 | 1 | 104 | 12.3 | 5.92 | 11.23 | 1 | 1 |
| Q9BXS6 | Nucleolar and spindle-associated protein 1 OS=Homo sapiens OX=9606 GN=NUSAP1 PE=1 SV=1 | 12 | 4 | 5 | 4 | 441 | 49.4 | 9.91 | 11.21 | 4 | 1 |
| P18077 | 60S ribosomal protein L35a OS=Homo sapiens OX=9606 GN=RPL35A PE=1 SV=2 | 40 | 5 | 5 | 5 | 110 | 12.5 | 11.06 | 11.14 | 5 | 1 |
| Q13247 | Serine/arginine-rich splicing factor 6 OS=Homo sapiens OX=9606 GN=SRSF6 PE=1 SV=2 | 9 | 3 | 5 | 3 | 344 | 39.6 | 11.43 | 11.14 | 3 | 1 |
| Q9Y224 | RNA transcription, translation and transport factor protein OS=Homo sapiens OX=9606 GN=RTRAF PE=1 SV=1 | 14 | 4 | 5 | 4 | 244 | 28.1 | 6.65 | 11.07 | 4 | 1 |
| O75821 | Eukaryotic translation initiation factor 3 subunit G OS=Homo sapiens OX=9606 GN=EIF3G PE=1 SV=2 | 11 | 3 | 4 | 3 | 320 | 35.6 | 6.13 | 11.06 | 3 | 1 |
| Q06830 | Peroxiredoxin-1 OS=Homo sapiens OX=9606 GN=PRDX1 PE=1 SV=1 | 24 | 5 | 5 | 5 | 199 | 22.1 | 8.13 | 10.92 | 5 | 1 |
| P09651 | Heterogeneous nuclear ribonucleoprotein A1 OS=Homo sapiens OX=9606 GN=HNRNPA1 PE=1 SV=5 | 11 | 3 | 4 | 2 | 372 | 38.7 | 9.13 | 10.92 | 3 | 1 |
| P35268 | 60S ribosomal protein L22 OS=Homo sapiens OX=9606 GN=RPL22 PE=1 SV=2 | 19 | 2 | 4 | 2 | 128 | 14.8 | 9.19 | 10.84 | 2 | 1 |
| Q15365 | Poly(rC)-binding protein 1 OS=Homo sapiens OX=9606 GN=PCBP1 PE=1 SV=2 | 9 | 3 | 4 | 2 | 356 | 37.5 | 7.09 | 10.67 | 3 | 1 |
| P50990 | T-complex protein 1 subunit theta OS=Homo sapiens OX=9606 GN=CCT8 PE=1 SV=4 | 8 | 4 | 4 | 4 | 548 | 59.6 | 5.6 | 10.65 | 4 | 1 |
| P60866 | 40S ribosomal protein S20 OS=Homo sapiens OX=9606 GN=RPS20 PE=1 SV=1 | 25 | 3 | 4 | 3 | 119 | 13.4 | 9.94 | 10.6 | 3 | 1 |
| P05141 | ADP/ATP translocase 2 OS=Homo sapiens OX=9606 GN=SLC25A5 PE=1 SV=7 | 10 | 3 | 4 | 3 | 298 | 32.8 | 9.69 | 10.54 | 3 | 1 |
| P15880 | 40S ribosomal protein S2 OS=Homo sapiens OX=9606 GN=RPS2 PE=1 SV=2 | 10 | 3 | 4 | 3 | 293 | 31.3 | 10.24 | 10.45 | 3 | 1 |
| O43809 | Cleavage and polyadenylation specificity factor subunit 5 OS=Homo sapiens OX=9606 GN=NUDT21 PE=1 SV=1 | 20 | 4 | 4 | 4 | 227 | 26.2 | 8.82 | 10.34 | 4 | 1 |
| P47914 | 60S ribosomal protein L29 OS=Homo sapiens OX=9606 GN=RPL29 PE=1 SV=2 | 14 | 2 | 3 | 2 | 159 | 17.7 | 11.66 | 10.03 | 2 | 1 |
| Q15366 | Poly(rC)-binding protein 2 OS=Homo sapiens OX=9606 GN=PCBP2 PE=1 SV=1 | 12 | 3 | 4 | 2 | 365 | 38.6 | 6.79 | 9.92 | 3 | 1 |
| P46783 | 40S ribosomal protein S10 OS=Homo sapiens OX=9606 GN=RPS10 PE=1 SV=1 | 13 | 2 | 3 | 2 | 165 | 18.9 | 10.15 | 9.88 | 2 | 1 |
| P02786 | Transferrin receptor protein 1 OS=Homo sapiens OX=9606 GN=TFRC PE=1 SV=2 | 6 | 4 | 4 | 4 | 760 | 84.8 | 6.61 | 9.71 | 4 | 1 |
| P07355 | Annexin A2 OS=Homo sapiens OX=9606 GN=ANXA2 PE=1 SV=2 | 14 | 4 | 4 | 4 | 339 | 38.6 | 7.75 | 9.68 | 4 | 1 |
| O94925 | Glutaminase kidney isoform, mitochondrial OS=Homo sapiens OX=9606 GN=GLS PE=1 SV=1 | 8 | 3 | 3 | 3 | 669 | 73.4 | 7.77 | 9.68 | 3 | 1 |
| P42766 | 60S ribosomal protein L35 OS=Homo sapiens OX=9606 GN=RPL35 PE=1 SV=2 | 14 | 3 | 4 | 2 | 123 | 14.5 | 11.05 | 9.64 | 3 | 1 |
| Q01844 | RNA-binding protein EWS OS=Homo sapiens OX=9606 GN=EWSR1 PE=1 SV=1 | 4 | 2 | 4 | 2 | 656 | 68.4 | 9.33 | 9.53 | 2 | 1 |
| P05198 | Eukaryotic translation initiation factor 2 subunit 1 OS=Homo sapiens OX=9606 GN=EIF2S1 PE=1 SV=3 | 10 | 3 | 4 | 3 | 315 | 36.1 | 5.08 | 9.48 | 3 | 1 |
| P62277 | 40S ribosomal protein S13 OS=Homo sapiens OX=9606 GN=RPS13 PE=1 SV=2 | 17 | 3 | 4 | 3 | 151 | 17.2 | 10.54 | 9.47 | 3 | 1 |
| P83731 | 60S ribosomal protein L24 OS=Homo sapiens OX=9606 GN=RPL24 PE=1 SV=1 | 14 | 2 | 3 | 2 | 157 | 17.8 | 11.25 | 9.41 | 2 | 1 |
| P46779 | 60S ribosomal protein L28 OS=Homo sapiens OX=9606 GN=RPL28 PE=1 SV=3 | 27 | 4 | 4 | 4 | 137 | 15.7 | 12.02 | 9.32 | 4 | 1 |
| O95429 | BAG family molecular chaperone regulator 4 OS=Homo sapiens OX=9606 GN=BAG4 PE=1 SV=1 | 6 | 2 | 3 | 2 | 457 | 49.6 | 5.12 | 9.31 | 2 | 1 |
| P60660 | Myosin light polypeptide 6 OS=Homo sapiens OX=9606 GN=MYL6 PE=1 SV=2 | 28 | 4 | 4 | 4 | 151 | 16.9 | 4.65 | 9.25 | 4 | 1 |
| Q9BXP5 | Serrate RNA effector molecule homolog OS=Homo sapiens OX=9606 GN=SRRT PE=1 SV=1 | 6 | 4 | 4 | 4 | 876 | 100.6 | 5.96 | 9.23 | 4 | 1 |
| P62280 | 40S ribosomal protein S11 OS=Homo sapiens OX=9606 GN=RPS11 PE=1 SV=3 | 11 | 2 | 4 | 2 | 158 | 18.4 | 10.3 | 9.23 | 2 | 1 |
| P08865 | 40S ribosomal protein SA OS=Homo sapiens OX=9606 GN=RPSA PE=1 SV=4 | 10 | 2 | 3 | 2 | 295 | 32.8 | 4.87 | 9.14 | 2 | 1 |
| P40939 | Trifunctional enzyme subunit alpha, mitochondrial OS=Homo sapiens OX=9606 GN=HADHA PE=1 SV=2 | 5 | 3 | 4 | 3 | 763 | 82.9 | 9.04 | 8.94 | 3 | 1 |
| P62244 | 40S ribosomal protein S15a OS=Homo sapiens OX=9606 GN=RPS15A PE=1 SV=2 | 25 | 3 | 4 | 3 | 130 | 14.8 | 10.13 | 8.83 | 3 | 1 |
| Q13435 | Splicing factor 3B subunit 2 OS=Homo sapiens OX=9606 GN=SF3B2 PE=1 SV=2 | 4 | 3 | 4 | 3 | 895 | 100.2 | 5.67 | 8.79 | 3 | 1 |
| Q71UI9 | Histone H2A.V OS=Homo sapiens OX=9606 GN=H2AZ2 PE=1 SV=3 | 20 | 3 | 4 | 3 | 128 | 13.5 | 10.58 | 8.62 | 3 | 1 |
| P40429 | 60S ribosomal protein L13a OS=Homo sapiens OX=9606 GN=RPL13A PE=1 SV=2 | 14 | 3 | 4 | 3 | 203 | 23.6 | 10.93 | 8.43 | 3 | 1 |
| P62910 | 60S ribosomal protein L32 OS=Homo sapiens OX=9606 GN=RPL32 PE=1 SV=2 | 26 | 4 | 4 | 4 | 135 | 15.9 | 11.33 | 8.41 | 4 | 1 |
| P18124 | 60S ribosomal protein L7 OS=Homo sapiens OX=9606 GN=RPL7 PE=1 SV=1 | 17 | 4 | 4 | 4 | 248 | 29.2 | 10.65 | 8.26 | 4 | 1 |
| Q7Z417 | Nuclear fragile X mental retardation-interacting protein 2 OS=Homo sapiens OX=9606 GN=NUFIP2 PE=1 SV=1 | 3 | 2 | 3 | 2 | 695 | 76.1 | 8.7 | 8.22 | 2 | 1 |
| Q14103 | Heterogeneous nuclear ribonucleoprotein D0 OS=Homo sapiens OX=9606 GN=HNRNPD PE=1 SV=1 | 10 | 3 | 3 | 2 | 355 | 38.4 | 7.81 | 8.07 | 3 | 1 |
| P62318 | Small nuclear ribonucleoprotein Sm D3 OS=Homo sapiens OX=9606 GN=SNRPD3 PE=1 SV=1 | 25 | 2 | 3 | 2 | 126 | 13.9 | 10.32 | 7.9 | 2 | 1 |
| P02768 | Albumin OS=Homo sapiens OX=9606 GN=ALB PE=1 SV=2 | 4 | 2 | 3 | 2 | 609 | 69.3 | 6.28 | 7.79 | 2 | 1 |
| Q9P0W8 | Spermatogenesis-associated protein 7 OS=Homo sapiens OX=9606 GN=SPATA7 PE=1 SV=3 | 1 | 1 | 4 | 1 | 599 | 67.7 | 6.29 | 7.71 | 1 | 1 |
| P62987 | Ubiquitin-60S ribosomal protein L40 OS=Homo sapiens OX=9606 GN=UBA52 PE=1 SV=2 | 20 | 2 | 3 | 2 | 128 | 14.7 | 9.83 | 7.66 | 2 | 1 |
| P24534 | Elongation factor 1-beta OS=Homo sapiens OX=9606 GN=EEF1B2 PE=1 SV=3 | 17 | 3 | 3 | 2 | 225 | 24.7 | 4.67 | 7.65 | 3 | 1 |
| P04075 | Fructose-bisphosphate aldolase A OS=Homo sapiens OX=9606 GN=ALDOA PE=1 SV=2 | 14 | 3 | 3 | 3 | 364 | 39.4 | 8.09 | 7.48 | 3 | 1 |
| P82933 | 28S ribosomal protein S9, mitochondrial OS=Homo sapiens OX=9606 GN=MRPS9 PE=1 SV=2 | 6 | 2 | 3 | 2 | 396 | 45.8 | 9.51 | 7.47 | 2 | 1 |
| P27824 | Calnexin OS=Homo sapiens OX=9606 GN=CANX PE=1 SV=2 | 5 | 3 | 3 | 3 | 592 | 67.5 | 4.6 | 7.44 | 3 | 1 |
| P62316 | Small nuclear ribonucleoprotein Sm D2 OS=Homo sapiens OX=9606 GN=SNRPD2 PE=1 SV=1 | 16 | 2 | 3 | 2 | 118 | 13.5 | 9.91 | 7.4 | 2 | 1 |
| Q9Y295 | Developmentally-regulated GTP-binding protein 1 OS=Homo sapiens OX=9606 GN=DRG1 PE=1 SV=1 | 11 | 3 | 3 | 3 | 367 | 40.5 | 8.9 | 7.36 | 3 | 1 |
| Q969Q0 | 60S ribosomal protein L36a-like OS=Homo sapiens OX=9606 GN=RPL36AL PE=1 SV=3 | 16 | 3 | 3 | 3 | 106 | 12.5 | 10.65 | 7.35 | 3 | 1 |
| Q07020 | 60S ribosomal protein L18 OS=Homo sapiens OX=9606 GN=RPL18 PE=1 SV=2 | 18 | 3 | 3 | 3 | 188 | 21.6 | 11.72 | 7.34 | 3 | 1 |
| P12956 | X-ray repair cross-complementing protein 6 OS=Homo sapiens OX=9606 GN=XRCC6 PE=1 SV=2 | 5 | 3 | 3 | 3 | 609 | 69.8 | 6.64 | 7.32 | 3 | 1 |
| Q15084 | Protein disulfide-isomerase A6 OS=Homo sapiens OX=9606 GN=PDIA6 PE=1 SV=1 | 7 | 2 | 3 | 2 | 440 | 48.1 | 5.08 | 7.21 | 2 | 1 |
| P07195 | L-lactate dehydrogenase B chain OS=Homo sapiens OX=9606 GN=LDHB PE=1 SV=2 | 10 | 3 | 3 | 2 | 334 | 36.6 | 6.05 | 7.18 | 3 | 1 |
| Q9UHB6 | LIM domain and actin-binding protein 1 OS=Homo sapiens OX=9606 GN=LIMA1 PE=1 SV=1 | 3 | 2 | 3 | 2 | 759 | 85.2 | 6.84 | 7.18 | 2 | 1 |
| P06576 | ATP synthase subunit beta, mitochondrial OS=Homo sapiens OX=9606 GN=ATP5F1B PE=1 SV=3 | 7 | 3 | 3 | 3 | 529 | 56.5 | 5.4 | 7.13 | 3 | 1 |
| P21333 | Filamin-A OS=Homo sapiens OX=9606 GN=FLNA PE=1 SV=4 | 1 | 2 | 3 | 2 | 2647 | 280.6 | 6.06 | 7.13 | 2 | 1 |
| Q9NX63 | MICOS complex subunit MIC19 OS=Homo sapiens OX=9606 GN=CHCHD3 PE=1 SV=1 | 9 | 2 | 3 | 2 | 227 | 26.1 | 8.28 | 7.08 | 2 | 1 |
| Q9Y3C1 | Nucleolar protein 16 OS=Homo sapiens OX=9606 GN=NOP16 PE=1 SV=2 | 24 | 3 | 3 | 3 | 178 | 21.2 | 9.94 | 7.05 | 3 | 1 |
| P61247 | 40S ribosomal protein S3a OS=Homo sapiens OX=9606 GN=RPS3A PE=1 SV=2 | 11 | 3 | 3 | 3 | 264 | 29.9 | 9.73 | 6.96 | 3 | 1 |
| Q09028 | Histone-binding protein RBBP4 OS=Homo sapiens OX=9606 GN=RBBP4 PE=1 SV=3 | 6 | 2 | 2 | 2 | 425 | 47.6 | 4.89 | 6.92 | 2 | 1 |
| Q92499 | ATP-dependent RNA helicase DDX1 OS=Homo sapiens OX=9606 GN=DDX1 PE=1 SV=2 | 4 | 3 | 3 | 3 | 740 | 82.4 | 7.23 | 6.87 | 3 | 1 |
| P23921 | Ribonucleoside-diphosphate reductase large subunit OS=Homo sapiens OX=9606 GN=RRM1 PE=1 SV=1 | 3 | 2 | 3 | 2 | 792 | 90 | 7.15 | 6.85 | 2 | 1 |
| Q16629 | Serine/arginine-rich splicing factor 7 OS=Homo sapiens OX=9606 GN=SRSF7 PE=1 SV=1 | 12 | 3 | 3 | 3 | 238 | 27.4 | 11.82 | 6.83 | 3 | 1 |
| P23246 | Splicing factor, proline- and glutamine-rich OS=Homo sapiens OX=9606 GN=SFPQ PE=1 SV=2 | 3 | 2 | 3 | 2 | 707 | 76.1 | 9.44 | 6.83 | 2 | 1 |
| P04792 | Heat shock protein beta-1 OS=Homo sapiens OX=9606 GN=HSPB1 PE=1 SV=2 | 13 | 2 | 3 | 2 | 205 | 22.8 | 6.4 | 6.76 | 2 | 1 |
| P20042 | Eukaryotic translation initiation factor 2 subunit 2 OS=Homo sapiens OX=9606 GN=EIF2S2 PE=1 SV=2 | 10 | 3 | 3 | 3 | 333 | 38.4 | 5.8 | 6.75 | 3 | 1 |
| P55084 | Trifunctional enzyme subunit beta, mitochondrial OS=Homo sapiens OX=9606 GN=HADHB PE=1 SV=3 | 4 | 2 | 3 | 2 | 474 | 51.3 | 9.41 | 6.74 | 2 | 1 |
| P84103 | Serine/arginine-rich splicing factor 3 OS=Homo sapiens OX=9606 GN=SRSF3 PE=1 SV=1 | 12 | 2 | 3 | 2 | 164 | 19.3 | 11.65 | 6.73 | 2 | 1 |
| Q13263 | Transcription intermediary factor 1-beta OS=Homo sapiens OX=9606 GN=TRIM28 PE=1 SV=5 | 6 | 3 | 3 | 3 | 835 | 88.5 | 5.77 | 6.71 | 3 | 1 |
| Q9UN86 | Ras GTPase-activating protein-binding protein 2 OS=Homo sapiens OX=9606 GN=G3BP2 PE=1 SV=2 | 5 | 2 | 2 | 2 | 482 | 54.1 | 5.55 | 6.6 | 2 | 1 |
| P14866 | Heterogeneous nuclear ribonucleoprotein L OS=Homo sapiens OX=9606 GN=HNRNPL PE=1 SV=2 | 6 | 3 | 3 | 3 | 589 | 64.1 | 8.22 | 6.56 | 3 | 1 |
| O75396 | Vesicle-trafficking protein SEC22b OS=Homo sapiens OX=9606 GN=SEC22B PE=1 SV=4 | 20 | 3 | 3 | 3 | 215 | 24.6 | 6.92 | 6.52 | 3 | 1 |
| P62906 | 60S ribosomal protein L10a OS=Homo sapiens OX=9606 GN=RPL10A PE=1 SV=2 | 10 | 2 | 3 | 2 | 217 | 24.8 | 9.94 | 6.51 | 2 | 1 |
| O95817 | BAG family molecular chaperone regulator 3 OS=Homo sapiens OX=9606 GN=BAG3 PE=1 SV=3 | 6 | 3 | 3 | 3 | 575 | 61.6 | 6.95 | 6.5 | 3 | 1 |
| P63104 | 14-3-3 protein zeta/delta OS=Homo sapiens OX=9606 GN=YWHAZ PE=1 SV=1 | 6 | 1 | 2 | 1 | 245 | 27.7 | 4.79 | 6.46 | 1 | 1 |
| P30101 | Protein disulfide-isomerase A3 OS=Homo sapiens OX=9606 GN=PDIA3 PE=1 SV=4 | 7 | 3 | 3 | 3 | 505 | 56.7 | 6.35 | 6.41 | 3 | 1 |
| P15924 | Desmoplakin OS=Homo sapiens OX=9606 GN=DSP PE=1 SV=3 | 1 | 3 | 3 | 3 | 2871 | 331.6 | 6.81 | 6.39 | 3 | 1 |
| Q9UL25 | Ras-related protein Rab-21 OS=Homo sapiens OX=9606 GN=RAB21 PE=1 SV=3 | 7 | 1 | 2 | 1 | 225 | 24.3 | 7.94 | 6.38 | 1 | 1 |
| P82673 | 28S ribosomal protein S35, mitochondrial OS=Homo sapiens OX=9606 GN=MRPS35 PE=1 SV=1 | 7 | 2 | 3 | 2 | 323 | 36.8 | 8.24 | 6.33 | 2 | 1 |
| P55884 | Eukaryotic translation initiation factor 3 subunit B OS=Homo sapiens OX=9606 GN=EIF3B PE=1 SV=3 | 4 | 3 | 3 | 3 | 814 | 92.4 | 5 | 6.29 | 3 | 1 |
| P05549 | Transcription factor AP-2-alpha OS=Homo sapiens OX=9606 GN=TFAP2A PE=1 SV=1 | 7 | 3 | 3 | 3 | 437 | 48 | 8.02 | 6.2 | 3 | 1 |
| P49207 | 60S ribosomal protein L34 OS=Homo sapiens OX=9606 GN=RPL34 PE=1 SV=3 | 22 | 3 | 3 | 3 | 117 | 13.3 | 11.47 | 6.19 | 3 | 1 |
| Q9Y676 | 28S ribosomal protein S18b, mitochondrial OS=Homo sapiens OX=9606 GN=MRPS18B PE=1 SV=1 | 14 | 3 | 3 | 3 | 258 | 29.4 | 9.38 | 6.12 | 3 | 1 |
| P37108 | Signal recognition particle 14 kDa protein OS=Homo sapiens OX=9606 GN=SRP14 PE=1 SV=2 | 15 | 2 | 2 | 2 | 136 | 14.6 | 10.04 | 6.09 | 2 | 1 |
| Q8NCA5 | Protein FAM98A OS=Homo sapiens OX=9606 GN=FAM98A PE=1 SV=2 | 6 | 2 | 2 | 2 | 518 | 55.2 | 8.95 | 6.03 | 2 | 1 |
| P46776 | 60S ribosomal protein L27a OS=Homo sapiens OX=9606 GN=RPL27A PE=1 SV=2 | 7 | 1 | 2 | 1 | 148 | 16.6 | 11 | 5.99 | 1 | 1 |
| P62306 | Small nuclear ribonucleoprotein F OS=Homo sapiens OX=9606 GN=SNRPF PE=1 SV=1 | 15 | 1 | 2 | 1 | 86 | 9.7 | 4.67 | 5.86 | 1 | 1 |
| P07477 | Trypsin-1 OS=Homo sapiens OX=9606 GN=PRSS1 PE=1 SV=1 | 11 | 2 | 2 | 2 | 247 | 26.5 | 6.51 | 5.84 | 2 | 1 |
| P00533 | Epidermal growth factor receptor OS=Homo sapiens OX=9606 GN=EGFR PE=1 SV=2 | 2 | 2 | 2 | 2 | 1210 | 134.2 | 6.68 | 5.78 | 2 | 1 |
| P82664 | 28S ribosomal protein S10, mitochondrial OS=Homo sapiens OX=9606 GN=MRPS10 PE=1 SV=2 | 11 | 2 | 2 | 2 | 201 | 23 | 8 | 5.47 | 2 | 1 |
| P23588 | Eukaryotic translation initiation factor 4B OS=Homo sapiens OX=9606 GN=EIF4B PE=1 SV=2 | 4 | 2 | 2 | 2 | 611 | 69.1 | 5.73 | 5.44 | 2 | 1 |
| P62861 | 40S ribosomal protein S30 OS=Homo sapiens OX=9606 GN=FAU PE=1 SV=1 | 17 | 1 | 2 | 1 | 59 | 6.6 | 12.15 | 5.37 | 1 | 1 |
| P11586 | C-1-tetrahydrofolate synthase, cytoplasmic OS=Homo sapiens OX=9606 GN=MTHFD1 PE=1 SV=3 | 3 | 2 | 2 | 2 | 935 | 101.5 | 7.3 | 5.34 | 2 | 1 |
| P21291 | Cysteine and glycine-rich protein 1 OS=Homo sapiens OX=9606 GN=CSRP1 PE=1 SV=3 | 16 | 2 | 2 | 2 | 193 | 20.6 | 8.57 | 5.26 | 2 | 1 |
| O75822 | Eukaryotic translation initiation factor 3 subunit J OS=Homo sapiens OX=9606 GN=EIF3J PE=1 SV=2 | 9 | 2 | 2 | 2 | 258 | 29 | 4.83 | 5.23 | 2 | 1 |
| P62805 | Histone H4 OS=Homo sapiens OX=9606 GN=H4C1 PE=1 SV=2 | 17 | 2 | 2 | 2 | 103 | 11.4 | 11.36 | 5.21 | 2 | 1 |
| P62829 | 60S ribosomal protein L23 OS=Homo sapiens OX=9606 GN=RPL23 PE=1 SV=1 | 18 | 2 | 2 | 2 | 140 | 14.9 | 10.51 | 5.2 | 2 | 1 |
| Q9NZ01 | Very-long-chain enoyl-CoA reductase OS=Homo sapiens OX=9606 GN=TECR PE=1 SV=1 | 3 | 1 | 2 | 1 | 308 | 36 | 9.45 | 5.18 | 1 | 1 |
| Q3MHD2 | Protein LSM12 homolog OS=Homo sapiens OX=9606 GN=LSM12 PE=1 SV=2 | 14 | 2 | 2 | 2 | 195 | 21.7 | 7.74 | 5.17 | 2 | 1 |
| P48643 | T-complex protein 1 subunit epsilon OS=Homo sapiens OX=9606 GN=CCT5 PE=1 SV=1 | 4 | 2 | 2 | 2 | 541 | 59.6 | 5.66 | 5.17 | 2 | 1 |
| P35030 | Trypsin-3 OS=Homo sapiens OX=9606 GN=PRSS3 PE=1 SV=2 | 4 | 1 | 2 | 1 | 304 | 32.5 | 7.49 | 5.17 | 1 | 1 |
| Q3ZCQ8 | Mitochondrial import inner membrane translocase subunit TIM50 OS=Homo sapiens OX=9606 GN=TIMM50 PE=1 SV=2 | 3 | 1 | 2 | 1 | 353 | 39.6 | 8.37 | 5.15 | 1 | 1 |
| P31025 | Lipocalin-1 OS=Homo sapiens OX=9606 GN=LCN1 PE=1 SV=1 | 13 | 2 | 2 | 2 | 176 | 19.2 | 5.58 | 5.11 | 2 | 1 |
| Q14134 | Tripartite motif-containing protein 29 OS=Homo sapiens OX=9606 GN=TRIM29 PE=1 SV=2 | 4 | 2 | 2 | 2 | 588 | 65.8 | 7.15 | 5.06 | 2 | 1 |
| P49368 | T-complex protein 1 subunit gamma OS=Homo sapiens OX=9606 GN=CCT3 PE=1 SV=4 | 4 | 2 | 2 | 2 | 545 | 60.5 | 6.49 | 5.05 | 2 | 1 |
| P08579 | U2 small nuclear ribonucleoprotein B'' OS=Homo sapiens OX=9606 GN=SNRPB2 PE=1 SV=1 | 13 | 2 | 2 | 2 | 225 | 25.5 | 9.72 | 4.98 | 2 | 1 |
| P08195 | 4F2 cell-surface antigen heavy chain OS=Homo sapiens OX=9606 GN=SLC3A2 PE=1 SV=3 | 4 | 2 | 2 | 2 | 630 | 68 | 5.01 | 4.98 | 2 | 1 |
| P42677 | 40S ribosomal protein S27 OS=Homo sapiens OX=9606 GN=RPS27 PE=1 SV=3 | 13 | 1 | 2 | 1 | 84 | 9.5 | 9.45 | 4.9 | 1 | 1 |
| P14678 | Small nuclear ribonucleoprotein-associated proteins B and B' OS=Homo sapiens OX=9606 GN=SNRPB PE=1 SV=2 | 6 | 1 | 2 | 1 | 240 | 24.6 | 11.19 | 4.9 | 1 | 1 |
| P11182 | Lipoamide acyltransferase component of branched-chain alpha-keto acid dehydrogenase complex, mitochondrial OS=Homo sapiens OX=9606 GN=DBT PE=1 SV=3 | 4 | 2 | 2 | 2 | 482 | 53.5 | 8.51 | 4.82 | 2 | 1 |
| Q86WX3 | Active regulator of SIRT1 OS=Homo sapiens OX=9606 GN=RPS19BP1 PE=1 SV=1 | 18 | 2 | 2 | 2 | 136 | 15.4 | 10.74 | 4.8 | 2 | 1 |
| Q8NC51 | Plasminogen activator inhibitor 1 RNA-binding protein OS=Homo sapiens OX=9606 GN=SERBP1 PE=1 SV=2 | 7 | 2 | 2 | 2 | 408 | 44.9 | 8.65 | 4.8 | 2 | 1 |
| Q14244 | Ensconsin OS=Homo sapiens OX=9606 GN=MAP7 PE=1 SV=1 | 3 | 2 | 2 | 2 | 749 | 84 | 9.61 | 4.79 | 2 | 1 |
| Q9Y3D9 | 28S ribosomal protein S23, mitochondrial OS=Homo sapiens OX=9606 GN=MRPS23 PE=1 SV=2 | 11 | 2 | 2 | 2 | 190 | 21.8 | 8.9 | 4.78 | 2 | 1 |
| Q6DD87 | Zinc finger protein 787 OS=Homo sapiens OX=9606 GN=ZNF787 PE=1 SV=4 | 8 | 2 | 2 | 2 | 382 | 40.4 | 7.96 | 4.74 | 2 | 1 |
| Q15046 | Lysine--tRNA ligase OS=Homo sapiens OX=9606 GN=KARS1 PE=1 SV=3 | 4 | 2 | 2 | 2 | 597 | 68 | 6.35 | 4.73 | 2 | 1 |
| O00303 | Eukaryotic translation initiation factor 3 subunit F OS=Homo sapiens OX=9606 GN=EIF3F PE=1 SV=1 | 7 | 2 | 2 | 2 | 357 | 37.5 | 5.45 | 4.72 | 2 | 1 |
| P63244 | Receptor of activated protein C kinase 1 OS=Homo sapiens OX=9606 GN=RACK1 PE=1 SV=3 | 7 | 2 | 2 | 2 | 317 | 35.1 | 7.69 | 4.72 | 2 | 1 |
| Q15717 | ELAV-like protein 1 OS=Homo sapiens OX=9606 GN=ELAVL1 PE=1 SV=2 | 7 | 2 | 2 | 2 | 326 | 36.1 | 9.17 | 4.7 | 2 | 1 |
| P38919 | Eukaryotic initiation factor 4A-III OS=Homo sapiens OX=9606 GN=EIF4A3 PE=1 SV=4 | 5 | 2 | 2 | 1 | 411 | 46.8 | 6.73 | 4.69 | 2 | 1 |
| P46782 | 40S ribosomal protein S5 OS=Homo sapiens OX=9606 GN=RPS5 PE=1 SV=4 | 4 | 1 | 2 | 1 | 204 | 22.9 | 9.72 | 4.67 | 1 | 1 |
| P09661 | U2 small nuclear ribonucleoprotein A' OS=Homo sapiens OX=9606 GN=SNRPA1 PE=1 SV=2 | 5 | 1 | 2 | 1 | 255 | 28.4 | 8.62 | 4.66 | 1 | 1 |
| P46777 | 60S ribosomal protein L5 OS=Homo sapiens OX=9606 GN=RPL5 PE=1 SV=3 | 9 | 2 | 2 | 2 | 297 | 34.3 | 9.72 | 4.64 | 2 | 1 |
| P62241 | 40S ribosomal protein S8 OS=Homo sapiens OX=9606 GN=RPS8 PE=1 SV=2 | 13 | 2 | 2 | 2 | 208 | 24.2 | 10.32 | 4.62 | 2 | 1 |
| P62081 | 40S ribosomal protein S7 OS=Homo sapiens OX=9606 GN=RPS7 PE=1 SV=1 | 9 | 2 | 2 | 2 | 194 | 22.1 | 10.1 | 4.62 | 2 | 1 |
| O43670 | BUB3-interacting and GLEBS motif-containing protein ZNF207 OS=Homo sapiens OX=9606 GN=ZNF207 PE=1 SV=1 | 5 | 2 | 2 | 2 | 478 | 50.7 | 9.1 | 4.61 | 2 | 1 |
| P26641 | Elongation factor 1-gamma OS=Homo sapiens OX=9606 GN=EEF1G PE=1 SV=3 | 5 | 2 | 2 | 2 | 437 | 50.1 | 6.67 | 4.58 | 2 | 1 |
| P25311 | Zinc-alpha-2-glycoprotein OS=Homo sapiens OX=9606 GN=AZGP1 PE=1 SV=2 | 3 | 1 | 2 | 1 | 298 | 34.2 | 6.05 | 4.58 | 1 | 1 |
| Q5T9A4 | ATPase family AAA domain-containing protein 3B OS=Homo sapiens OX=9606 GN=ATAD3B PE=1 SV=1 | 3 | 2 | 2 | 2 | 648 | 72.5 | 9.2 | 4.57 | 2 | 1 |
| P49750 | YLP motif-containing protein 1 OS=Homo sapiens OX=9606 GN=YLPM1 PE=1 SV=4 | 1 | 2 | 2 | 2 | 2146 | 241.5 | 6.55 | 4.56 | 2 | 1 |
| Q03013 | Glutathione S-transferase Mu 4 OS=Homo sapiens OX=9606 GN=GSTM4 PE=1 SV=3 | 6 | 1 | 2 | 1 | 218 | 25.5 | 5.9 | 4.55 | 1 | 1 |
| P31153 | S-adenosylmethionine synthase isoform type-2 OS=Homo sapiens OX=9606 GN=MAT2A PE=1 SV=1 | 4 | 1 | 2 | 1 | 395 | 43.6 | 6.48 | 4.53 | 1 | 1 |
| P16615 | Sarcoplasmic/endoplasmic reticulum calcium ATPase 2 OS=Homo sapiens OX=9606 GN=ATP2A2 PE=1 SV=1 | 3 | 2 | 2 | 2 | 1042 | 114.7 | 5.34 | 4.5 | 2 | 1 |
| O95983 | Methyl-CpG-binding domain protein 3 OS=Homo sapiens OX=9606 GN=MBD3 PE=1 SV=1 | 3 | 1 | 2 | 1 | 291 | 32.8 | 5.34 | 4.49 | 1 | 1 |
| Q14847 | LIM and SH3 domain protein 1 OS=Homo sapiens OX=9606 GN=LASP1 PE=1 SV=2 | 9 | 2 | 2 | 2 | 261 | 29.7 | 7.05 | 4.47 | 2 | 1 |
| P40227 | T-complex protein 1 subunit zeta OS=Homo sapiens OX=9606 GN=CCT6A PE=1 SV=3 | 3 | 2 | 2 | 2 | 531 | 58 | 6.68 | 4.44 | 2 | 1 |
| Q8N5N7 | 39S ribosomal protein L50, mitochondrial OS=Homo sapiens OX=9606 GN=MRPL50 PE=1 SV=2 | 15 | 2 | 2 | 2 | 158 | 18.3 | 7.88 | 4.42 | 2 | 1 |
| P62888 | 60S ribosomal protein L30 OS=Homo sapiens OX=9606 GN=RPL30 PE=1 SV=2 | 20 | 2 | 2 | 2 | 115 | 12.8 | 9.63 | 4.41 | 2 | 1 |
| O15427 | Monocarboxylate transporter 4 OS=Homo sapiens OX=9606 GN=SLC16A3 PE=1 SV=1 | 6 | 2 | 2 | 2 | 465 | 49.4 | 7.96 | 4.41 | 2 | 1 |
| Q8TDN6 | Ribosome biogenesis protein BRX1 homolog OS=Homo sapiens OX=9606 GN=BRIX1 PE=1 SV=2 | 7 | 2 | 2 | 2 | 353 | 41.4 | 9.92 | 4.39 | 2 | 1 |
| Q13347 | Eukaryotic translation initiation factor 3 subunit I OS=Homo sapiens OX=9606 GN=EIF3I PE=1 SV=1 | 6 | 2 | 2 | 2 | 325 | 36.5 | 5.64 | 4.39 | 2 | 1 |
| P52272 | Heterogeneous nuclear ribonucleoprotein M OS=Homo sapiens OX=9606 GN=HNRNPM PE=1 SV=3 | 2 | 2 | 2 | 2 | 730 | 77.5 | 8.7 | 4.39 | 2 | 1 |
| O43143 | Pre-mRNA-splicing factor ATP-dependent RNA helicase DHX15 OS=Homo sapiens OX=9606 GN=DHX15 PE=1 SV=2 | 3 | 2 | 2 | 2 | 795 | 90.9 | 7.46 | 4.3 | 2 | 1 |
| O00422 | Histone deacetylase complex subunit SAP18 OS=Homo sapiens OX=9606 GN=SAP18 PE=1 SV=1 | 14 | 2 | 2 | 2 | 153 | 17.6 | 9.35 | 4.27 | 2 | 1 |
| P02788 | Lactotransferrin OS=Homo sapiens OX=9606 GN=LTF PE=1 SV=6 | 4 | 2 | 2 | 2 | 710 | 78.1 | 8.12 | 4.27 | 2 | 1 |
| P06493 | Cyclin-dependent kinase 1 OS=Homo sapiens OX=9606 GN=CDK1 PE=1 SV=3 | 9 | 2 | 2 | 2 | 297 | 34.1 | 8.4 | 4.25 | 2 | 1 |
| P18085 | ADP-ribosylation factor 4 OS=Homo sapiens OX=9606 GN=ARF4 PE=1 SV=3 | 12 | 2 | 2 | 2 | 180 | 20.5 | 7.14 | 4.2 | 2 | 1 |
| P27635 | 60S ribosomal protein L10 OS=Homo sapiens OX=9606 GN=RPL10 PE=1 SV=4 | 4 | 1 | 2 | 1 | 214 | 24.6 | 10.08 | 4.18 | 1 | 1 |
| P13489 | Ribonuclease inhibitor OS=Homo sapiens OX=9606 GN=RNH1 PE=1 SV=2 | 6 | 2 | 2 | 2 | 461 | 49.9 | 4.82 | 4.08 | 2 | 1 |
| Q96AE4 | Far upstream element-binding protein 1 OS=Homo sapiens OX=9606 GN=FUBP1 PE=1 SV=3 | 3 | 2 | 2 | 2 | 644 | 67.5 | 7.61 | 4.08 | 2 | 1 |
| Q15029 | 116 kDa U5 small nuclear ribonucleoprotein component OS=Homo sapiens OX=9606 GN=EFTUD2 PE=1 SV=1 | 2 | 2 | 2 | 2 | 972 | 109.4 | 5 | 4.07 | 2 | 1 |
| P62701 | 40S ribosomal protein S4, X isoform OS=Homo sapiens OX=9606 GN=RPS4X PE=1 SV=2 | 8 | 2 | 2 | 2 | 263 | 29.6 | 10.15 | 4.06 | 2 | 1 |
| P50991 | T-complex protein 1 subunit delta OS=Homo sapiens OX=9606 GN=CCT4 PE=1 SV=4 | 5 | 2 | 2 | 2 | 539 | 57.9 | 7.83 | 4.06 | 2 | 1 |
| P08559 | Pyruvate dehydrogenase E1 component subunit alpha, somatic form, mitochondrial OS=Homo sapiens OX=9606 GN=PDHA1 PE=1 SV=3 | 7 | 2 | 2 | 2 | 390 | 43.3 | 8.06 | 4.05 | 2 | 1 |
| P02042 | Hemoglobin subunit delta OS=Homo sapiens OX=9606 GN=HBD PE=1 SV=2 | 6 | 1 | 2 | 1 | 147 | 16 | 8.05 | 4.04 | 1 | 1 |
| O00425 | Insulin-like growth factor 2 mRNA-binding protein 3 OS=Homo sapiens OX=9606 GN=IGF2BP3 PE=1 SV=2 | 4 | 2 | 2 | 2 | 579 | 63.7 | 8.87 | 4.02 | 2 | 1 |
| P78527 | DNA-dependent protein kinase catalytic subunit OS=Homo sapiens OX=9606 GN=PRKDC PE=1 SV=3 | 1 | 2 | 2 | 2 | 4128 | 468.8 | 7.12 | 4.02 | 2 | 1 |
| Q13242 | Serine/arginine-rich splicing factor 9 OS=Homo sapiens OX=9606 GN=SRSF9 PE=1 SV=1 | 9 | 2 | 2 | 2 | 221 | 25.5 | 8.65 | 3.97 | 2 | 1 |
| P24752 | Acetyl-CoA acetyltransferase, mitochondrial OS=Homo sapiens OX=9606 GN=ACAT1 PE=1 SV=1 | 6 | 2 | 2 | 2 | 427 | 45.2 | 8.85 | 3.94 | 2 | 1 |
| P51149 | Ras-related protein Rab-7a OS=Homo sapiens OX=9606 GN=RAB7A PE=1 SV=1 | 10 | 2 | 2 | 2 | 207 | 23.5 | 6.7 | 3.92 | 2 | 1 |
| Q96AG4 | Leucine-rich repeat-containing protein 59 OS=Homo sapiens OX=9606 GN=LRRC59 PE=1 SV=1 | 5 | 2 | 2 | 2 | 307 | 34.9 | 9.57 | 3.9 | 2 | 1 |
| P62195 | 26S proteasome regulatory subunit 8 OS=Homo sapiens OX=9606 GN=PSMC5 PE=1 SV=1 | 7 | 2 | 2 | 2 | 406 | 45.6 | 7.55 | 3.89 | 2 | 1 |
| Q9NY12 | H/ACA ribonucleoprotein complex subunit 1 OS=Homo sapiens OX=9606 GN=GAR1 PE=1 SV=1 | 8 | 2 | 2 | 2 | 217 | 22.3 | 10.92 | 3.88 | 2 | 1 |
| O00148 | ATP-dependent RNA helicase DDX39A OS=Homo sapiens OX=9606 GN=DDX39A PE=1 SV=2 | 5 | 2 | 2 | 2 | 427 | 49.1 | 5.68 | 3.83 | 2 | 1 |
| P33993 | DNA replication licensing factor MCM7 OS=Homo sapiens OX=9606 GN=MCM7 PE=1 SV=4 | 4 | 2 | 2 | 2 | 719 | 81.3 | 6.46 | 3.82 | 2 | 1 |
| Q86U42 | Polyadenylate-binding protein 2 OS=Homo sapiens OX=9606 GN=PABPN1 PE=1 SV=3 | 6 | 1 | 1 | 1 | 306 | 32.7 | 5.06 | 3.74 | 1 | 1 |
| Q16778 | Histone H2B type 2-E OS=Homo sapiens OX=9606 GN=H2BC21 PE=1 SV=3 | 9 | 1 | 1 | 1 | 126 | 13.9 | 10.32 | 3.62 | 1 | 1 |
| Q9BYD3 | 39S ribosomal protein L4, mitochondrial OS=Homo sapiens OX=9606 GN=MRPL4 PE=1 SV=1 | 6 | 1 | 1 | 1 | 311 | 34.9 | 9.72 | 3.41 | 1 | 1 |
| O15372 | Eukaryotic translation initiation factor 3 subunit H OS=Homo sapiens OX=9606 GN=EIF3H PE=1 SV=1 | 5 | 1 | 1 | 1 | 352 | 39.9 | 6.54 | 3.24 | 1 | 1 |
| P31040 | Succinate dehydrogenase [ubiquinone] flavoprotein subunit, mitochondrial OS=Homo sapiens OX=9606 GN=SDHA PE=1 SV=2 | 2 | 1 | 1 | 1 | 664 | 72.6 | 7.39 | 3.21 | 1 | 1 |
| Q92504 | Zinc transporter SLC39A7 OS=Homo sapiens OX=9606 GN=SLC39A7 PE=1 SV=2 | 3 | 1 | 1 | 1 | 469 | 50.1 | 6.87 | 3.13 | 1 | 1 |
| Q9Y3B9 | RRP15-like protein OS=Homo sapiens OX=9606 GN=RRP15 PE=1 SV=2 | 5 | 1 | 1 | 1 | 282 | 31.5 | 5.52 | 3.05 | 1 | 1 |
| P60981 | Destrin OS=Homo sapiens OX=9606 GN=DSTN PE=1 SV=3 | 5 | 1 | 1 | 1 | 165 | 18.5 | 7.85 | 2.95 | 1 | 1 |
| Q13601 | KRR1 small subunit processome component homolog OS=Homo sapiens OX=9606 GN=KRR1 PE=1 SV=4 | 3 | 1 | 1 | 1 | 381 | 43.6 | 9.77 | 2.93 | 1 | 1 |
| Q13283 | Ras GTPase-activating protein-binding protein 1 OS=Homo sapiens OX=9606 GN=G3BP1 PE=1 SV=1 | 2 | 1 | 1 | 1 | 466 | 52.1 | 5.52 | 2.93 | 1 | 1 |
| P12004 | Proliferating cell nuclear antigen OS=Homo sapiens OX=9606 GN=PCNA PE=1 SV=1 | 5 | 1 | 1 | 1 | 261 | 28.8 | 4.69 | 2.92 | 1 | 1 |
| Q9Y2R9 | 28S ribosomal protein S7, mitochondrial OS=Homo sapiens OX=9606 GN=MRPS7 PE=1 SV=2 | 5 | 1 | 1 | 1 | 242 | 28.1 | 9.99 | 2.89 | 1 | 1 |
| Q00059 | Transcription factor A, mitochondrial OS=Homo sapiens OX=9606 GN=TFAM PE=1 SV=1 | 4 | 1 | 1 | 1 | 246 | 29.1 | 9.72 | 2.89 | 1 | 1 |
| Q14257 | Reticulocalbin-2 OS=Homo sapiens OX=9606 GN=RCN2 PE=1 SV=1 | 3 | 1 | 1 | 1 | 317 | 36.9 | 4.4 | 2.89 | 1 | 1 |
| P61604 | 10 kDa heat shock protein, mitochondrial OS=Homo sapiens OX=9606 GN=HSPE1 PE=1 SV=2 | 14 | 1 | 1 | 1 | 102 | 10.9 | 8.92 | 2.87 | 1 | 1 |
| P09132 | Signal recognition particle 19 kDa protein OS=Homo sapiens OX=9606 GN=SRP19 PE=1 SV=3 | 10 | 1 | 1 | 1 | 144 | 16.1 | 9.85 | 2.87 | 1 | 1 |
| P08574 | Cytochrome c1, heme protein, mitochondrial OS=Homo sapiens OX=9606 GN=CYC1 PE=1 SV=3 | 4 | 1 | 1 | 1 | 325 | 35.4 | 9 | 2.85 | 1 | 1 |
| Q9H0G5 | Nuclear speckle splicing regulatory protein 1 OS=Homo sapiens OX=9606 GN=NSRP1 PE=1 SV=1 | 2 | 1 | 1 | 1 | 558 | 66.4 | 8.84 | 2.83 | 1 | 1 |
| P50213 | Isocitrate dehydrogenase [NAD] subunit alpha, mitochondrial OS=Homo sapiens OX=9606 GN=IDH3A PE=1 SV=1 | 3 | 1 | 1 | 1 | 366 | 39.6 | 6.92 | 2.78 | 1 | 1 |
| P42765 | 3-ketoacyl-CoA thiolase, mitochondrial OS=Homo sapiens OX=9606 GN=ACAA2 PE=1 SV=2 | 3 | 1 | 1 | 1 | 397 | 41.9 | 8.09 | 2.76 | 1 | 1 |
| Q08554 | Desmocollin-1 OS=Homo sapiens OX=9606 GN=DSC1 PE=1 SV=2 | 2 | 1 | 1 | 1 | 894 | 99.9 | 5.43 | 2.76 | 1 | 1 |
| Q8TC12 | Retinol dehydrogenase 11 OS=Homo sapiens OX=9606 GN=RDH11 PE=1 SV=2 | 4 | 1 | 1 | 1 | 318 | 35.4 | 8.82 | 2.74 | 1 | 1 |
| Q13405 | 39S ribosomal protein L49, mitochondrial OS=Homo sapiens OX=9606 GN=MRPL49 PE=1 SV=1 | 8 | 1 | 1 | 1 | 166 | 19.2 | 9.45 | 2.73 | 1 | 1 |
| Q9NQT5 | Exosome complex component RRP40 OS=Homo sapiens OX=9606 GN=EXOSC3 PE=1 SV=3 | 6 | 1 | 1 | 1 | 275 | 29.6 | 8.1 | 2.69 | 1 | 1 |
| Q9UI30 | Multifunctional methyltransferase subunit TRM112-like protein OS=Homo sapiens OX=9606 GN=TRMT112 PE=1 SV=1 | 11 | 1 | 1 | 1 | 125 | 14.2 | 5.26 | 2.68 | 1 | 1 |
| P52209 | 6-phosphogluconate dehydrogenase, decarboxylating OS=Homo sapiens OX=9606 GN=PGD PE=1 SV=3 | 4 | 1 | 1 | 1 | 483 | 53.1 | 7.23 | 2.68 | 1 | 1 |
| P59998 | Actin-related protein 2/3 complex subunit 4 OS=Homo sapiens OX=9606 GN=ARPC4 PE=1 SV=3 | 7 | 1 | 1 | 1 | 168 | 19.7 | 8.43 | 2.67 | 1 | 1 |
| Q13151 | Heterogeneous nuclear ribonucleoprotein A0 OS=Homo sapiens OX=9606 GN=HNRNPA0 PE=1 SV=1 | 5 | 1 | 1 | 1 | 305 | 30.8 | 9.29 | 2.66 | 1 | 1 |
| P06454 | Prothymosin alpha OS=Homo sapiens OX=9606 GN=PTMA PE=1 SV=2 | 14 | 1 | 1 | 1 | 111 | 12.2 | 3.78 | 2.64 | 1 | 1 |
| E9PRG8 | Uncharacterized protein C11orf98 OS=Homo sapiens OX=9606 GN=C11orf98 PE=4 SV=2 | 11 | 1 | 1 | 1 | 123 | 14.2 | 11.53 | 2.62 | 1 | 1 |
| Q9UJZ1 | Stomatin-like protein 2, mitochondrial OS=Homo sapiens OX=9606 GN=STOML2 PE=1 SV=1 | 4 | 1 | 1 | 1 | 356 | 38.5 | 7.39 | 2.59 | 1 | 1 |
| Q9GZZ1 | N-alpha-acetyltransferase 50 OS=Homo sapiens OX=9606 GN=NAA50 PE=1 SV=1 | 8 | 1 | 1 | 1 | 169 | 19.4 | 8.81 | 2.57 | 1 | 1 |
| O15479 | Melanoma-associated antigen B2 OS=Homo sapiens OX=9606 GN=MAGEB2 PE=1 SV=3 | 5 | 1 | 1 | 1 | 319 | 35.3 | 8.76 | 2.56 | 1 | 1 |
| P11498 | Pyruvate carboxylase, mitochondrial OS=Homo sapiens OX=9606 GN=PC PE=1 SV=2 | 1 | 1 | 1 | 1 | 1178 | 129.6 | 6.84 | 2.56 | 1 | 1 |
| Q9Y3Y2 | Chromatin target of PRMT1 protein OS=Homo sapiens OX=9606 GN=CHTOP PE=1 SV=2 | 6 | 1 | 1 | 1 | 248 | 26.4 | 12.23 | 2.54 | 1 | 1 |
| Q8N7H5 | RNA polymerase II-associated factor 1 homolog OS=Homo sapiens OX=9606 GN=PAF1 PE=1 SV=2 | 2 | 1 | 1 | 1 | 531 | 59.9 | 4.63 | 2.52 | 1 | 1 |
| Q8N983 | 39S ribosomal protein L43, mitochondrial OS=Homo sapiens OX=9606 GN=MRPL43 PE=1 SV=1 | 5 | 1 | 1 | 1 | 215 | 23.4 | 8.65 | 2.51 | 1 | 1 |
| Q9UBB5 | Methyl-CpG-binding domain protein 2 OS=Homo sapiens OX=9606 GN=MBD2 PE=1 SV=1 | 2 | 1 | 1 | 1 | 411 | 43.2 | 10.04 | 2.51 | 1 | 1 |
| Q1ED39 | Lysine-rich nucleolar protein 1 OS=Homo sapiens OX=9606 GN=KNOP1 PE=1 SV=1 | 2 | 1 | 1 | 1 | 458 | 51.6 | 9.86 | 2.51 | 1 | 1 |
| Q6P161 | 39S ribosomal protein L54, mitochondrial OS=Homo sapiens OX=9606 GN=MRPL54 PE=1 SV=1 | 7 | 1 | 1 | 1 | 138 | 15.8 | 9.6 | 2.49 | 1 | 1 |
| Q9BY77 | Polymerase delta-interacting protein 3 OS=Homo sapiens OX=9606 GN=POLDIP3 PE=1 SV=2 | 4 | 1 | 1 | 1 | 421 | 46.1 | 9.99 | 2.49 | 1 | 1 |
| P20674 | Cytochrome c oxidase subunit 5A, mitochondrial OS=Homo sapiens OX=9606 GN=COX5A PE=1 SV=2 | 6 | 1 | 1 | 1 | 150 | 16.8 | 6.79 | 2.47 | 1 | 1 |
| P13010 | X-ray repair cross-complementing protein 5 OS=Homo sapiens OX=9606 GN=XRCC5 PE=1 SV=3 | 2 | 1 | 1 | 1 | 732 | 82.7 | 5.81 | 2.46 | 1 | 1 |
| Q13573 | SNW domain-containing protein 1 OS=Homo sapiens OX=9606 GN=SNW1 PE=1 SV=1 | 2 | 1 | 1 | 1 | 536 | 61.5 | 9.52 | 2.45 | 1 | 1 |
| Q9GZT3 | SRA stem-loop-interacting RNA-binding protein, mitochondrial OS=Homo sapiens OX=9606 GN=SLIRP PE=1 SV=1 | 13 | 1 | 1 | 1 | 109 | 12.3 | 10.24 | 2.44 | 1 | 1 |
| Q13561 | Dynactin subunit 2 OS=Homo sapiens OX=9606 GN=DCTN2 PE=1 SV=4 | 2 | 1 | 1 | 1 | 401 | 44.2 | 5.21 | 2.44 | 1 | 1 |
| Q96EY1 | DnaJ homolog subfamily A member 3, mitochondrial OS=Homo sapiens OX=9606 GN=DNAJA3 PE=1 SV=2 | 3 | 1 | 1 | 1 | 480 | 52.5 | 9.26 | 2.42 | 1 | 1 |
| Q9UQ80 | Proliferation-associated protein 2G4 OS=Homo sapiens OX=9606 GN=PA2G4 PE=1 SV=3 | 3 | 1 | 1 | 1 | 394 | 43.8 | 6.55 | 2.42 | 1 | 1 |
| Q16630 | Cleavage and polyadenylation specificity factor subunit 6 OS=Homo sapiens OX=9606 GN=CPSF6 PE=1 SV=2 | 3 | 1 | 1 | 1 | 551 | 59.2 | 7.15 | 2.41 | 1 | 1 |
| Q15056 | Eukaryotic translation initiation factor 4H OS=Homo sapiens OX=9606 GN=EIF4H PE=1 SV=5 | 5 | 1 | 1 | 1 | 248 | 27.4 | 7.23 | 2.39 | 1 | 1 |
| Q9H9B4 | Sideroflexin-1 OS=Homo sapiens OX=9606 GN=SFXN1 PE=1 SV=4 | 4 | 1 | 1 | 1 | 322 | 35.6 | 9.07 | 2.39 | 1 | 1 |
| P01876 | Immunoglobulin heavy constant alpha 1 OS=Homo sapiens OX=9606 GN=IGHA1 PE=1 SV=2 | 3 | 1 | 1 | 1 | 353 | 37.6 | 6.51 | 2.38 | 1 | 1 |
| Q92598 | Heat shock protein 105 kDa OS=Homo sapiens OX=9606 GN=HSPH1 PE=1 SV=1 | 2 | 1 | 1 | 1 | 858 | 96.8 | 5.39 | 2.38 | 1 | 1 |
| Q8IWX8 | Calcium homeostasis endoplasmic reticulum protein OS=Homo sapiens OX=9606 GN=CHERP PE=1 SV=3 | 2 | 1 | 1 | 1 | 916 | 103.6 | 9.04 | 2.36 | 1 | 1 |
| Q9Y3B7 | 39S ribosomal protein L11, mitochondrial OS=Homo sapiens OX=9606 GN=MRPL11 PE=1 SV=1 | 6 | 1 | 1 | 1 | 192 | 20.7 | 9.91 | 2.35 | 1 | 1 |
| P07237 | Protein disulfide-isomerase OS=Homo sapiens OX=9606 GN=P4HB PE=1 SV=3 | 3 | 1 | 1 | 1 | 508 | 57.1 | 4.87 | 2.35 | 1 | 1 |
| O75955 | Flotillin-1 OS=Homo sapiens OX=9606 GN=FLOT1 PE=1 SV=3 | 3 | 1 | 1 | 1 | 427 | 47.3 | 7.49 | 2.35 | 1 | 1 |
| P21796 | Voltage-dependent anion-selective channel protein 1 OS=Homo sapiens OX=9606 GN=VDAC1 PE=1 SV=2 | 7 | 1 | 1 | 1 | 283 | 30.8 | 8.54 | 2.34 | 1 | 1 |
| Q15637 | Splicing factor 1 OS=Homo sapiens OX=9606 GN=SF1 PE=1 SV=4 | 2 | 1 | 1 | 1 | 639 | 68.3 | 8.98 | 2.34 | 1 | 1 |
| O15234 | Protein CASC3 OS=Homo sapiens OX=9606 GN=CASC3 PE=1 SV=2 | 2 | 1 | 1 | 1 | 703 | 76.2 | 6.48 | 2.34 | 1 | 1 |
| P34897 | Serine hydroxymethyltransferase, mitochondrial OS=Homo sapiens OX=9606 GN=SHMT2 PE=1 SV=3 | 2 | 1 | 1 | 1 | 504 | 56 | 8.53 | 2.33 | 1 | 1 |
| P39023 | 60S ribosomal protein L3 OS=Homo sapiens OX=9606 GN=RPL3 PE=1 SV=2 | 3 | 1 | 1 | 1 | 403 | 46.1 | 10.18 | 2.32 | 1 | 1 |
| P68400 | Casein kinase II subunit alpha OS=Homo sapiens OX=9606 GN=CSNK2A1 PE=1 SV=1 | 4 | 1 | 1 | 1 | 391 | 45.1 | 7.74 | 2.3 | 1 | 1 |
| Q15517 | Corneodesmosin OS=Homo sapiens OX=9606 GN=CDSN PE=1 SV=3 | 3 | 1 | 1 | 1 | 529 | 51.5 | 8.35 | 2.3 | 1 | 1 |
| O43242 | 26S proteasome non-ATPase regulatory subunit 3 OS=Homo sapiens OX=9606 GN=PSMD3 PE=1 SV=2 | 2 | 1 | 1 | 1 | 534 | 60.9 | 8.44 | 2.28 | 1 | 1 |
| Q8TF72 | Protein Shroom3 OS=Homo sapiens OX=9606 GN=SHROOM3 PE=1 SV=2 | 0 | 1 | 1 | 1 | 1996 | 216.7 | 7.8 | 2.28 | 1 | 1 |
| P39656 | Dolichyl-diphosphooligosaccharide--protein glycosyltransferase 48 kDa subunit OS=Homo sapiens OX=9606 GN=DDOST PE=1 SV=4 | 2 | 1 | 1 | 1 | 456 | 50.8 | 6.55 | 2.27 | 1 | 1 |
| P27348 | 14-3-3 protein theta OS=Homo sapiens OX=9606 GN=YWHAQ PE=1 SV=1 | 6 | 1 | 1 | 1 | 245 | 27.7 | 4.78 | 2.26 | 1 | 1 |
| P29401 | Transketolase OS=Homo sapiens OX=9606 GN=TKT PE=1 SV=3 | 3 | 1 | 1 | 1 | 623 | 67.8 | 7.66 | 2.26 | 1 | 1 |
| Q9P1U1 | Actin-related protein 3B OS=Homo sapiens OX=9606 GN=ACTR3B PE=2 SV=1 | 2 | 1 | 1 | 1 | 418 | 47.6 | 5.88 | 2.26 | 1 | 1 |
| Q9UBQ5 | Eukaryotic translation initiation factor 3 subunit K OS=Homo sapiens OX=9606 GN=EIF3K PE=1 SV=1 | 5 | 1 | 1 | 1 | 218 | 25 | 4.93 | 2.25 | 1 | 1 |
| O00231 | 26S proteasome non-ATPase regulatory subunit 11 OS=Homo sapiens OX=9606 GN=PSMD11 PE=1 SV=3 | 3 | 1 | 1 | 1 | 422 | 47.4 | 6.48 | 2.25 | 1 | 1 |
| P28331 | NADH-ubiquinone oxidoreductase 75 kDa subunit, mitochondrial OS=Homo sapiens OX=9606 GN=NDUFS1 PE=1 SV=3 | 2 | 1 | 1 | 1 | 727 | 79.4 | 6.23 | 2.25 | 1 | 1 |
| Q9NR31 | GTP-binding protein SAR1a OS=Homo sapiens OX=9606 GN=SAR1A PE=1 SV=1 | 6 | 1 | 1 | 1 | 198 | 22.4 | 6.68 | 2.24 | 1 | 1 |
| Q9H2U2 | Inorganic pyrophosphatase 2, mitochondrial OS=Homo sapiens OX=9606 GN=PPA2 PE=1 SV=2 | 4 | 1 | 1 | 1 | 334 | 37.9 | 7.39 | 2.23 | 1 | 1 |
| Q02790 | Peptidyl-prolyl cis-trans isomerase FKBP4 OS=Homo sapiens OX=9606 GN=FKBP4 PE=1 SV=3 | 3 | 1 | 1 | 1 | 459 | 51.8 | 5.43 | 2.22 | 1 | 1 |
| P33991 | DNA replication licensing factor MCM4 OS=Homo sapiens OX=9606 GN=MCM4 PE=1 SV=5 | 2 | 1 | 1 | 1 | 863 | 96.5 | 6.74 | 2.22 | 1 | 1 |
| Q01105 | Protein SET OS=Homo sapiens OX=9606 GN=SET PE=1 SV=3 | 4 | 1 | 1 | 1 | 290 | 33.5 | 4.32 | 2.21 | 1 | 1 |
| Q9H7E9 | UPF0488 protein C8orf33 OS=Homo sapiens OX=9606 GN=C8orf33 PE=1 SV=1 | 5 | 1 | 1 | 1 | 229 | 25 | 9.95 | 2.19 | 1 | 1 |
| Q92665 | 28S ribosomal protein S31, mitochondrial OS=Homo sapiens OX=9606 GN=MRPS31 PE=1 SV=3 | 2 | 1 | 1 | 1 | 395 | 45.3 | 9.29 | 2.19 | 1 | 1 |
| Q9Y520 | Protein PRRC2C OS=Homo sapiens OX=9606 GN=PRRC2C PE=1 SV=4 | 0 | 1 | 1 | 1 | 2896 | 316.7 | 9.13 | 2.19 | 1 | 1 |
| Q9Y5S9 | RNA-binding protein 8A OS=Homo sapiens OX=9606 GN=RBM8A PE=1 SV=1 | 10 | 1 | 1 | 1 | 174 | 19.9 | 5.72 | 2.18 | 1 | 1 |
| P98179 | RNA-binding protein 3 OS=Homo sapiens OX=9606 GN=RBM3 PE=1 SV=1 | 6 | 1 | 1 | 1 | 157 | 17.2 | 8.91 | 2.18 | 1 | 1 |
| P78417 | Glutathione S-transferase omega-1 OS=Homo sapiens OX=9606 GN=GSTO1 PE=1 SV=2 | 5 | 1 | 1 | 1 | 241 | 27.5 | 6.6 | 2.18 | 1 | 1 |
| P11166 | Solute carrier family 2, facilitated glucose transporter member 1 OS=Homo sapiens OX=9606 GN=SLC2A1 PE=1 SV=2 | 3 | 1 | 1 | 1 | 492 | 54 | 8.72 | 2.18 | 1 | 1 |
| P35998 | 26S proteasome regulatory subunit 7 OS=Homo sapiens OX=9606 GN=PSMC2 PE=1 SV=3 | 3 | 1 | 1 | 1 | 433 | 48.6 | 5.95 | 2.18 | 1 | 1 |
| P31948 | Stress-induced-phosphoprotein 1 OS=Homo sapiens OX=9606 GN=STIP1 PE=1 SV=1 | 2 | 1 | 1 | 1 | 543 | 62.6 | 6.8 | 2.18 | 1 | 1 |
| P49327 | Fatty acid synthase OS=Homo sapiens OX=9606 GN=FASN PE=1 SV=3 | 0 | 1 | 1 | 1 | 2511 | 273.3 | 6.44 | 2.18 | 1 | 1 |
| Q9H2W6 | 39S ribosomal protein L46, mitochondrial OS=Homo sapiens OX=9606 GN=MRPL46 PE=1 SV=1 | 5 | 1 | 1 | 1 | 279 | 31.7 | 7.05 | 2.17 | 1 | 1 |
| P06730 | Eukaryotic translation initiation factor 4E OS=Homo sapiens OX=9606 GN=EIF4E PE=1 SV=2 | 4 | 1 | 1 | 1 | 217 | 25.1 | 6.15 | 2.17 | 1 | 1 |
| P78406 | mRNA export factor OS=Homo sapiens OX=9606 GN=RAE1 PE=1 SV=1 | 2 | 1 | 1 | 1 | 368 | 40.9 | 7.83 | 2.17 | 1 | 1 |
| Q15758 | Neutral amino acid transporter B(0) OS=Homo sapiens OX=9606 GN=SLC1A5 PE=1 SV=2 | 2 | 1 | 1 | 1 | 541 | 56.6 | 5.48 | 2.17 | 1 | 1 |
| P31483 | Nucleolysin TIA-1 isoform p40 OS=Homo sapiens OX=9606 GN=TIA1 PE=1 SV=3 | 3 | 1 | 1 | 1 | 386 | 42.9 | 7.74 | 2.16 | 1 | 1 |
| Q52LJ0 | Protein FAM98B OS=Homo sapiens OX=9606 GN=FAM98B PE=1 SV=2 | 3 | 1 | 1 | 1 | 433 | 45.5 | 8.69 | 2.16 | 1 | 1 |
| P14324 | Farnesyl pyrophosphate synthase OS=Homo sapiens OX=9606 GN=FDPS PE=1 SV=4 | 2 | 1 | 1 | 1 | 419 | 48.2 | 6.15 | 2.16 | 1 | 1 |
| O75934 | Pre-mRNA-splicing factor SPF27 OS=Homo sapiens OX=9606 GN=BCAS2 PE=1 SV=1 | 5 | 1 | 1 | 1 | 225 | 26.1 | 5.66 | 2.15 | 1 | 1 |
| P04843 | Dolichyl-diphosphooligosaccharide--protein glycosyltransferase subunit 1 OS=Homo sapiens OX=9606 GN=RPN1 PE=1 SV=1 | 2 | 1 | 1 | 1 | 607 | 68.5 | 6.38 | 2.14 | 1 | 1 |
| Q9UQ35 | Serine/arginine repetitive matrix protein 2 OS=Homo sapiens OX=9606 GN=SRRM2 PE=1 SV=2 | 0 | 1 | 1 | 1 | 2752 | 299.4 | 12.06 | 2.14 | 1 | 1 |
| P15531 | Nucleoside diphosphate kinase A OS=Homo sapiens OX=9606 GN=NME1 PE=1 SV=1 | 8 | 1 | 1 | 1 | 152 | 17.1 | 6.19 | 2.12 | 1 | 1 |
| Q8N1G4 | Leucine-rich repeat-containing protein 47 OS=Homo sapiens OX=9606 GN=LRRC47 PE=1 SV=1 | 2 | 1 | 1 | 1 | 583 | 63.4 | 8.28 | 2.12 | 1 | 1 |
| P56182 | Ribosomal RNA processing protein 1 homolog A OS=Homo sapiens OX=9606 GN=RRP1 PE=1 SV=1 | 2 | 1 | 1 | 1 | 461 | 52.8 | 9.33 | 2.12 | 1 | 1 |
| O43823 | A-kinase anchor protein 8 OS=Homo sapiens OX=9606 GN=AKAP8 PE=1 SV=1 | 2 | 1 | 1 | 1 | 692 | 76.1 | 5.15 | 2.12 | 1 | 1 |
| Q08188 | Protein-glutamine gamma-glutamyltransferase E OS=Homo sapiens OX=9606 GN=TGM3 PE=1 SV=4 | 1 | 1 | 1 | 1 | 693 | 76.6 | 5.86 | 2.12 | 1 | 1 |
| P02794 | Ferritin heavy chain OS=Homo sapiens OX=9606 GN=FTH1 PE=1 SV=2 | 5 | 1 | 1 | 1 | 183 | 21.2 | 5.55 | 2.11 | 1 | 1 |
| O43684 | Mitotic checkpoint protein BUB3 OS=Homo sapiens OX=9606 GN=BUB3 PE=1 SV=1 | 4 | 1 | 1 | 1 | 328 | 37.1 | 6.84 | 2.11 | 1 | 1 |
| P31944 | Caspase-14 OS=Homo sapiens OX=9606 GN=CASP14 PE=1 SV=2 | 4 | 1 | 1 | 1 | 242 | 27.7 | 5.58 | 2.11 | 1 | 1 |
| Q13595 | Transformer-2 protein homolog alpha OS=Homo sapiens OX=9606 GN=TRA2A PE=1 SV=1 | 3 | 1 | 1 | 1 | 282 | 32.7 | 11.27 | 2.11 | 1 | 1 |
| P62258 | 14-3-3 protein epsilon OS=Homo sapiens OX=9606 GN=YWHAE PE=1 SV=1 | 4 | 1 | 1 | 1 | 255 | 29.2 | 4.74 | 2.1 | 1 | 1 |
| Q12904 | Aminoacyl tRNA synthase complex-interacting multifunctional protein 1 OS=Homo sapiens OX=9606 GN=AIMP1 PE=1 SV=2 | 4 | 1 | 1 | 1 | 312 | 34.3 | 8.43 | 2.1 | 1 | 1 |
| P35659 | Protein DEK OS=Homo sapiens OX=9606 GN=DEK PE=1 SV=1 | 3 | 1 | 1 | 1 | 375 | 42.6 | 8.56 | 2.1 | 1 | 1 |
| Q9Y2R5 | 28S ribosomal protein S17, mitochondrial OS=Homo sapiens OX=9606 GN=MRPS17 PE=1 SV=1 | 7 | 1 | 1 | 1 | 130 | 14.5 | 9.85 | 2.09 | 1 | 1 |
| Q02413 | Desmoglein-1 OS=Homo sapiens OX=9606 GN=DSG1 PE=1 SV=2 | 1 | 1 | 1 | 1 | 1049 | 113.7 | 5.03 | 2.09 | 1 | 1 |
| Q9ULR0 | Pre-mRNA-splicing factor ISY1 homolog OS=Homo sapiens OX=9606 GN=ISY1 PE=1 SV=3 | 5 | 1 | 1 | 1 | 285 | 33 | 5.17 | 2.08 | 1 | 1 |
| P17987 | T-complex protein 1 subunit alpha OS=Homo sapiens OX=9606 GN=TCP1 PE=1 SV=1 | 2 | 1 | 1 | 1 | 556 | 60.3 | 6.11 | 2.08 | 1 | 1 |
| O75083 | WD repeat-containing protein 1 OS=Homo sapiens OX=9606 GN=WDR1 PE=1 SV=4 | 1 | 1 | 1 | 1 | 606 | 66.2 | 6.65 | 2.08 | 1 | 1 |
| Q6UXG8 | Butyrophilin-like protein 9 OS=Homo sapiens OX=9606 GN=BTNL9 PE=1 SV=1 | 1 | 1 | 1 | 1 | 535 | 59.7 | 6.44 | 2.07 | 1 | 1 |
| P52565 | Rho GDP-dissociation inhibitor 1 OS=Homo sapiens OX=9606 GN=ARHGDIA PE=1 SV=3 | 7 | 1 | 1 | 1 | 204 | 23.2 | 5.11 | 2.06 | 1 | 1 |
| P61224 | Ras-related protein Rap-1b OS=Homo sapiens OX=9606 GN=RAP1B PE=1 SV=1 | 5 | 1 | 1 | 1 | 184 | 20.8 | 5.78 | 2.06 | 1 | 1 |
| Q6IS14 | Eukaryotic translation initiation factor 5A-1-like OS=Homo sapiens OX=9606 GN=EIF5AL1 PE=2 SV=2 | 5 | 1 | 1 | 1 | 154 | 16.8 | 5 | 2.06 | 1 | 1 |
| P23526 | Adenosylhomocysteinase OS=Homo sapiens OX=9606 GN=AHCY PE=1 SV=4 | 3 | 1 | 1 | 1 | 432 | 47.7 | 6.34 | 2.06 | 1 | 1 |
| P55209 | Nucleosome assembly protein 1-like 1 OS=Homo sapiens OX=9606 GN=NAP1L1 PE=1 SV=1 | 3 | 1 | 1 | 1 | 391 | 45.3 | 4.46 | 2.06 | 1 | 1 |
| P78371 | T-complex protein 1 subunit beta OS=Homo sapiens OX=9606 GN=CCT2 PE=1 SV=4 | 2 | 1 | 1 | 1 | 535 | 57.5 | 6.46 | 2.06 | 1 | 1 |
| O75533 | Splicing factor 3B subunit 1 OS=Homo sapiens OX=9606 GN=SF3B1 PE=1 SV=3 | 1 | 1 | 1 | 1 | 1304 | 145.7 | 7.09 | 2.06 | 1 | 1 |
| P10599 | Thioredoxin OS=Homo sapiens OX=9606 GN=TXN PE=1 SV=3 | 12 | 1 | 1 | 1 | 105 | 11.7 | 4.92 | 2.04 | 1 | 1 |
| Q9BU76 | Multiple myeloma tumor-associated protein 2 OS=Homo sapiens OX=9606 GN=MMTAG2 PE=1 SV=1 | 5 | 1 | 1 | 1 | 263 | 29.4 | 10.02 | 2.04 | 1 | 1 |
| O75477 | Erlin-1 OS=Homo sapiens OX=9606 GN=ERLIN1 PE=1 SV=2 | 3 | 1 | 1 | 1 | 348 | 39.1 | 7.87 | 2.04 | 1 | 1 |
| P13804 | Electron transfer flavoprotein subunit alpha, mitochondrial OS=Homo sapiens OX=9606 GN=ETFA PE=1 SV=1 | 3 | 1 | 1 | 1 | 333 | 35.1 | 8.38 | 2.04 | 1 | 1 |
| P61619 | Protein transport protein Sec61 subunit alpha isoform 1 OS=Homo sapiens OX=9606 GN=SEC61A1 PE=1 SV=2 | 2 | 1 | 1 | 1 | 476 | 52.2 | 8.06 | 2.04 | 1 | 1 |
| P13639 | Elongation factor 2 OS=Homo sapiens OX=9606 GN=EEF2 PE=1 SV=4 | 1 | 1 | 1 | 1 | 858 | 95.3 | 6.83 | 2.04 | 1 | 1 |
| Q96C36 | Pyrroline-5-carboxylate reductase 2 OS=Homo sapiens OX=9606 GN=PYCR2 PE=1 SV=1 | 5 | 1 | 1 | 1 | 320 | 33.6 | 7.77 | 2.03 | 1 | 1 |
| P98175 | RNA-binding protein 10 OS=Homo sapiens OX=9606 GN=RBM10 PE=1 SV=3 | 2 | 1 | 1 | 1 | 930 | 103.5 | 5.97 | 2.03 | 1 | 1 |
| P42704 | Leucine-rich PPR motif-containing protein, mitochondrial OS=Homo sapiens OX=9606 GN=LRPPRC PE=1 SV=3 | 1 | 1 | 1 | 1 | 1394 | 157.8 | 6.13 | 2.03 | 1 | 1 |
| O75369 | Filamin-B OS=Homo sapiens OX=9606 GN=FLNB PE=1 SV=2 | 0 | 1 | 1 | 1 | 2602 | 278 | 5.73 | 2.03 | 1 | 1 |
| P56385 | ATP synthase subunit e, mitochondrial OS=Homo sapiens OX=9606 GN=ATP5ME PE=1 SV=2 | 14 | 1 | 1 | 1 | 69 | 7.9 | 9.35 | 2.02 | 1 | 1 |
| O75964 | ATP synthase subunit g, mitochondrial OS=Homo sapiens OX=9606 GN=ATP5MG PE=1 SV=3 | 13 | 1 | 1 | 1 | 103 | 11.4 | 9.64 | 2.02 | 1 | 1 |
| Q9NUL3 | Double-stranded RNA-binding protein Staufen homolog 2 OS=Homo sapiens OX=9606 GN=STAU2 PE=1 SV=2 | 3 | 1 | 1 | 1 | 570 | 62.6 | 9.61 | 2.02 | 1 | 1 |
| P62249 | 40S ribosomal protein S16 OS=Homo sapiens OX=9606 GN=RPS16 PE=1 SV=2 | 8 | 1 | 1 | 1 | 146 | 16.4 | 10.21 | 2.01 | 1 | 1 |
| P30837 | Aldehyde dehydrogenase X, mitochondrial OS=Homo sapiens OX=9606 GN=ALDH1B1 PE=1 SV=3 | 3 | 1 | 1 | 1 | 517 | 57.2 | 6.8 | 2.01 | 1 | 1 |
| P31151 | Protein S100-A7 OS=Homo sapiens OX=9606 GN=S100A7 PE=1 SV=4 | 12 | 1 | 1 | 1 | 101 | 11.5 | 6.77 | 2 | 1 | 1 |
| P32969 | 60S ribosomal protein L9 OS=Homo sapiens OX=9606 GN=RPL9 PE=1 SV=1 | 5 | 1 | 1 | 1 | 192 | 21.9 | 9.95 | 1.99 | 1 | 1 |
| Q6PJT7 | Zinc finger CCCH domain-containing protein 14 OS=Homo sapiens OX=9606 GN=ZC3H14 PE=1 SV=1 | 2 | 1 | 1 | 1 | 736 | 82.8 | 7.31 | 1.99 | 1 | 1 |
| P78332 | RNA-binding protein 6 OS=Homo sapiens OX=9606 GN=RBM6 PE=1 SV=5 | 1 | 1 | 1 | 1 | 1123 | 128.6 | 6.32 | 1.99 | 1 | 1 |
| Q8TDP1 | Ribonuclease H2 subunit C OS=Homo sapiens OX=9606 GN=RNASEH2C PE=1 SV=1 | 8 | 1 | 1 | 1 | 164 | 17.8 | 5.03 | 1.98 | 1 | 1 |
| Q9UNZ5 | Leydig cell tumor 10 kDa protein homolog OS=Homo sapiens OX=9606 GN=C19orf53 PE=1 SV=1 | 7 | 1 | 1 | 1 | 99 | 10.6 | 11.55 | 1.98 | 1 | 1 |
| O14684 | Prostaglandin E synthase OS=Homo sapiens OX=9606 GN=PTGES PE=1 SV=2 | 7 | 1 | 1 | 1 | 152 | 17.1 | 9.5 | 1.98 | 1 | 1 |
| Q9H6F5 | Coiled-coil domain-containing protein 86 OS=Homo sapiens OX=9606 GN=CCDC86 PE=1 SV=1 | 4 | 1 | 1 | 1 | 360 | 40.2 | 10.33 | 1.97 | 1 | 1 |
| Q9BV38 | WD repeat-containing protein 18 OS=Homo sapiens OX=9606 GN=WDR18 PE=1 SV=2 | 2 | 1 | 1 | 1 | 432 | 47.4 | 6.7 | 1.97 | 1 | 1 |
| O14949 | Cytochrome b-c1 complex subunit 8 OS=Homo sapiens OX=9606 GN=UQCRQ PE=1 SV=4 | 12 | 1 | 1 | 1 | 82 | 9.9 | 10.08 | 1.96 | 1 | 1 |
| P21912 | Succinate dehydrogenase [ubiquinone] iron-sulfur subunit, mitochondrial OS=Homo sapiens OX=9606 GN=SDHB PE=1 SV=3 | 4 | 1 | 1 | 1 | 280 | 31.6 | 8.76 | 1.96 | 1 | 1 |
| P11310 | Medium-chain specific acyl-CoA dehydrogenase, mitochondrial OS=Homo sapiens OX=9606 GN=ACADM PE=1 SV=1 | 3 | 1 | 1 | 1 | 421 | 46.6 | 8.37 | 1.96 | 1 | 1 |
| O00330 | Pyruvate dehydrogenase protein X component, mitochondrial OS=Homo sapiens OX=9606 GN=PDHX PE=1 SV=3 | 2 | 1 | 1 | 1 | 501 | 54.1 | 8.66 | 1.96 | 1 | 1 |
| Q14697 | Neutral alpha-glucosidase AB OS=Homo sapiens OX=9606 GN=GANAB PE=1 SV=3 | 2 | 1 | 1 | 1 | 944 | 106.8 | 6.14 | 1.96 | 1 | 1 |
| Q9Y6V0 | Protein piccolo OS=Homo sapiens OX=9606 GN=PCLO PE=1 SV=5 | 0 | 1 | 1 | 1 | 5142 | 560.4 | 6.47 | 1.96 | 1 | 1 |
| P36542 | ATP synthase subunit gamma, mitochondrial OS=Homo sapiens OX=9606 GN=ATP5F1C PE=1 SV=1 | 3 | 1 | 1 | 1 | 298 | 33 | 9.22 | 1.95 | 1 | 1 |
| O15160 | DNA-directed RNA polymerases I and III subunit RPAC1 OS=Homo sapiens OX=9606 GN=POLR1C PE=1 SV=1 | 3 | 1 | 1 | 1 | 346 | 39.2 | 5.5 | 1.95 | 1 | 1 |
| Q9Y399 | 28S ribosomal protein S2, mitochondrial OS=Homo sapiens OX=9606 GN=MRPS2 PE=1 SV=1 | 3 | 1 | 1 | 1 | 296 | 33.2 | 9.26 | 1.95 | 1 | 1 |
| P60468 | Protein transport protein Sec61 subunit beta OS=Homo sapiens OX=9606 GN=SEC61B PE=1 SV=2 | 10 | 1 | 1 | 1 | 96 | 10 | 11.56 | 1.94 | 1 | 1 |
| Q99497 | Parkinson disease protein 7 OS=Homo sapiens OX=9606 GN=PARK7 PE=1 SV=2 | 5 | 1 | 1 | 1 | 189 | 19.9 | 6.79 | 1.94 | 1 | 1 |
| P41091 | Eukaryotic translation initiation factor 2 subunit 3 OS=Homo sapiens OX=9606 GN=EIF2S3 PE=1 SV=3 | 4 | 1 | 1 | 1 | 472 | 51.1 | 8.4 | 1.94 | 1 | 1 |
| Q01650 | Large neutral amino acids transporter small subunit 1 OS=Homo sapiens OX=9606 GN=SLC7A5 PE=1 SV=2 | 2 | 1 | 1 | 1 | 507 | 55 | 7.72 | 1.94 | 1 | 1 |
| P61326 | Protein mago nashi homolog OS=Homo sapiens OX=9606 GN=MAGOH PE=1 SV=1 | 8 | 1 | 1 | 1 | 146 | 17.2 | 6.11 | 1.93 | 1 | 1 |
| Q16527 | Cysteine and glycine-rich protein 2 OS=Homo sapiens OX=9606 GN=CSRP2 PE=1 SV=3 | 5 | 1 | 1 | 1 | 193 | 20.9 | 8.62 | 1.93 | 1 | 1 |
| Q92552 | 28S ribosomal protein S27, mitochondrial OS=Homo sapiens OX=9606 GN=MRPS27 PE=1 SV=3 | 2 | 1 | 1 | 1 | 414 | 47.6 | 6.18 | 1.93 | 1 | 1 |
| Q13200 | 26S proteasome non-ATPase regulatory subunit 2 OS=Homo sapiens OX=9606 GN=PSMD2 PE=1 SV=3 | 1 | 1 | 1 | 1 | 908 | 100.1 | 5.2 | 1.93 | 1 | 1 |
| P10515 | Dihydrolipoyllysine-residue acetyltransferase component of pyruvate dehydrogenase complex, mitochondrial OS=Homo sapiens OX=9606 GN=DLAT PE=1 SV=3 | 1 | 1 | 1 | 1 | 647 | 69 | 7.84 | 1.93 | 1 | 1 |
| Q9NZJ4 | Sacsin OS=Homo sapiens OX=9606 GN=SACS PE=1 SV=2 | 0 | 1 | 1 | 1 | 4579 | 520.8 | 7.05 | 1.93 | 1 | 1 |
| O95336 | 6-phosphogluconolactonase OS=Homo sapiens OX=9606 GN=PGLS PE=1 SV=2 | 6 | 1 | 1 | 1 | 258 | 27.5 | 6.05 | 1.92 | 1 | 1 |
| P47985 | Cytochrome b-c1 complex subunit Rieske, mitochondrial OS=Homo sapiens OX=9606 GN=UQCRFS1 PE=1 SV=2 | 5 | 1 | 1 | 1 | 274 | 29.6 | 8.32 | 1.92 | 1 | 1 |
| O75436 | Vacuolar protein sorting-associated protein 26A OS=Homo sapiens OX=9606 GN=VPS26A PE=1 SV=2 | 4 | 1 | 1 | 1 | 327 | 38.1 | 6.57 | 1.92 | 1 | 1 |
| O14929 | Histone acetyltransferase type B catalytic subunit OS=Homo sapiens OX=9606 GN=HAT1 PE=1 SV=1 | 3 | 1 | 1 | 1 | 419 | 49.5 | 5.69 | 1.92 | 1 | 1 |
| Q9H339 | Olfactory receptor 51B5 OS=Homo sapiens OX=9606 GN=OR51B5 PE=2 SV=2 | 3 | 1 | 1 | 1 | 312 | 35.2 | 8.31 | 1.92 | 1 | 1 |
| Q16891 | MICOS complex subunit MIC60 OS=Homo sapiens OX=9606 GN=IMMT PE=1 SV=1 | 1 | 1 | 1 | 1 | 758 | 83.6 | 6.48 | 1.92 | 1 | 1 |
| P63220 | 40S ribosomal protein S21 OS=Homo sapiens OX=9606 GN=RPS21 PE=1 SV=1 | 12 | 1 | 1 | 1 | 83 | 9.1 | 8.5 | 1.91 | 1 | 1 |
| Q9Y3C6 | Peptidyl-prolyl cis-trans isomerase-like 1 OS=Homo sapiens OX=9606 GN=PPIL1 PE=1 SV=1 | 8 | 1 | 1 | 1 | 166 | 18.2 | 7.99 | 1.91 | 1 | 1 |
| Q9UJV9 | Probable ATP-dependent RNA helicase DDX41 OS=Homo sapiens OX=9606 GN=DDX41 PE=1 SV=2 | 2 | 1 | 1 | 1 | 622 | 69.8 | 6.84 | 1.91 | 1 | 1 |
| Q9Y2W1 | Thyroid hormone receptor-associated protein 3 OS=Homo sapiens OX=9606 GN=THRAP3 PE=1 SV=2 | 1 | 1 | 1 | 1 | 955 | 108.6 | 10.15 | 1.91 | 1 | 1 |
| Q12797 | Aspartyl/asparaginyl beta-hydroxylase OS=Homo sapiens OX=9606 GN=ASPH PE=1 SV=3 | 3 | 1 | 1 | 1 | 758 | 85.8 | 5.01 | 1.9 | 1 | 1 |
| Q9BTD8 | RNA-binding protein 42 OS=Homo sapiens OX=9606 GN=RBM42 PE=1 SV=1 | 2 | 1 | 1 | 1 | 480 | 50.4 | 9.63 | 1.9 | 1 | 1 |

**Table S3. The information of primer sequences.**

| **Primer** | **Sequence (5’-3’)** |
| --- | --- |
| DDX3-K118R-For | CTTTGGCcgtTTTGAACGTGGTGGAAACAGTC |
| DDX3-K118R-Rev | GTTCAAAacgGCCAAAGCCACTTCTGTCACCA |
| DDX3-K130R-For | GTGTGACcgtTCAGATGAAGATGATTGGTCAAAAC |
| DDX3-K130R-Rev | CATCTGAacgGTCACACCAGCGACTGTTTCCA |
| DDX3-K208R-For | AGTGCAAcgtCATGCTATTCCTATTATCAAAGAGAAAA |
| DDX3-K208R-Rev | TAGCATGacgTTGCACTGGAGTTGGGCGAGTA |
| DDX3-K215R-For | TCcgtGAGAAAAGAGACTTGATGGCTTGTGCC |
| DDX3-K215R-Rev | GTCTCTTTTCTCacgGATAATAGGAATAGCATGCTTTTGCA |
| DDX3-K217R-For | CAAAGAGcgtAGAGACTTGATGGCTTGTGCCC |
| DDX3-K217R-Rev | AGTCTCTacgCTCTTTGATAATAGGAATAGCATGCTT |
| DDX3-K264R-For | CGCcgtCAATACCCAATCTCCTTGGTATTAGC |
| DDX3-K264R-Rev | ATTGGGTATTGacgGCGGCGCCCATACCTTCC |
| DDX3-K335R-For | GAAAGAGGAcgtATTGGATTAGACTTTTGCAAATACTTG |
| DDX3-K335R-Rev | CCAATacgTCCTCTTTCCATCATATCCACTAGAC |
| DDX3-K342R-For | CTTTTGCcgtTACTTGGTGTTAGATGAAGCTGATCG |
| DDX3-K342R-Rev | CCAAGTAacgGCAAAAGTCTAATCCAATCTTTCCT |
| DDX3-K373R-For | TACTATGCCTCCAcgtGGTGTCCGCCACACTATGATG |
| DDX3-K373R-Rev | CacgTGGAGGCATAGTATCTTGTTCGACTATT |
| DDX3-K387R-For | GCTACTTTTCCTcgtGAAATACAGATGCTGGCTCGTG |
| DDX3-K387R-Rev | TCacgAGGAAAAGTAGCACTAAACATCATAGTGT |
| DDX3-K418R-For | CACACAGcgtGTAGTTTGGGTGGAAGAATCAGACA |
| DDX3-K418R-Rev | AAACTACacgCTGTGTGATGTTTTCAGAGGTAGAGC |
| DDX3-K81R-For | AAGAGGGcgtTCTAGCTTCTTCAGTGATCGTGGA |
| DDX3-K81R-Rev | AGCTAGAacgCCCTCTTGAATCACTACGAGATCC |
| DDX3-K138R-For | ATTGGTCAcgtCCACTCCCACCAAGTGAACGC |
| DDX3-K138R-Rev | GAGTGGacgTGACCAATCATCTTCATCTGATTTG |
| DDX3-K491R-For | TCAGGAcgtAGCCCAATTTTAGTGGCTACAGC |
| DDX3-K491R-Rev | ATTGGGCTacgTCCTGAGCGGAACTGGTGAAG |
| DDX3-K511R-For | CAAATGTGcgtCATGTTATCAATTTTGACTTGCCAA |
| DDX3-K511R-Rev | AACATGacgCACATTTGAAATGTCCAGTCCTCT |
| DDX3-K230E-For | TGGAgagACTGCAGCATTTCTGTTGCCCATCT |
| DDX3-K230E-Rev | ATGCTGCAGTctcTCCAGACCCTGTTTGGGCA |
| DDX3-S382L-For | GATGTTTttgGCTACTTTTCCTAAGGAAATACAGATGC |
| DDX3-S382L-Rev | AAGTAGCcaaAAACATCATAGTGTGGCGGACA |
| DDX3(1-414)-For | gatgatgacgataaaggatccATGAGTCATGTGGCAGTGGAAA |
| DDX3(1-414)-Rev | aacgggccctctagactcgagGTTTTCAGAGGTAGAGCCAACTCTT |
| DDX3(1-544)-For | gatgatgacgataaaggatccATGAGTCATGTGGCAGTGGAAA |
| DDX3(1-544)-Rev | aacgggccctctagactcgagGAATGAGGTTGCCAGGCCA |
| DDX3(182-662)-For | gatgatgacgataaaggatccTTCAGTGATGTTGAGATGGGAGAA |
| DDX3(182-662)-Rev | aacgggccctctagactcgagTCAGTTACCCCACCAGTCAACC |
| DDX3(414-662)-For | gatgatgacgataaaggatccAACATCACACAGAAAGTAGTTTGGGT |
| DDX3(414-662)-Rev | aacgggccctctagactcgagTCAGTTACCCCACCAGTCAACC |
| Myc-Flag-TRIM28-For | aggagatctgccgccgcgatcgcATGGCGGCCTCCGCGGCG |
| Myc-Flag-TRIM28-Rev | tgagatgagtttctgctcgagTCAGGGGCCATCACCAGG |
| EGFP-TRIM28-For | gacgagctgtacaagggatccATGGCGGCCTCCGCGGCG |
| EGFP-TRIM28-Rev | aacgggccctctagactcgagTCAGGGGCCATCACCAGG |
| TRIM28-RBCC-For | gacgagctgtacaagggatccATGGCGGCCTCCGCGGCG |
| TRIM28-RBCC-For | aacgggccctctagactcgagGAGGGCCCGGTGCAGCTG |
| TRIM28-RING-For | gacgagctgtacaagggatccTGCGGCGTGTGCAGAGAG |
| TRIM28-RING-Rev | aacgggccctctagactcgagCTTGCACACGGGACAGTCCA |
| TRIM28-PHDBR-For | gacgagctgtacaagggatccAAGATGATTGTGGATCCCGTGG |
| TRIM28-PHDBR-Fev | aacgggccctctagactcgagACCGAAGGCCTCGTTCATG |
| TRIM28-PHD-For | gacgagctgtacaagggatccAAGATGATTGTGGATCCCGTGG |
| TRIM28-PHD-Rev | aacgggccctctagactcgagATCCAGGCTGAGGCTGCC |
| SENP1-C603A-For | AAGTGACgctGGGATGTTTGCCTGCAAATATG |
| SNEP1-C603A-Rev | ACATCCCagcGTCACTTCCATTCATCTGCTGAGG |
| EGFP-Pol-For | gacgagctgtacaagggatccATGCCCCTATCCTATCAACACTTC |
| EGFP-Pol-Rev | aacgggccctctagactcgagTCACGGTGGTCTCCATGCG |
| pgRNA-For | CTCAATCTCGGGAATCTCAATGT |
| pgRNA-Rev | TGGATAAAACCTAGCAGGCATAAT |
| IFNβ-For | ATGACCAACAAGTGTCTCCTCC |
| IFNβ-Rev | GCTCATGGAAAGAGCTGTAGTG |
| β-actin-For | CTCTTCCAGCCTTCCTTCCT |
| β-actin-Rev | AGCACTGTGTTGGCGTACAG |

Table S4. HCC tissue microarray information.

| **Tissue type** | **Gender** | **Age** | **Pathological grade** | **HBsAg** |
| --- | --- | --- | --- | --- |
| HCT/PLT | Female | 73 | Ⅱ | Positive |
| HCT/PLT | Female | 37 | Ⅱ | Positive |
| HCT/PLT | Male | 53 | Ⅱ | Positive |
| HCT/PLT | Male | 41 | Ⅲ | Positive |
| HCT/PLT | Female | 80 | Ⅱ | Negative |
| HCT/PLT | Male | 56 | Ⅱ | Positive |
| HCT/PLT | Male | 41 | Ⅱ | Positive |
| HCT/PLT | Male | 52 | Ⅱ-Ⅲ | Positive |
| HCT/PLT | Male | 49 | Ⅱ | Positive |
| HCT/PLT | Male | 50 | Ⅲ | Positive |
| HCT/PLT | Female | 43 | Ⅱ | Positive |
| HCT/PLT | Male | 63 | Ⅱ | Positive |
| HCT/PLT | Male | 43 | Ⅲ | Positive |
| HCT/PLT | Male | 38 | Ⅱ-Ⅲ | Positive |
| HCT/PLT | Male | 45 | Ⅲ | Positive |
| HCT/PLT | Female | 59 | Ⅱ | Positive |
| HCT/PLT | Male | 56 | Ⅱ | Positive |
| HCT/PLT | Male | 46 | Ⅱ | Positive |
| HCT/PLT | Male | 27 | Ⅱ | Positive |
| HCT/PLT | Male | 54 | Ⅱ | Positive |
| HCT/PLT | Male | 50 | Ⅱ | Positive |
| HCT/PLT | Male | 59 | Ⅱ | Negative |
| HCT/PLT | Male | 59 | Ⅱ-Ⅲ | Positive |
| HCT/PLT | Male | 44 | Ⅱ | Positive |
| HCT/PLT | Male | 39 | Ⅱ | Positive |
| HCT/PLT | Female | 70 | Ⅱ | Positive |
| HCT/PLT | Male | 51 | Ⅱ | Positive |
| HCT/PLT | Male | 54 | Ⅱ | Positive |
| HCT/PLT | Male | 48 | Ⅱ | Positive |
| HCT/PLT | Male | 35 | Ⅱ | Positive |
| HCT/PLT | Male | 53 | Ⅱ | Positive |
| HCT/PLT | Male | 48 | Ⅲ | Positive |
| HCT/PLT | Male | 46 | Ⅱ | Positive |
| HCT/PLT | Male | 29 | Ⅲ | Positive |
| HCT/PLT | Male | 62 | Ⅱ | Positive |
| HCT/PLT | Male | 57 | Ⅲ | Positive |
| HCT/PLT | Male | 79 | Ⅲ | Negative |
| HCT/PLT | Male | 39 | Ⅱ | Positive |
| HCT/PLT | Male | 39 | Ⅱ | Positive |
| HCT/PLT | Female | 55 | Ⅰ | Negative |
| HCT/PLT | Male | 59 | Ⅲ | Positive |
| HCT/PLT | Female | 60 | Ⅱ | Positive |
| HCT/PLT | Male | 59 | Ⅲ | Positive |
| HCT/PLT | Male | 75 | Ⅲ | Positive |
| HCT/PLT | Male | 66 | Ⅱ | Positive |
| HCT/PLT | Male | 34 | Ⅱ | Positive |
| HCT/PLT | Male | 42 | Ⅱ | Positive |
| HCT/PLT | Male | 41 | Ⅱ | Positive |
| HCT/PLT | Male | 57 | Ⅱ | Negative |
| HCT/PLT | Male | 59 | Ⅱ | Negative |
| HCT/PLT | Male | 51 | Ⅱ-Ⅲ | Positive |
| HCT/PLT | Male | 84 | Ⅱ | Positive |
| HCT/PLT | Male | 39 | Ⅲ | Positive |
| HCT/PLT | Male | 60 | Ⅲ | Positive |
| HCT/PLT | Male | 40 | Ⅲ | Positive |
| HCT/PLT | Male | 35 | Ⅲ | Positive |
| HCT/PLT | Female | 60 | Ⅲ | Positive |
| HCT/PLT | Male | 56 | Ⅱ | Positive |
| HCT/PLT | Male | 50 | Ⅱ | Positive |
| HCT/PLT | Male | 31 | Ⅱ | Positive |
| HCT/PLT | Male | 63 | Ⅲ | Positive |
| HCT/PLT | Female | 42 | Ⅲ | Positive |
| HCT/PLT | Male | 47 | Ⅱ | Negative |
| HCT/PLT | Male | 58 | Ⅲ | Positive |
| HCT/PLT | Female | 70 | Ⅱ | Negative |
| HCT/PLT | Male | 41 | Ⅱ-Ⅲ | Positive |
| HCT/PLT | Male | 72 | Ⅱ | Positive |
| HCT/PLT | Male | 64 | Ⅱ | Negative |
| HCT/PLT | Male | 49 | Ⅱ | Positive |
| HCT/PLT | Male | 50 | Ⅱ-Ⅲ | Negative |
| HCT/PLT | Female | 72 | Ⅱ | Positive |
| HCT/PLT | Male | 54 | Ⅲ | Positive |
| HCT/PLT | Male | 39 | Ⅲ | Positive |
| HCT/PLT | Male | 48 | Ⅱ | Positive |
| HCT/PLT | Male | 60 | Ⅰ-Ⅱ | Positive |
| HCT/PLT | Female | 56 | Ⅲ | Positive |
| HCT/PLT | Female | 45 | Ⅱ | Positive |
| HCT/PLT | Male | 55 | Ⅱ | Positive |
| HCT/PLT | Male | 43 | Ⅱ | Positive |
| HCT/PLT | Female | 64 | Ⅱ | Positive |
| HCT/PLT | Male | 51 | Ⅲ | Negative |
| HCT/PLT | Male | 69 | Ⅱ | Positive |
| HCT/PLT | Female | 62 | Ⅱ | Positive |
| HCT/PLT | Male | 45 | Ⅱ | Positive |
| HCT/PLT | Male | 58 | Ⅱ | Positive |
| HCT/PLT | Male | 64 | Ⅱ | Negative |
| HCT/PLT | Male | 42 | Ⅲ | Positive |
| HCT/PLT | Male | 49 | Ⅱ | Positive |
| HCT/PLT | Male | 41 | Ⅱ-Ⅲ | Positive |
| HCT/PLT | Male | 55 | Ⅲ | Positive |
|  |  |  |  |  |
| HCT, HCC tissue | | | | |
| PLT, patients’ autologous para-cancerous liver tissue | | | | |
